# Medical Gas Therapy for Tissue, Organ, and CNS Protection: A Systematic Review of Effects, Mechanisms, and Challenges

**DOI:** 10.1002/advs.202104136

**Published:** 2022-03-04

**Authors:** Ross D. Zafonte, Lei Wang, Christian A. Arbelaez, Rachel Dennison, Yang D. Teng

**Affiliations:** ^1^ Department of Physical Medicine and Rehabilitation Harvard Medical School Boston MA 02115 USA; ^2^ Neurotrauma Recovery Research, Department of Physical Medicine and Rehabilitation Spaulding Rehabilitation Hospital Network, Mass General Brigham, and Harvard Medical School Boston MA 02129 USA; ^3^ Spaulding Research Institute Spaulding Rehabilitation Hospital Network Boston MA 02129 USA; ^4^ Laboratory of SCI, Stem Cell and Recovery Neurobiology Research, Department of Physical Medicine and Rehabilitation Spaulding Rehabilitation Hospital Network, Mass General Brigham, and Harvard Medical School Boston MA 02129 USA

**Keywords:** carbon monoxide, cell signaling, functional recovery, medical gas therapy, spinal cord injury, tissue protection, traumatic brain injury, xenon

## Abstract

Gaseous molecules have been increasingly explored for therapeutic development. Here, following an analytical background introduction, a systematic review of medical gas research is presented, focusing on tissue protections, mechanisms, data tangibility, and translational challenges. The pharmacological efficacies of carbon monoxide (CO) and xenon (Xe) are further examined with emphasis on intracellular messengers associated with cytoprotection and functional improvement for the CNS, heart, retina, liver, kidneys, lungs, etc. Overall, the outcome supports the hypothesis that readily deliverable “biological gas” (CO, H_2_, H_2_S, NO, O_2_, O_3_, and N_2_O) or “noble gas” (He, Ar, and Xe) treatment may preserve cells against common pathologies by regulating oxidative, inflammatory, apoptotic, survival, and/or repair processes. Specifically, CO, in safe dosages, elicits neurorestoration via igniting sGC/cGMP/MAPK signaling and crosstalk between HO‐CO, HIF‐1*α*/VEGF, and NOS pathways. Xe rescues neurons through NMDA antagonism and PI3K/Akt/HIF‐1*α*/ERK activation. Primary findings also reveal that the need to utilize cutting‐edge molecular and genetic tactics to validate mechanistic targets and optimize outcome consistency remains urgent; the number of neurotherapeutic investigations is limited, without published results from large in vivo models. Lastly, the broad‐spectrum, concurrent multimodal homeostatic actions of medical gases may represent a novel pharmaceutical approach to treating critical organ failure and neurotrauma.

## Graphical abstract

This systematic review is focused on tissue protections, mechanisms, data qualities, and translational challenges of experimental gas therapies. The outcome suggests that biological/noble gases in safe dosages preserve cells by modulating oxidative, inflammatory, apoptotic, survival, and/or repair processes; it remains urgent to validate therapeutic targets via cutting‐edge molecular/genetic technologies. Gas agents may be developed to treat organ failure and neurotrauma.

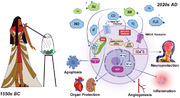

## 1 Review Design, Methods, Databases, and Results

**Figure 1:**
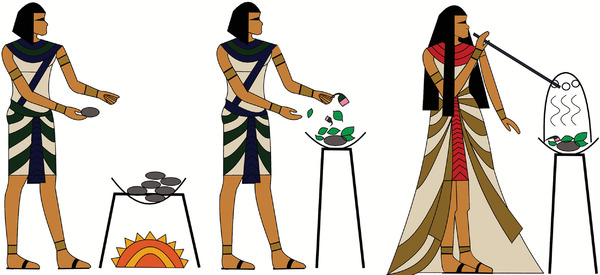
The ancient Egyptian papyrus (so called “Ebers papyrus”: ≈1554 BC) recorded the oldest known description of therapeutic aerosol delivery (i.e., smoke of henbane plants was administered through the stalk of a reed). The schematic illustration showed an Egyptian caregiver preheated bricks (left) before throwing the weed onto them (middle). The vaporized liquid of black henbane plants that contained the tropane alkaloids such as atropine was then inhaled by a patient to relieve breathing stress (right). Redrawn from ref. [191].

A narrative review of literature collected from PubMed and Google Scholar was conducted to analytically compose the historical background of medical gas therapy (MGT; see **Figure** **1** for an example), with hand searches and authoritative texts used to verify cited information. We next performed a systematic review to test *the central hypothesis* that medical gases might possess efficacy for tissue preservation via activating specific signaling pathways to promote repair and recovery.^[^ ^1^ ^]^ For the formulated literature search, we sought articles (published between January 1, 2000 – July 31, 2021) on experimental mechanistic investigations of medical gases as therapeutics, with relevant clinical reports crosschecked to assess translational potential. Keywords comprising Brain, Medical Gas, Neural, Protection, Preservation, Regeneration, Repair, Spinal Cord, etc. plus their varied combinations (Section S1: Supporting Information) were used to search 1) Medline, a bibliographic database of life sciences and biomedical information produced by The US National Library of Medicine;^[^ ^2^ ^]^ 2) Scopus, an abstract and citation database covering topics of life sciences, social sciences, physical sciences, and health sciences provided by Elsevier;^[^ ^3^ ^]^ and 3) Embase, a biomedical and pharmacological bibliographic database produced by Elsevier that includes published literature designed to support compliance with the regulatory requirements of a licensed drug.^[^ ^4^ ^]^

**Figure 2:**
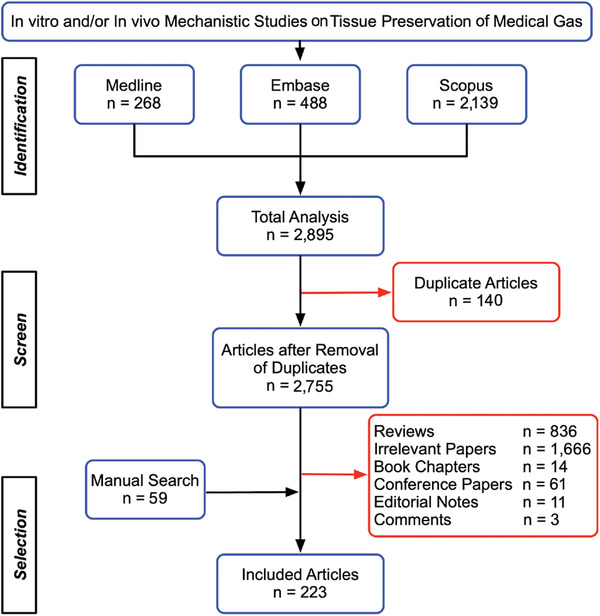
The publication enrollment process and outcome regarding in vitro and/or in vivo mechanistic investigations of medical gas‐induced tissue protections. In total, 223 papers were systematically qualified for further analysis (see all enrolled articles in Section S2: Supporting Information).

Literature tracking and selection were facilitated by using Boolean Operators (i.e., AND, OR, NOT or AND NOT) as conjunctions to combine or exclude keywords.^[^ ^5^ ^]^ All results were reconfirmed before conducting further evaluation (see **Figure** **2** and **Table** **1** for details). Search strategy and included publications were shown in Sections S1 and S2 (Supporting Information), respectively. After eliminating duplicates through Endnote X9 (Clarivate Analytics), titles and abstracts of identified articles were independently evaluated by two authors to screen for qualified reports that described in vitro and/or in vivo studies about the effect of medical gases on tissue protection and underlying mechanisms (**Table** **2**; abbreviations, acronyms, and references in Section S3: Supporting Information). Book chapters, conference abstracts/papers, reviews, editorial notes, and comments were excluded. Furthermore, cited references and “similar papers” automatically selected by PubMed as a qualified article were also screened and manually crosschecked at Google Scholar for additional includible reports prior to compiling the final bibliography.

**Table 1:** Medical subject headlines or relevant keywords utilized for literature searches

Search limits	(1) Publications from January 1, 2000 – July 31, 2021 (2) Literature published in English	
Database	Ovid‐Medline/Embase	Scopus
Medical subject headings (MeSH) or key words concerning tissue protection	Angioplasty, Bone regeneration, Bone protection, Brain regeneration, Brain repair, Liver regeneration, Nerve regeneration, Neural repair, Neuroprotection, Neural protection, Neuroregeneration, Regeneration, Spinal cord regeneration, Spinal cord repair, Tissue preservation, Tissue repair, Tissue protection.	Tissue preservation, Tissue protection, Tissue repair.
Medical subject headings (MeSH) or key words concerning medical gas	Argon, Carbon monoxide, Gases, Helium, Hydrogen, Hydrogen sulfide, Argon, Krypton, Medical gas, Nitric oxide, Nitrous oxide, Noble gas, Oxygen, Ozone, Xenon.	Argon, Carbon monoxide, Helium, Hydrogen sulfide, Hydrogen therapy, Argon, Krypton, Medical gas, Nitric oxide, Nitrous oxide, Noble gas, Oxygen therapy, Ozone, Xenon.
Medical subject headings (MeSH) or key words concerning mechanism	Mechanism, Pathway, Signaling, Signal transduction.	Mechanism, Pathway, Signal transduction.

Note: see Sections S1 and S2: Supporting Information, for database search strategies and enrolled articles, respectively.

**Table 2:** Representative tissue protective effects of medical gases

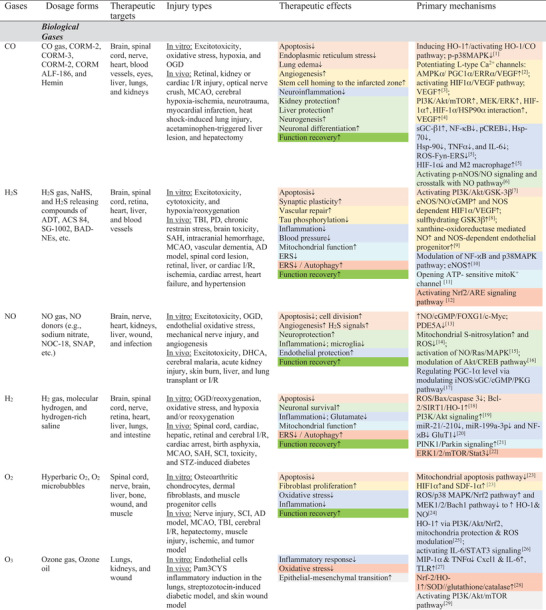					
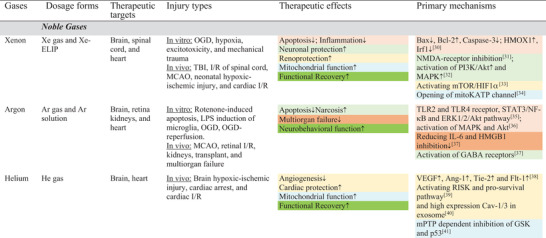					

Note: Abbreviations and citations in Sections S3: Supporting Information.

In total, we retrieved 268 studies from Medline, 488 studies from Embase, and 2139 from Scopus. A total of 2755 papers emerged after removing 140 duplicates. After further evaluation, 223 publications, including 59 reports chosen manually, met inclusion criteria (Figure 2). All qualified papers were subsequently grouped into three categories: 1) biological gases (i.e., gaseous molecules that are produced by live cells to modulate biological activities); 2) noble gases (i.e., the six natural elements that comprise Group 18 [VIIIa; traditionally labeled Group 0]) of the periodic table; and 3) reports on findings of adverse effects or no significant impact of medical gases on some or all outcome measures (Table S1: Supporting Information).

**Figure 3:**
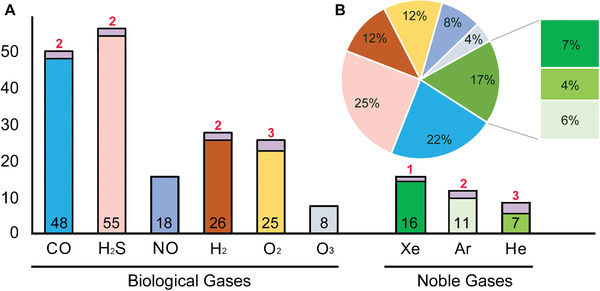
Histogram and pie chart of classification and quantification of all enrolled reports on mechanistic investigation of gas‐mediated tissue protection. A) numbers of reports showing discernible therapeutic effect (black numbers inside the bars) were much higher than those exhibiting ineffectiveness and/or side effect (red numbers on top of the bars) for all gases examined (note: no negative reports were found for NO and O_3_). B) The percentages in the chart corresponded to the number of positive reports of each gas over the total number of papers enrolled (i.e., 223). The majority of the qualified studies demonstrating therapeutic efficacy investigated biological gases (total: 83%) of hydrogen sulfide (H_2_S: 55/223 = 25%) and carbon monoxide (CO: 48/223 = 22%), with xenon (Xe: 16/223 = 7%) being the most studied noble gas (group total: 17%).

**Figure 4:**
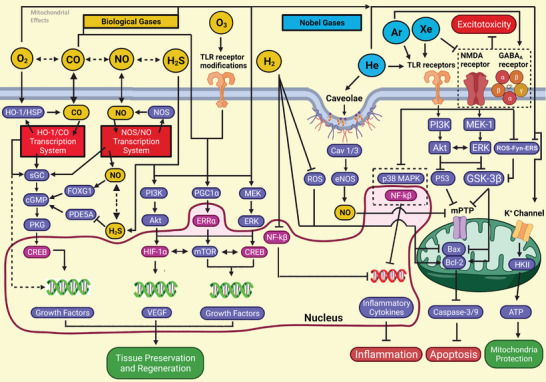
Schematic diagrams of diverging and converging mechanisms underlying the tissue protection and repair effects of medical gases. Biological gases (top left), in general, effect via modulating HO1/HSP/L‐type Ca^2+^ channels, NOS, sGC, PI3K/Akt, MEK, and PGC1*α* to regulate gene expressions of CREB/mTOR, growth factors, and HIF‐1*α*/VEGF. Also involved are TLRs that affect p38 MAPK and NF‐*κ*B to impede proinflammatory cytokines, as well as ROS and GSK‐3*β* to modulate mPTP/cytochrome *c* and Bax/Bcl2/caspases. Conversely, noble gases (top right) work by agonizing GABA_A_ receptors and antagonizing NMDA receptors, ROS, and mPTP/mitochondrial K^+^ channels to ameliorate excitotoxicity, caspase activation, and cell death. More specifics can be found in the text regarding the signaling pathways of each gas (note: definitions of abbreviations and acronyms are listed in Sections S3 and S4: Supporting Information).

**Figure 5:**
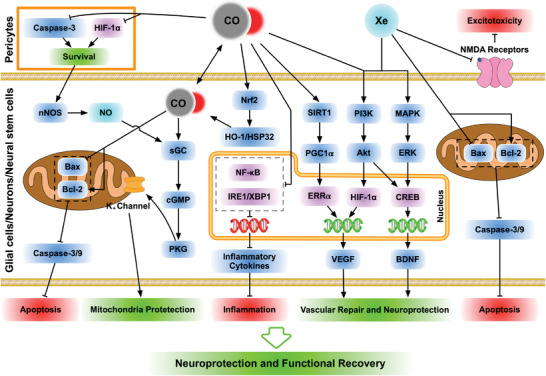
Schematic diagrams of diverging and converging mechanisms underlying the neural protective and recovery effects of carbon monoxide (CO) and xenon (Xe). Left panel: CO impedes IRE1 and NF‐*κ*B via activating Nrf2/HO‐1/HSP in neural cells including neural stem cells (↓inflammatory mediators), and suppresses HIF‐1*α* and caspases in pericytes (↓cell death). Moreover, CO exerts therapeutic effects through igniting NOS/sGC/PKG to regulate Bax/Bcl2/caspases to augment cell survival, and activating SIRT1/PI3K/MAPK to upregulate VEGF and BDNF. Right panel: Xe protects and repairs neural tissue by antagonizing NMDA receptors to mitigate excitotoxicity, and stimulating PI3K/MAPK, which partly overlaps with the mechanisms of CO. Detailed signaling pathways and target cells are presented in the text and definitions of abbreviations/acronyms in Sections S3 and S4: Supporting Information.

Biological gases reviewed were carbon monoxide (CO), hydrogen (H_2_), hydrogen sulfide (H_2_S), nitric oxide (NO), oxygen (O_2_), and ozone (O_3_). Noble gases evaluated consisted of helium (He), argon (Ar), and xenon (Xe). Because of interstudy differences in experimental models, outcome measures, drug deliveries, and research designs, statistical comparison (e.g., meta‐analysis) between studies regarding medical gas efficacy for a particular tissue was deemed biologically invalid. Instead, based on an established rule for systematic reviews, quantitative distributions of all enrolled publications by MG type were displayed in **Figure** **3** (histograms and a pie chart) to show the number of research reports on each medical gas describing the cytoprotective effects that met the qualification criteria; also generated were Tables 2–4 (summarizing typical MG effects of tissue protection, mechanisms and functional recovery data), Section S2: Supporting Information (publications cited in the tables), Tables S1–S4: Supporting Information (about negative results, experimental models/gas dosages, statistical methods/sample size/power analysis, and data pre‐processing procedures, respectively), and a section on statistics analysis to assess research outcome tangibility. Analysis of these outcomes generated a narrative synthesis of intervention impacts of medical gases, which was utilized to qualitatively test the central hypothesis. Major mechanisms underlying the therapeutic effect of the gases were illustrated in **Figure** **4** and Figure S1: Supporting Information (the key signal transduction pathways and molecular mechanisms underlying representative tissue protective effects reviewed in Table 2). Reports about investigations on the therapeutic impacts of CO and Xe were reviewed in **Table** **3** and Table **4**, respectively. The mechanisms of CO and Xe on neural protection and recovery were detailed in **Figure** **5**. Finally, for concision, only standard abbreviations of signaling molecules and other biological terms were used (see Sections S3 and S4: Supporting Information for full length nomenclatures, abbreviations, and acronyms).

**Table 3:** Neuroprotective mechanisms of carbon monoxide (CO)

Author/Year	Species and cells	Insults	Treatment	Duration	Effect	Mechanisms
	In Vitro					
Choi, Y. K. 2010^[1]^	Human primary brain astrocytes	Inhibition and knockdown of signaling pathways	CORM‐2: 5‐100 µM	8 h with or w/o drugs affecting signaling	Angiogenesis↑	PI3K/Akt/mTOR↑, MEK/ERK↑, HIF‐1*α*↑, HIF‐1*α*/HSP90*α* interaction↑, VEGF↑
Dallas, M. L. 2011^[2]^	Rat primary hippocampal neurons	Oxidative stress	CORM‐2: 30 µM or 100 µM CO‐saturated solution (1:5)	1 h exposure after injury	Apoptosis↓	Inhibition of K_v_2.1 via PKG/ERK pathway
Dreyer A., N. 2018^[3]^	Human neural stem cells and neurospheres	None (survival and development assay)	CO: 12.5–100 ppm for cells, 25 ppm for neurospheres	30 min exposure on day 0 and 4	Neurodifferentiation↑ Cell apoptosis↓	MAP2↑, HIF1*α*↑, mitochondrial alterations, and caspase 3↓
Imuta, N. 2007^[4]^	Rat primary cerebral neurons	Hypoxia	CO: 2.5‐5%	0‐24 h under hypoxia	Neuronal survival↑	cGMP↑ and caspase 3↓
Lin, C. 2017^[5]^	Rat astrocytes	IL‐1*β* induced inflammation	CORM‐3: 30 µM	Pretreatment for 4 h	Neuroinflammation↓	c‐Src/Pyk2/PKC*α*/ERK1/2↑, cFos↑, c‐Jun ↑, HO‐1↑, MMP9↓
Vieira, H. L. 2008^[6]^	Mouse primary cerebellar granule cells	Excitotoxic and oxidative stress	CO: 250 ppm	1.5 h exposure before injury	Neuronal survival↑	HO‐1/CO↑ and NO/cGMP/mitoK_ATP_↑
Horvat, A. 2017^[7]^ ^a)^	Rat astrocytes	Noradrenaline induction	CO: 100 µM solution	10 min before induction	Interfering aerobic glycolysis/glycogenolysis	Intracellular L‐lactate↓
	In Vivo					
Biermann, J. 2010^[8]^	Rat	Retinal I/R injury	Inhalation of CO (250 ppm)	1 h exposure before injury	Apoptosis↓ Inflammation↓	DNA binding of HSF‐1 and CREB↑, caspase 3↓, p‐p38MAPK↑, Hsp‐70↑, and TNF*α*↓
Han, Y. 2015^[9]^	Rat	Recurrent febrile seizures	Intraperitoneal injection of hemin (40 mg kg^−1^)	30 min before seizure induction	Apoptosis↓ ERS↓	HO‐1/CO↑, GRP78↑, CHOP↓, and PERK/eIF2↑
Ulbrich, F. 2016^[10]^	Rat	Retinal I/R injury	Intravenous injection of CORM ALF‐186 (10 mg kg^−1^)	After reperfusion	Apoptosis↓	p‐p38MAPK↑, ERK1/2↓, caspase 3↓, Bax↓, and Bcl‐2↑
Ulbrich, F. 2017^[11]^	Rat	Retinal I/R injury	Intravenous injection of CORM ALF‐186 (10 mg kg^−1^)	After reperfusion	Apoptosis↓ Inflammation↓	sGC‐*β*1↑, NF‐*κ*B↓, pCREB↓, Hsp‐70↓, Hsp‐90↑, TNF*α*↓, and IL‐6↓
Wang, B. 2011^[12]^	Mouse	MCAO	Inhalation of CO (250 ppm)	18 h exposure after MCAO	Infarct size↓ Neurobehavioral function↑	OH‐1 expression↑ and nuclear translocation of Nrf2↑
Wang, R. 2018^[13]^	Rat	Optic nerve crush	Inhalation of CO (250 ppm)	1 h exposure before injury	Retinal ganglion cell survival↑	Caspase‐9↓ and caspase 3↓
Zheng, G. 2020^[14]^	Rat	Vascular clip (30 g x 1 min) thoracic level 9 spinal cord compression	Intravenous injection of CORM‐3 (8 mg kg^−1^)	Immediately after SCI plus daily dose over the whole study	Blood spinal cord barrier disruption↓ Neutrophil infiltration↓ Neurobehavioral function↑	Tight junction preservation (Zo‐1), NF‐*κ*B signaling↓, MMP‐9↓
	In Vitro and In Vivo					
Choi, Y. K. 2016^[15]^	In Vitro: Human brain pericytes/Embryonic NSCs/ Rat adult NSCs	OGD	CORM‐3: 200 µM	Treatment for 24 h after OGD	Cell death↓ and Vascular integrity↑	Prorepair activities of pericytes and NSCs↑ and proneurogenesis p‐nNOS/NO signaling between pericytes and NSCs↑
	In vivo: Mouse	TBI	Intravenous injection of CORM‐3 (4 mg kg^−1^) or inhalation of CO (250 ppm)	1 h after injury (bolus injection or 1 h inhaling)	Neurogenesis↑ and Neurodifferentiation↑	
Choi, Y. K. 2017^[16]^	In vitro: Human astrocytes, HBMECs or HIF1*α*‐deficient RCCs	Knockdown of HO‐1, AMPKa, PGC‐1*α*, CaMKK*β*, ERR*α*, or/and SIRT1	Serum deprivation plus RuCl_3_ (200 µM) or CORM‐2 (100 µM); CORM‐2 (25 µM) and bilirubin (25 µM)	8 h with or w/o drugs affecting signaling	HO‐1‐derived CO triggered HIF1*α*‐independent VEGF↑	Activating L‐type Ca 2+channels, AMPK*α*/NAMPT/SIRT1/PGC1*α*/ERR*α* pathway↑, VEGF↑
	In vivo: Wildtype and HO‐1^+/‐^ mouse	I/R by 2 h MCAO and 24 h reperfusion		None		
Kaiser, S. 2020^[17]^	In vitro: Wildtype, HO‐1‐ and CD36‐deficient mouse microglia	Blood‐exposure	CO: 250 ppm	Immediately after blood‐exposure	Erythrophagocytosis↑ Neuronal apoptosis↓ Neurobehavioral function↑	HO‐1/CO↑, ROS↑, pAMPK↑, CD36↑
	In vivo: mouse	Subarachnoid hemorrhage model	O: 250 ppm	Immediately after injury for 1h plus daily exposure for 7 days		
Lu, K. 2020^[^ ^18^ ^]^	In vitro: mouse microglia BV2 cell line	*β*‐ionizing radiation	CORM‐3: 100 µM	30 mins before injury	Microglia activation↓ Inflammation↓ Neuronal apoptosis↓ Neurogenesis↑	p‐p38 MAPK↓, ICAM‐1↓, iNOS↓
	In vivo: mouse	Brain injury induced by *β*‐ionizing radiation	Intraperitoneal injection of CORM‐3 (40 mg kg^−1^)	Immediately after injury plus daily dose for 7 days		
Schallner, N. 2013^[19]^	In vitro: Human neuroblastoma cells	Rotenone‐induced apoptosis	CORM ALF186: 10–100 mM	30 mins before injury	Apoptosis↓ RGC survival↑	sGC/cGMP/PKG1↑, caspase 3↓, Bax↓, Bcl‐2↑
	In vivo: Rat	Retinal I/R injury (1 h ischemia and retina collection at 7 d after I/R)	Intravenous injection of CORM ALF186 10 mg kg^−1^	30 mins before injury		
Stifter, J. 2017^[20]^	In vitro: Rat retinal explant In vivo: Rat	Retinal I/R injury (1 h ischemia and retina collection at 24 h) Retinal I/R injury (1 h ischemia and retina collection at 2 or 7 d after I/R)	Intravitreal injection of CORM ALF‐186 (25 µg)	Immediately after injury	RGC survival↑ Axon regeneration↑ GFAP+ cell migration↑ and differentiation↑ Inflammation↓ Apoptosis↓	p‐p38 MAPK↑, GAP‐43↑, *β*III‐tubulin↑, and nestin↑ Hsp‐70↓, Hsp‐90↑, NF‐*κ*B↓, TNF*α*↓, AIF1↓, and caspase 3↓
Queiroga, C.S. 2012^[^ ^21^ ^]^	In vitro: Rat primary cerebellar granule cells	Excitotoxic stress	10 µM CO solution	1 h culture before insult	Apoptosis↓	Mitochondrial release of cytochrome c↓, caspase 3↓, and Bcl‐2↑
	In vivo: Rat	Neonatal cerebral hypoxia‐ischemia	Inhalation of CO 250 ppm	1 h exposure in 3 days before injury		
Zheng, G. 2019^[^ ^22^ ^]^	In vitro: Rat primary neuron In vivo: Rat	OGD Vascular clip (30 g x 1 min) thoracic level 9 spinal cord compression	The CORM‐3:100 µM Intravenous injection of CORM‐3 (8 mg kg^−1^)	Before OGD Immediately after injury plus daily dose throughout the study (28 days)	Inflammation↓ Neuron death↓ Neurobehavioral function↑	ERS‐medicated pyroptosis and inflammasome signaling↓ (e.g., IRE1/XBP1↓, GSDMD↓, IL1*β*↓, IL18↓, caspase 1↓, and caspase 11↓)

^a)^ This is a special review that contained original research data elucidating potential neuroprotective mechanisms of CO; Note: Abbreviations and reference citations in Sections S3: Supporting Information.

**Table 4:** Neuroprotective mechanisms of xenon (Xe) (Abbreviations and reference citations in Sections S3: Supporting Information)

Author/Year	Species and cells	Insult	Treatment	Duration	Effect	Mechanisms
	In Vitro					
Harris, K. 2013^[1]^	Mouse organotypic hippocampal slices	Focal mechanical injury to CA1 via stylus drop	0.5 ATM Xe (or Ar) in a hyperbaric chamber (1.5 ATM) immediately after injury	30 min‐24 h after injury	Cell secondary injury processes↓ Cell death scale↓	Inhibition of the glycine site of the NMDA receptor and activation of TREK‐1 channels by Xe
Koziakova, M. 2019^[2]^	Mouse organotypic hippocampal slices	OGD for 30 min	0.5 ATM Xe (or Ar) in a hyperbaric chamber (1.5 ATM) started 10 min after OGA	24 h exposure after injury	Cell death scale↓	Inhibition of the glycine site of the NMDA receptor by Xe
Lavaur, J. 2017^[3]^	Rat midbrain DA neurons and astrocytes	PDC‐excitotoxic exposure for 1 or 4 days, or spontaneous cell death in defined culture condition	75% Xe/25% O_2_	1 or 4 days (overlapping with PDC exposure)	Neuronal survival↑ Oxidative stress↓	Blockade of NMDA receptors by Xe (for excitotoxicity), and mitigate harmful effect from proliferating astrocytes and/or direct trophic effect of Xe (for spontaneous cell death)
Petzelt, C. 2003^[4]^	Rat cortical neurons	Hypoxia for 0‐2 h	50% (50% O_2_) or 100% Xe	0‐2 h (overlapping with hypoxia)	Neuronal damage (assessed by LDH) ↓ Neuronal survival↑ Hypoxia‐triggered glutamate release↓	Blockade of NMDA receptor, and possibly improving CaMKII‐mediated Ca^2+^‐dependent modification of neurotransmitter dynamics
Petzelt, C. 2004^[5]^	Rat PC12 cells (differentiated to DA neuron‐like cells)	Hypoxia for 0‐2 h	100% Xe	0‐2 h (overlapping with hypoxia)	Neuronal damage (assessed by LDH) ↓ Cell survival↑ Hypoxia‐triggered dopamine release↓	Possibly improving CaMKII‐mediated Ca^2+^‐dependent intracellular modifications
	In Vivo					
Limatola, V. 2010^[6]^	Mouse	Transient MCAO (1 h)	Inhalation of 70% Xe/30% O_2_	2 h exposure before injury	Infarct size↓ Functional recovery↑	HIF1*α*↑ and p‐Akt↑
Liu, S. 2016^[7]^	Rat	Spinal cord I/R injury	Inhalation of 50% Xe/50% O_2_	1 h exposure after injury	Apoptosis↓ Motor neuron survival↑ Functional recovery↑	p‐Akt↑ and p‐ERK↑
Peng, T. 2013^[8]^	Rat	Transient MCAO (2 h)	Intraarterial injection of Xe‐ELIP (7 mg kg^−1^)	2, 3 & 5 h after MCAO	Infract size↓ Functional recovery↑	Activation of MAPK and Akt, and BDNF↑ (Optimal effect observed at 3 h post MCAO)
Yang, Y. W. 2014^[9]^	Rat	Spinal cord I/R injury	Inhalation of 50% Xe/50% O_2_	1 h exposure overlapping with reperfusion	Neuronal survival↑ Apoptosis↓ Functional recovery↑	Cyt c↓, caspase 3↓, Bax↓ and Bcl‐2↑
Zhuang, L. 2012^[10]^	Rat	Hypoxic–ischemic injury via right common carotid artery ligation (for 90 ∼120 min)	Inhalation of 70% Xe/30% O_2_	1.5 h exposure after injury	Neuronal survival↑ Infract size↓ Functional recovery↑	Bax↓ and Bcl‐2↑
Filev, A. D. 2021^[11]^	Rat	Unilateral weight‐drop brain injury (50g x 10cm)	Inhalation of 70‐75% Xe/25‐30% O_2_	15‐30 min after TBI for 1 h	Contralateral: ↑stress genes (Irf1, Hmox1, S100A8, & S100A9) Infarct area: ↓Irf1, an inflammatory gene	
	In Vitro and In Vivo					
Ma, D. 2005^[12]^	In vitro: Mixed 1‐2 PND mouse cortical glial‐neuronal cocultures	OGD	12.5‐75% Xe plus mild hypothermia (20‐33 °C)	24 h (overlapping with OGD)	Necrosis↓ Apoptosis↓	NMDA antagonism, Bax↓, Bcl‐2↑, and caspase 3↓ (for both treated in vitro and in vivo models)
	In vivo: Rat (7 PND)	Hypoxic–ischemic injury (same as above)	Inhalation of 20‐70% Xe plus mild hypothermia (33 °C)	90 min (during hypoxia) or 2‐24 h after hypoxia	Viable neuron↑ Neuronal apoptosis↓ Functional recovery↑ (significant protection seen at 4 h post insult)	
Luo, Y. 2008^[13]^	In vitro: (1) Mixed 1‐2 PND mouse cortical glial‐neuronal cocultures (2) E16 mouse cortical neurons	OGD (for 24 h)	Effective doses: 50% and 75% Xe or 12.5 Xe plus 0.67% Sevoflurane (for 2 h)	2 h exposure before injury	Necrosis↓ Apoptosis↓ Viable cells↑	Activation of PI3K/Akt/CREB signaling pathway in both in vitro and in vivo treated groups
	In vivo: Rat (7 PND)	Hypoxic–ischemic injury (same as above) starting at 4 h after pretreatment of medical gases	Inhalation of 20% or 75% Xe (effective), 0.75% or 1.5% (effective) sevoflurane, or 20% Xe plus 0.75% sevoflurane (effective) for 2 h	4 h before injury	Infarct size↓ Functional recovery↑	

Note: Abbreviations and reference citations in Section S3: Supporting Information.

## 2 Historical Background of Applying Gases for Therapeutic Purposes

### 2.1 Introduction

Modern medical gases are conventionally described as compressed gases that are used in clinical procedures involving symptom treatment and anesthesia, or driving and conditioning medical devices or tools. Commonly utilized in hospitals are six individual gases (i.e., O_2_, N_2_, N_2_O, Ar, He, and CO_2_) and three gaseous mixtures (i.e., Entonox: 50% N_2_O and 50% O_2_; Heliox: 79% He and 21% O_2_; and compressed air).^[^ ^8^ ^]^ Therefore, in this review, medical gases are defined as pharmaceutical gaseous molecules that fulfill the therapeutic demands of specific pathophysiologic conditions.

On account of the relatively short history of industrialized production of gas compounds,^[^ ^9^ ^]^ MGT has been considered a contemporary clinical discipline. However, our analysis suggested that the origin and evolvement of inhalation of medicinal aerosols likely provided the foundation for MGT development.

### 2.2 Historical Perspectives

More than two thousand years before gaseous molecules were artificially purified, inhalation therapy had been invented. Its original purpose was to allow medications to swiftly reach the diseased respiratory, and other systems.^[^ ^10^ ^]^ The first known reference to inhaled therapeutics was recorded in an Egyptian medical papyrus scroll (purchased in the winter of 1873–74 by Georg Ebers at Luxor, Egypt) dating back to ≈1554 BCE.^[^ ^10^, ^11^ ^]^ It was described in the papyrus that patients with breathing stress were prescribed to inhale the vapor of black henbane (*Hyoscyamus niger*) plants baked on a hot brick through a stalk of reed that was inserted into a dome jar (Figure 1). As black henbane contains alkaloids such as atropine, hyoscyamine, scopolamine, and tropane, its aerosol accordingly has bronchodilating, antisecretory, urinary bladder relaxant, spasmolytic, hypnotic, sedative, and anti‐diarrheal properties.^[^ ^12^ ^]^

Even earlier, the most frequently consumed ventilatory drug probably already existed: the smoking of opium for therapeutic (e.g., analgesia, treatment of diarrhea, severe cough, etc.) and recreational purposes, based on documentation written around 1100 BCE.^[^ ^13^ ^]^ In addition, claimed accounts of asthma were being recorded around 2698–2598 BCE in China, and the medicinal herb *Ephedra sinica* (麻黄), containing ephedrine, was prescribed to treat it via smoke, steam vapor, and aromatic sachets.^[^ ^10^, ^14^ ^]^ Inhalation of various herb vapors (including tobacco) and resins, sometimes boiled with vinegar and oil, was also widely practiced in other early civilizations beginning in India, Ancient Greece, the Middle East, and North and Central America.^[^ ^9^ ^]^

### 2.3 Emergence of MGT

It was not until the discovery of oxygen in the late 18^th^ century that physicians began integrating MGT into clinical practice with what might be called a modern signature. Carl Wilhelm Scheele first identified oxygen in 1771 and mentioned the discovery in a letter to Antoine Lavoisier in 1774 before submitting his finding for publication in 1775 (in press by 1777). Also published in 1774 and 1775 was characterization work of oxygen performed independently by Joseph Priestly.^[^ ^15^, ^16^ ^]^ In contrast to other gases identified earlier or at a similar time (i.e., CO_2_, H_2_, and NO and N_2_O, in 1754, 1766, and 1772, respectively) that were adapted to help patients much later, the therapeutic potential of O_2_ was speculated soon after its discovery.

In 1783, benefits of O_2_ inhalation on tuberculosis was observed by M. Caillens.^[^ ^17^ ^]^ Ten years afterward, Dr. Beddoes started using O_2_ for asthma, tuberculosis, chronic lung disease, palsy, dropsy, and various other conditions.^[^ ^18^ ^]^ In the early 1800s, O_2_ was recognized as the essential element that humans obtain through respiration. Over the following 200 years, therapeutic administration of O_2_ and other gases fluctuated but, in general, increased remarkably over time. Toward the middle of the 20^th^ century, O_2_ therapy finally became rational and scientific.^[^ ^19^ ^]^

Standard acute therapies for patients utilize 24–100% O_2_ in inspired air (relative to 20.95% in the natural dry air at sea level) under normobaric and hyperbaric conditions.^[^ ^20^ ^]^ It is worth noting that, more recently, intermittent hypoxia (i.e., episodic exposure to O_2_ levels lower than normoxia) has been under intensive investigation for its potential in tissue protection, neuroplasticity, and functional improvement.^[^ ^21^ ^]^ The establishment of this unconventional approach was aided by advances in the understanding of molecular specifics underlying cell stress, trophic factor expression, survival and death, and the tissue sparing effect of other gaseous molecules as secondary messengers (see below).^[^ ^22^, ^23^ ^]^

## 3 MGT Investigation for Tissue Protection

### 3.1 Biological Gases

To understand these naturally occurring molecules for their multiplicity of action and interaction with biological targets to modulate cell survival and function, research efforts were first focused on determining the dose range of a particular gas that was able to ignite a set of signaling events to produce a specific biological effect not only in gas‐generator cells, but also in cells not synaptically coupled with them.^[^ ^24^ ^]^

#### 3.1.1 Carbon Monoxide (CO)

Joseph Priestley was credited for the isolation of CO during 1772–1799.^[^ ^16^ ^]^ Despite its lethal poisoning potency in high concentrations (human adjusted 0.5 h [h] LC: 4000 ppm (CF: 1); cdc.gov/niosh/idlh/630 080.htm), a low level of CO has been known to exist in the blood of healthy humans and animals since the late 19^th^ century.^[^ ^25^ ^]^ In 1968, Tenhunen, Marver, and Schmidt determined that heme oxygenase (HO) was the rate‐limiting enzyme to catabolize heme degradation to produce CO, ferrous iron, and biliverdin; biliverdin‐reductase then converted biliverdin to bilirubin.^[^ ^26^ ^]^ CO, bilirubin, and biliverdin were later found to have antioxidative and cell protective qualities.^[^ ^27^, ^28^, ^29^ ^]^ CO, in particular, could multimodally affect neurotransmission, vasodilation, and suppression of platelet aggregation.^[^ ^28^ ^]^ Accordingly, HO‐1 and CO have been deemed crucial endogenous homeostatic, signaling, and cell protective agents with desirable therapeutic value.^[^ ^29^ ^]^

Biochemically, HO belongs to the heat shock protein (HSP) family as a heme‐containing member named HSP32. In mammals, there are three isoforms of HO: an inducible form of HO‐1 that is produced throughout the body, two constitutively expressed forms of HO‐2 in the brain, testes, and endothelial cells of other organs, and HO‐3, a heme‐binding sensor due to its catalytic inactivity.^[^ ^30^ ^]^ HO is mainly located in the endoplasmic reticulum (ER), as well as the cell nucleus, mitochondria, and plasma membrane.^[^ ^30^ ^]^ Heme has high affinity to NO, CO, and O_2_, and operates as a gas sensor and signal transducer.^[^ ^31^ ^]^ For both prokaryotes and eukaryotes, gas sensing is pivotal for survival and function. The heme‐based sensor proteins such as HO and NPAS2 thereupon play important roles in regulating cellular responses to gaseous environment changes (e.g., O_2_, CO, NO, and H_2_S) through coupling a “regulatory” heme binding site to a “functional” signal‐transmitter site.^[^ ^32^ ^]^ Distinctly, CO, NO, and H_2_S are members of the growing family of molecules termed “gasotransmitters” that influence functional, metabolic, and genetic events.^[^ ^33^, ^34^ ^]^

The human body's average rate of CO generation is ≈20 µmol h^−1^, resulting in the normal baseline human carboxyhemoglobin level of 0.4–1%. The activity of HO accounts for 86–80% of endogenous CO production. The remaining 14–20% is derived from nonheme sources (e.g., hepatic catabolism of hemoproteins and ineffective erythropoiesis in bone marrow).^[^ ^35^ ^]^ CO, in physiological concentrations (e.g., 0.5–0.7 nmol mg^−1^ protein/liver; ≈20 pmol mg^−1^ protein/brain astrocytes,^[^ ^36^ ^]^) acts as a ubiquitous second messenger affecting a wide range of physiological and pathophysiological processes. This concept appears to have been widely accepted,^[^ ^34^, ^37^ ^]^ albeit with some disputes (e.g., CO primarily stimulates mitochondria to produce ROS that subsequently activate secondary messengers).^[^ ^38^ ^]^ Challenged by stress (e.g., inflammation), cells increase HO and CO production to restore homeostasis for tissue protection.^[^ ^38^, ^39^ ^]^ To date, most reports have shown that safe dosage CO inhalation therapy reduced cell death in the brain, spinal cord, heart, retina, kidneys, lungs, etc. and enhanced functional recovery in established models of trauma, cerebral ischemia, myocardial infarction, ischemia‐reperfusion injury (IRI), etc. (Table 2; Section S3: Supporting Information).^[^ ^40^, ^41^, ^42^, ^43^, ^44^, ^45^ ^]^

In male Wistar rats, CO‐donor methylene chloride (500 mg kg^−1^, p.o.) or HO‐1 inducer cobalt protoporphyrin IX (5 mg kg^−1^, i.p.) was administered at 24 and 3 h, respectively, before coronary artery ligation (3 min).^[^ ^45^ ^]^ Compared to controls, pretreatment of CO donor activated stem/progenitor cells to migrate into the infarct area to differentiate into vascular smooth muscle cells. The healing process was associated with augmented expression of HIF‐1*α*, SDF‐1*α*, and VEGF‐B, molecules impacting stem/progenitor cell migration, differentiation, vasculogenesis, and myocardial regeneration. Additionally, heightened HO‐1 production improved SDF‐1*α* (a progenitor mobilizer) expression and vascular density,^[^ ^45^ ^]^ suggesting that CO and HO‐1 cooperatively evoked required mechanisms for cardiac repair.

In terms of safety, experimental in vivo inhalations typically utilized 100–500 ppm CO for bolus administrations with or without consecutive exposures (e.g., 1–2 h/session for one to ≥10 days [d]). Animals all tolerated these exposures well without discernible toxic responses (Table 2; Section S3: Supporting Information). Clinical evidence has been corroborative, demonstrating that transient exposure to CO (≤500 ppm) did not have long lasting effects on the brain or other organs. Hence, relatively healthy individuals are safe to undergo short duration (i.e., several h/d) CO treatments at low doses.^[^ ^46^ ^]^ In contrast, in people with coronary artery disease histories, low concentrations of CO inhalation (i.e., 117–225 ppm × 50–70 min) exacerbated myocardial ischemia during graded physical exercise.^[^ ^46^ ^]^

In a small size pilot clinical study (*n* = 9; healthy male; age: 19 – 40 y/o), synthetic air (placebo) or 500 ppm CO was given for 1 h, followed by a safe bolus of LPS, an inflammation inducer (2 ng kg^−1^/each person, i.v.). Compared to the experimental anti‐inflammatory results shown in Table 2, the CO regimen, however, did not significantly reduce LPS‐elevated plasma concentrations of proinflammatory cytokines.^[^ ^47^ ^]^ The discrepancy might be attributable to pharmacological differences: animals had higher LPS doses (e.g., 9 ng kg^−1^) plus CO treatment both pre‐ and post‐LPS injection without body fluid maintenance, whereas human volunteers received systemic hydration protection, a 78% lower dose of LPS, and post‐LPS CO inhalation only.^[^ ^47^ ^]^

Since the early 2000s, synthetic pharmaceuticals which release CO have been invented to enhance targeting, controlled release, and potential systemic toxicity prevention; the precision level of these pharmacological parameters will be further optimized by the development of gas‐producing nanoplatforms.^[^ ^47^, ^48^ ^]^ Different types of CO‐releasing molecules (CORMs) have been experimentally investigated.^[^ ^49^, ^50^, ^51^ ^]^ In a rat acute lung injury model induced by hemorrhagic shock and resuscitation where interstitial macrophage demise was detrimental, hydrophilic CORM‐3 (4 or 8 mg kg^−1^, i.v., given immediately after resuscitation) significantly ameliorated lung injury, pulmonary edema, macrophage apoptotic or pyroptotic death and expressions of p‐p38MAPK.^[^ ^52^ ^]^ When given at 1 h after acetaminophen‐induced liver injury in mice, lipophilic CORM‐2 (5 mg kg^−1^, i.v.) suppressed M1 (proinflammation)/M2 (pro‐healing) ratios of microglia polarization by decreasing HIF‐1*α* and increasing HO‐1 to reduce oxidative damage and inflammation. The treatment also promoted liver regeneration by activating hepatic progenitor cells via igniting the PI3K/Akt/mTOR pathway.^[^ ^52^ ^]^

The data suggested that, unlike conventional cytoprotectants (e.g., trophic factors) that exclusively act through specific receptors, CO, under varied pathogenic milieus, could differentially affect the same type of target cells (e.g., macrophages: programmed death or polarization) by concurrently modulating multiple second messengers to promote homeostasis and repair (e.g., progenitor/stem cell mobilization). Conversely, to attain a direct antioxidant effect on a murine acute kidney injury model, a pretreatment of CORM‐2 (30 mg kg^−1^, i.p.) done 2 h before LPS injection (15 mg kg^−1^, i.p.) appeared necessary in order to impede ROS‐Fyn‐ER stress signaling in nephric cells.^[^ ^52^ ^]^ Notably, CO exhibited a partly similar mechanistic profile when effecting as a neurotherapeutic (see below).^[^ ^53^ ^]^

A more recent report showed that CO administered via inhalation (250 ppm for 1 h before injury), i.p. (CO prodrug BW‐101: 100 mg kg^−1^, i.p.), or p.o. (CO liquid HBI‐002: 0.2 mg kg^−1^), was nephroprotective in a model of bilateral kidney IRI in mice through modulating purinergic signaling. The treatment augmented CD39 ectonucleotidase expression, reduced expression of Adora1 (adenosine receptor A1); there was a concomitant increase in Adora2a/2b. These changes were associated with marked elevations of the circadian rhythm protein Period 2 (Per2) and serum erythropoietin (EPO), which jointly impeded kidney IRI (see details in CO reference #7 in Section S2: Supporting Information).

Also demonstrated was that either CO treatment or HO‐1 elevation induced by endogenous heme increased cellular CO production,^[^ ^54^ ^]^ suggesting that augmentation of endogenous CO generation is an important therapeutic mechanism. However, HO‐1 induction is a time‐consuming process (e.g., 6–12 h by CORM‐3 in rat brain astrocytes;^[^ ^55^ ^]^ 12–24 h by heme in human monocytes;^[^ ^56^ ^]^ and 3–24 h by IL‐10 in a dose‐dependent way.^[^ ^57^ ^]^) This fact has made administration of CO or CORM a valuable approach to managing acute tissue or organ lesions.^[^ ^58^ ^]^

Three pathways have been identified to mediate the medicinal effect of CO in addition to its multifaceted antistress and anti‐inflammation mechanisms (e.g., ↓p38MAPK;^[^ ^52^ ^]^ ↑IRG1/↓TNF*α* ^[^ ^59^ ^]^): modulation of the HO‐CO axis, HIF‐1*α* dependent and independent induction of VEGF expression, and crosstalk with NO and other messengers. Among them, activation of the HO‐CO system was determined as a major player in rallying multiple mechanisms of tissue repair (Table 2; Section S3: Supporting Information; Figure 4; Figure S1: Supporting Information). For example, CO stimulated angiogenesis, a crucial effector of tissue preservation by building the following positive feedback loops: 1) triggering translational activation and stabilization of HIF‐1*α*; 2) imposing a “pseudo” intracellular hypoxia environment that upregulated VEGF expression to increase circulating endothelial progenitor cells; 3) reducing endothelial apoptosis via activating PERK, which is a key transducer of ER stress and cell death/survival regulation;^[^ ^45^, ^59^, ^60^ ^]^ 4) inducing production of hypoxia‐responsive microRNA‐101, which, via stabilizing Nrf2, promoted Nrf2‐dependent HO‐1 synthesis to augment VEGF and eNOS‐derived NO levels;^[^ ^61^ ^]^ and 5) modulating L‐type Ca^2+^ channel activity to either activate AMPK*α*/SIRT1‐PGC‐1*α*/ERR*α* signaling to increase VEGF, or reduce Ca^2+^ influx.^[^ ^41^ ^]^ Importantly, CO‐evoked crosstalk with NO signaling pathway has been shown to mobilize NSCs (for neurotherapeutic specifics, see Sections 4.1., 4.3.1. and 4.3.2.).^[^ ^44^, ^62^ ^]^

#### 3.1.2 Hydrogen Sulfide (H_2_S)

Hydrogen sulfide (H_2_S) was also discovered by Carl Wilhelm Scheele in 1777.^[^ ^63^ ^]^ Commonly produced from the microbial process of anaerobic digestion, volcanic gases, etc., H_2_S had occupationally been regarded as a toxic, corrosive, and flammable hazard.^[^ ^64^ ^]^ However, since Stipanuk and Beck reported that small quantities of H_2_S were produced by human cells in 1982,^[^ ^65^ ^]^ H_2_S has been investigated and gained acceptance as a gasotransmitter and neuromodulator.^[^ ^66^ ^]^

Apart from its production by the intestinal microbiota, H_2_S is regularly generated from various sources in mammalian tissues by specific enzymes (e.g., cystathionine‐*β*‐synthase, cystathionine‐*γ*‐lyase, and 3‐mercaptosulfurtransferase).^[^ ^66^, ^67^ ^]^ H_2_S exercises important roles in intra‐ and intercellular communication in mammals. Due to lipid‐solubility, gasotransmitters are not regulated by vesicle‐mediated storage and release transitions. Instead, their signals are gated by metabolism‐ and/or physical diffusion‐controlled concentration fluctuations. In this manner, H_2_S, working in consortium with NO, modulates ureteral smooth muscle activity in urodynamics and affects relaxation of the sphincters.^[^ ^67^ ^]^

Exogenous H_2_S and its donor (NaHS) have been examined for their tissue protection potential in the brain, spinal cord, heart, retina, blood vessels, etc., against excitotoxicity, neurotrauma, heart failure, and IRI.^[^ ^68^, ^69^, ^70^ ^]^ Data suggested that H_2_S treatment strengthened tissue repair through modulating specific signaling pathways. Specifically, H_2_S induced HIF‐1*α* and VEGF expression through both NOS dependent and independent pathways (e.g., nitrite reduction activity; see below), which partially overlapped with CO pathways (Figure 4; Figure S1: Supporting Information; Table 2), to promote revascularization in a murine chronic tissue ischemia model.^[^ ^70^ ^]^ The effect of H_2_S to increase VEGF and cytokeratin 10 expression in keratinocytes could promote wound healing.^[^ ^70^ ^]^ H_2_S acted via upregulating xanthine‐oxidoreductase to catalyze nitrite reduction to NO. NO in turn increased HIF‐1*α* and VEGF expression that dose‐dependently stimulated chronic ischemic vascular remodeling.^[^ ^70^ ^]^ In a heart failure model, H_2_S therapy resulted in cardioprotection by activating the eNOS‐NO‐cGMP and Akt‐VEGF pathways in addition to preserving mitochondrial function to comprehensively enhance angiogenesis and temper oxidative stress.^[^ ^71^ ^]^

For neuroprotection, H_2_S pretreatment (released from 200 µmol L^−1^ NaSH for 30 min) protected rat pheochromocytoma PC12 cells, which were used to proximate neurons, against scratch‐caused decrease of cystathionine‐*β*‐synthetase, a key enzyme for H_2_S generation, and attenuated depolarization of mitochondrial membrane, intracellular accumulation of ROS, and cell death. The effects were diminished by blocking the PI3K/Akt pathway with LY294002 (5 × 10^−6^ m x 24 h).^[^ ^72^ ^]^ H_2_S exposure (40 or 80 ppm × 1 h, given immediately after cardiac arrest and cardiopulmonary resuscitation in rats) impeded inflammation and oxidative stress by inhibiting NF‐*κ*B activation and downstream proinflammatory mediators iNOS and ICAM‐1 to improve hippocampal neuron protection, neurological recovery, and animal survival.^[^ ^73^ ^]^ Recently, it was reported that CSE/cystathionase, another crucial biosynthetic enzyme of H_2_S, failed to bind Tau P301L, a mutant Tau related to AD pathogenesis. CSE‐produced H_2_S normally prevents hyperphosphorylation of Tau by sulfhydrating its kinase GSK3*β*. In both 3xTg‐AD mice and in human AD brains, CSE was depleted. Treating the AD mice (6 m/o) with NaGYY, a slow‐releasing H_2_S donor (100 mg kg^−1^, i.p.; q.d. × 12 weeks) mitigated motor and cognitive deficits through sulfhydrating GSK3*β*.^[^ ^73^ ^]^

Targets of H_2_S also included Bcl‐2 and surviving for cell survival; cyclin D1, mTOR, Bax, and caspases for cell death; IL‐6 and IL‐12 for immune and inflammatory responses; and GABA_A_ receptor for neuroplasticity.^[^ ^64^, ^74^ ^]^ Taken together, H_2_S may divergently (e.g., via Akt‐mediated events to enhance cell survival and function) and convergently (e.g., through NF‐*κ*B signaling to impede detrimental inflammation) regulate cell survival, death, homeostasis, and function (Figure 4; Figure S1: Supporting Information). Contrary to the conventional concept that H_2_S as a tissue messenger must maintain an intratissue concentration range of 30 – ≥100 × 10^−6^ m, a study uncovered an average intratissue H_2_S level of ≈15 × 10^−9^ m due to rapid catabolism, and ≈100 × 10^−6^ m was required for H_2_S to affect cellular function.^[^ ^75^ ^]^ If reconfirmed, future investigations should try to maintain an effective dose of H_2_S only in the target region to mitigate side effects, because H_2_S can lesion and kill cells by overly perturbating the same set of intracellular messengers.^[^ ^64^, ^74^, ^75^, ^76^ ^]^

#### 3.1.3 Nitric Oxide (NO)

Nitric Oxide (NO; also termed nitrogen oxide or nitrogen monoxide) was perhaps first encountered by Jan Baptista van Helmont around 1620.^[^ ^77^ ^]^ It was, however, Joseph Priestly who made NO widely recognized.^[^ ^16^, ^78^ ^]^ Though amyl nitrite–a compound that metabolizes into NO–was first medically applied in 1867 to treat chest pain, it was replaced a decade later by nitroglycerin, one of the first synthetic drugs, for administration convenience and longer duration of effectiveness.^[^ ^79^ ^]^ The effect of NO to potently improve coronary blood flow was recognized in the 1920s. NO‐releasing drugs have since been administered as vascular relaxants to save lives.^[^ ^79^, ^80^ ^]^

The role of NO as a gaseous messenger was not validated until the 1980s.^[^ ^81^ ^]^ When administering NMDA, an agonist of a glutamate receptor subtype, to rat hippocampal slices, researchers noticed that NO concentrations in the CA1 and CA3 pyramidal cell layer were increased from ≈2–10 to ≈200 × 10^−9^ m. Furthermore, for any newly elevated NO doses to facilitate neuronal long‐term potentiation, a tonic endogenous low concentration of NO was required, suggesting that cell production of NO was dependent on the functional level of the glutamate NMDA receptor. Since NMDA receptors mediate excitatory neurotransmission that detonates important signaling cascades to regulate neurogenesis, development, neuronal plasticity, neural cell interaction, senescence, cell death, and disease, the finding validated a role of NO in modulating these processes.^[^ ^24^ ^]^ NO was subsequently determined to be a controller of vascular tone and integrity, inflammatory response of endothelial cells and leukocytes, and platelet function.^[^ ^80^ ^]^

By 1991, Frostell et al. reported that inhalation of NO in low doses reduced pulmonary artery pressure (PAP) in an unanesthetized lamb pulmonary arterial hypertension (PAH) model.^[^ ^82^ ^]^ However, NO is a free radical when possessing an unpaired electron. The feature makes it highly reactive with the thiol/sulfhydryl groups that usually exist in disulfide linkages to sustain the tertiary and quaternary structures of proteins. Consequently, NO inhalation has not been approved for therapeutic application in most organ disorders, except for those of the lungs (e.g., PAH and term or near‐term neonates with hypoxemia and PAH) and heart (e.g., angina pectoris) in short‐term and low concentration (i.e., ≤80 ppm) formulas to limit its action range and harmful effect.^[^ ^80^, ^82^, ^83^, ^84^, ^85^ ^]^ NO dose‐dependently reduces PAP and pulmonary vascular resistance for PAH but not for pulmonary circulation systems in normal vascular tone.^[^ ^79^, ^80^, ^82^, ^83^, ^84^, ^85^ ^]^

Appreciated recently were three molecular mechanisms underlying the cytoprotective effect of NO in low doses (Figure 4; Figure S1: Supporting Information). First, NO activated sGC, which then increased cGMP and ignited the PKG pathway to regulate PGC‐1*α* synthesis to mediate cardiovascular and other tissue protection or destruction (e.g., the microbiocidal viricidal effects of NO), depending on the dose level and time course of NO exposure.^[^ ^86^ ^]^ Second, NO modified protein thiol/sulfhydryl residues via *S*‐nitrosylation, exerting a major ubiquitous influence on cellular signal transductions and fate/function of proteins, including the activity and stability of enzymes and transcription factors, functions of receptors and ion channels, protein phosphorylation, ubiquitylation and degradation, cellular redox equilibrium, and apoptosis.^[^ ^87^ ^]^ Third, NO modified prosthetic metals in proteins (e.g., heme iron) and iron‐related gene expression to maintain iron homeostasis and trigger regulatory effects (e.g., crosstalk with CO). Conversely, heme proteins (e.g., sGC, cytochromes, NO‐transporters and sensors, heme‐activated K^+^ channels, etc.), allosterically activated by heme coordination changes post‐NO binding, carried out a variety of vital functions (Table 2; Section S3: Supporting Information).^[^ ^85^, ^88^ ^]^

The NO/sGC/cGMP axis may promote epidermal stem cell proliferation in wound healing and angiogenesis. It operated through NO‐regulated FOXG1 expression via cGMP pathway and FOXG1‐heightened c‐Myc promoter activity, which could be enhanced by H_2_S co‐signaling to inhibit PDE5A (i.e., ↑cGMP).^[^ ^83^ ^]^ For neuroprotection, it was reported that nitrite, an ischemic reservoir of NO and a potent *S*‐nitrosating agent, ameliorated brain injury after asphyxial cardiac arrest in rats by *S*‐nitrosylation of brain mitochondria to reduce reperfusion ROS and maintain ATP generation. Such an effect was NO dependent but not involving the cGC/cGMP signaling pathway.^[^ ^82^ ^]^ In addition, when rat hippocampal neurons were stressed by sublethal OGD, NO, derived from donor and NOS activation, provided partial neuroprotection by indirect stimulation of ATP‐sensitive K^+^ channels via turning on the Ras/MAPK pathway.^[^ ^89^ ^]^ In a murine MCAO (for 1 h) and reperfusion (for 47 h) model, inhalation of NO (10 – 60 ppm × 8–24 h; starting immediately after injury) significantly reduced group average infarct size, likely through enhanced blood flow during reperfusion and anti‐inflammation (Table 2; Section S3: Supporting Information; Figure 4; Figure S1: Supporting Information).^[^ ^90^ ^]^ Conversely, quick interaction between superoxide anions and NO can produce peroxynitrite (ONOO^–^) that adversely causes oxidative damage. The reaction may also exhaust normal NO bioactivity.^[^ ^91^, ^92^ ^]^ Clearly, a judiciously designed treatment to properly control NO level and application time is important for mitigating these potential side effects.^[^ ^91^, ^92^, ^93^ ^]^

#### 3.1.4 Hydrogen (H_2_)

Hydrogen (in Greek, hydro: water; genes: forming), the most abundant element, composes ≈90% of the visible universe. Molecular hydrogen gas (H_2_: the primary existence form of hydrogen) was likely first experimentally produced by Robert Boyle in 1671.^[^ ^94^ ^]^ Henry Cavendish recognized hydrogen (H) as a distinct element a century later.^[^ ^95^ ^]^ The pilot test of treating tuberculosis with H_2_ was led by Thomas Beddoes around 1793.^[^ ^18^ ^]^ Yet, H_2_ production within the human body was not confirmed until 1969.^[^ ^96^ ^]^ In 1975, Dole et al. demonstrated that treatment with a hyperbaric (8 ATA) H_2_ mixture (97.5% H_2_ and 2.5% O_2_ × 2 weeks) caused regression of squamous cell carcinoma in mice, mainly through catalyzing free radical decay.^[^ ^96^ ^]^

Later, administration of H_2_‐rich water or H_2_ gas was shown to reduce urinary secretion of 8‐hydroxydeoxyguanosine and hepatic formation of peroxidized lipids after chemically induced oxidative stress in rats, and to mitigate brain tissue loss in a rat IRI model by neutralizing hydroxyl radical.^[^ ^97^ ^]^ Being able to diffuse across cell membranes and quench deleterious free radicals (e.g., ONOO^–^, OH^•^, etc.) without interruption of physiological oxidation, H_2_‐based treatments hold excellent potential for tissue protection against toxicological pathology, diabetes, neurotrauma, and IRI of the brain, heart, intestine, kidney, liver, and lungs (Table 2; Section S3: Supporting Information).^[^ ^98^, ^99^ ^]^

The tissue protective effects of H_2_ primarily resulted from suppression of cell apoptosis, which involved attenuation of oxidative stress (see above), down‐regulation of Bax expression and caspase 3 activation, and upregulation of Bcl‐2, SIRT1 and HO‐1.^[^ ^100^ ^]^ Administering H_2_‐rich saline in rodent perinatal cerebral IRI (H_2_ inhalation also used) and cardiac arrest models reduced pro‐inflammatory cytokines (e.g., TNF*α*, IL‐1*β*, and TGF‐*β*1), microRNA‐21/‐210 (members of so called hypoxamirs), HMGB1, caspase 3 activation, NF‐*κ*B response, and microglia toxicity; the treatment increased activity of antioxidant enzymes (superoxide dismutase/SOD and catalase) and the number of regulatory T (Treg) cells to conjugatively protect neural tissue.^[^ ^99^ ^]^ Treatment with H_2_ saline (H_2_ dissolved under 0.6 MPa for 2 h; 5 mL kg^−1^, q.d. x 3, starting 24 h post injury) and H_2_ inhalation (3% H_2_+33% O_2_+64% N_2_/4 L min^−1^, beginning 10 min before IRI for 140 min) significantly reduced brain edema and infarct size by reducing ER stress (via ↓GRP78 and C/EBP homologous protein), in HIBI neonatal mice and mitigated neuronal loss and motor deficits by decreasing extracellular glutamate level (via ↓GluT1) in rats with spinal cord IRI, respectively (Table 2; Sections S3 and S4: Supporting Information).^[^ ^101^ ^]^

In terms of negative results (Table S1: Supporting Information), in moderate and severe Rice‐Vannucci models (i.e., P10 rat pups undergoing unilateral ligation of the common carotid artery followed by exposure to 8% O_2_ hypoxic air for 120 and 150 min, respectively), pre‐, intra‐, and post‐injury inhalation of 2.9% H_2_ all failed to significantly reduce infarct volume and brain concentration of MDA, an end‐product of lipid peroxidation. In addition, H_2_ pretreatment was associated with increased infarct area in neonatal hypoxic brains and cerebral MDA level in non‐ischemic neonates.^[^ ^102^ ^]^ Compared to the beneficial outcomes observed in the neonatal mice with HIBI after H_2_ saline administration and an earlier work where 2% H_2_ inhalation showed marked neuroprotection in a mild Rice‐Vannucci model (90 min hypoxia),^[^ ^103^ ^]^ the data suggested that the impact of H_2_ on oxidative damages is defined by the balance between selective antioxidant effect and circumstantial prooxidant influence of H_2_, and the local and global free radical dynamics of superoxide anion (O_2_^•‐^), nitric oxide (^•^NO), and hydroxyl (^•^OH) radicals.^[^ ^104^ ^]^ Accordingly, the pharmacodynamics of these oxidants and H_2_ could be modified by pathophysiological aspects of a particular injury or disease model, lesion severity, injury time course, and the age of the subject.^[^ ^104^ ^]^

#### 3.1.5 Oxygen (O_2_)

Oxygen as a nomenclature is in fact a double misnomer: Lavoisier proposed that it was an essential element for all acids (the Greek root “oxys” means “sharp,” referring to the taste of acids, and “‐genes” translates to “begetter”); oxygen refers to elemental oxygen (with the 8^th^ atomic number and a symbol of “O”), not the “oxygen” as a clinical term (i.e., dioxygen: O_2_, the most stable form of O).^[^ ^15^, ^16^ ^]^ As described above, O_2_ is a traditional medical gas valued for its essential role in sustaining aerobes on the earth. This signature has made the application profile of O_2_ in hospitals different from all other medical gases. For this review, hyperbaric O_2_ (HBO) therapy was focused on for its prevalent use in promoting recovery for patients with ischemic and/or hypoxic lesions in various organs, drug resistant microbial infections, or/and full thickness wounds. HBO increases O_2_ concentration in the blood and interstitial space to counteract hypoxic lesions, maintains cellular reservoirs of O_2_, and attenuates adverse consequences (e.g., cytotoxic and vasogenic edema, inflammation, metabolic perturbation, and apoptotic cell death), as well as exerts direct bactericidal effects on anaerobic pathogens to provide tissue protection (Table 2; Section S3: Supporting Information).^[^ ^105^ ^]^

HBO‐elevated interstitial pO_2_, by definition, is an oxidative stimulus that increases the production of ROS and RNS and initiates cell survival signaling and antioxidant reactions. In fact, HBO has been shown to promote tissue remodeling and functional restoration through interaction with other gaseous messengers to modulate multiple signaling pathways. As examples, the level of SDF‐1, a pivotal factor in mobilizing various progenitors,^[^ ^45^ ^]^ was elevated by HBO treatment via improving stability and activity of HIF‐1*α*, which reciprocally increased SDF‐1 and dermal fibroblast proliferation.^[^ ^106^ ^]^ HBO therapy also stimulated the IL‐6/STAT3 pathway in an early phase of a muscle injury model.^[^ ^107^ ^]^

HBO treatment broke DNA strands and caused an initial inhibition of HO‐1 synthesis that was later increased (i.e., 12–18 h after HBO exposure) in cultured rabbit lens epithelial cells.^[^ ^108^ ^]^ Since administering hemin also augmented HO‐1 production, the HBO‐induced HO‐1 elevation might have resulted from an immediate inhibition of protein synthesis, leading to an accumulation of heme, rather than a direct antagonism to oxidative insult.^[^ ^108^ ^]^ Data from a clinical study supported this conclusion: exposure to HBO (i.e., 100% O_2_ at 2.5 ATA for 20 min x 3) produced DNA damage in lymphocytes of the participants. Yet, induction of DNA breakage only occurred after the first HBO treatment but not in subsequent sessions. There were discernibly elevated levels of HO‐1 in lymphocytes 24 h after HBO treatment. In contrast, superoxide dismutase, catalase, DNA repair enzyme apurinic endonuclease, and DNA polymerase beta were not increased. Importantly, blood lymphocytes collected 24 h after HBO exposure were significantly protected against DNA destruction caused by H_2_O_2_, but not by gamma‐irradiation, suggesting that the survival adaptation was enabled by the HBO‐induced antioxidant defense.^[^ ^109^ ^]^

Pretreatment with HBO significantly reduced infarct volume and neurological deficits in a rat model of MCAO/IRI by suppressing mitochondrial pathway of apoptosis.^[^ ^110^ ^]^ HBO preconditioning also markedly protected cultured rat spinal cord neurons from oxidative and OGD insults by upregulating ROS, NO, and HO‐1 and activation of MEK1/2, ERK1/2, p38 MAPK, CREB, Bach1 and Nrf2 pathways. Among them, the ROS/p38 MAPK/Nrf2 pathway functioned to augment HO‐1 expression; MEK1/2/Bach1 signaling, in contrast, negatively regulated this process.^[^ ^110^ ^]^ HBO also increased HO‐1 in a myocardium IRI model through activating the PI3K/Akt/Nrf2‐dependent system against oxidants.^[^ ^111^ ^]^ Thus, interactions between O_2_, CO, and NO intracellular signaling pathways may collectively decide cell survival or death, and HO‐1, the primary producer of endogenous CO, may serve as a mediator between these pathways. (Figure 4; Figure S1: Supporting Information; Table 2; Sections S3 and S4: Supporting Information).

What has not been systematically studied is how to properly control the HBO‐triggered ROS/RNS production to avoid excessive oxidative lesion and interference to physiological second messengers. Because O_2_ therapy can produce severe adverse effects if not executed properly, judicious treatment design, patient monitoring, and dosage control are crucial. To do so, it has been recommended that mitochondrial health should be monitored as a primary biomarker to preserve HBO's efficacy and prevent toxicity.^[^ ^112^ ^]^

In a rat cerebral IRI model, HBO therapy given within 6 h after IRI onset significantly reduced brain infarction. However, administering HBO ≥12 h p.i. exacerbated tissue loss.^[^ ^113^ ^]^ Whereas HBO was ineffective in filament‐induced permanent MCAO in mice, it was efficacious in sparing parenchyma after filament‐induced transient MCAO.^[^ ^114^ ^]^ Therefore, treatment timing, lesion severity, and injury time course appeared to be factors in determining the effect of HBO on neural ischemia.

For SCI, a recent paper described that HBO therapy (1 h of 100% O_2_ at 3 ATA/d × 10) improved diaphragm‐specific force production and muscle fiber integrity in rats with lateral C3/4 contusion. The effect was mainly derived from HBO‐mediated mitochondrial protection and ROS modulation.^[^ ^115^ ^]^ In rodent TBI models, HBO protected neural cells by increasing expression of IL‐10, Bcl‐2, and Bcl‐xL.^[^ ^116^ ^]^ It is noteworthy that a systematic review of clinical data (40 years through 2017) revealed that when HBO was given ≤30 d of an acute severe TBI, it enhanced clinical recovery signs with no major toxicity.^[^ ^117^ ^]^ For moderate‐to‐severe TBI, the majority of results favored HBO treatment compared to “standard care.” While limited analysis of placebo effect and tissue preservation were conducted on mild TBI (mTBI) with no statistically significant differences being reported between HBO and sham arms in all trials, Walker et al. found that for military service members with post‐mTBI sleep‐wake disturbances, HBO relative to sham significantly improved certain aspects of sleep quality.^[^ ^117^ ^]^ For people suffering from chronic neuro‐cognitive impairment from concussion TBI, HBO therapy enhanced angiogenesis to benefit blood perfusion in the damaged brain area and general cognitive function.^[^ ^117^ ^]^

#### 3.1.6 Ozone (O_3_)

The odor of ozone (O_3_) produced by electrical discharge through ambient air was noticed in 1785 by Martin van Marum.^[^ ^118^ ^]^ Fifty‐four years later (1839), Christian Friedrich Schönbein sensed the same peculiar odor from the oxygen generated during electrolysis of acidulated water. He named it ozone (from the Greek verb “ozein”: to smell), as a discrete gas.^[^ ^119^ ^]^ O_3_ is dynamically unstable due to the presence of mesomeric states. Despite its hazardous properties, O_3_ has been studied for therapeutic purposes since the 1890s. For example, Dr. Henry Norris, in 1892, prescribed O_3_ dissolved in water (termed “Aquozone”) to treat tuberculosis.^[^ ^120^ ^]^ Considering the known serious adverse effects,^[^ ^121^ ^]^ the U.S. FDA banned O_3_ use “in any medical condition for which there is no proof of safety and effectiveness" (see Code of Federal Regulations Title 21 Sec. 801.415 in FDA Website) in April 2016.

Nonetheless, there have been some continued research endeavors concentrating on the possible antioxidant and immune modulation potencies of low dose O_3_‐triggered eustress. For instance, after incubation of endothelial cells with increasing doses of ozonated serum (20, 40, and 80 µg O_3_/mL of serum), a eustress‐dependent activation of Nrf2 was observed, followed by induction of HO‐1 and NQO1 (an antioxidant enzyme) to evoke anti‐inflammation responses.^[^ ^122^ ^]^ O_3_ pre‐exposure (2 ppm x 3 h) enhanced the innate immune response in the lungs by decreasing MIP‐1*α* and TNF*α* and increasing the expression of CXCL‐1, IL‐6, and TLRs in macrophages.^[^ ^123^ ^]^ Furthermore, a wound healing study reported that administration of 400 µL O_3_ oil topically (b.i.d. × 12) activated the PI3K/Akt/mTOR pathway to promote the epithelial‐mesenchymal transition (Figure 4; Figure S1: Supporting Information; Table 2; Section S3: Supporting Information).^[^ ^124^ ^]^ Contrariwise, the activation of NF‐*κ*B‐light‐chain‐enhancers in B cells is a major mechanism for toxic levels of atmospheric O_3_ to induce harmful inflammation. This promotes transcription of pro‐inflammatory cytokines and, in turn, sets certain antioxidative events in motion.^[^ ^122^ ^]^

### 3.2 Noble Gases

The nomenclature of noble gases arose from the fact that these elements are normally unreactive (i.e., inert) toward other elements or compounds. Despite said inertness and a relatively limited understanding of their mechanism of action, noble gases have been utilized for various purposes in hospitals and extensively investigated for applications in anesthesia, IRI amelioration, tissue preservation, and neural repair.^[^ ^125^ ^]^

#### 3.2.1 Xenon (Xe)

William Ramsay and Morris Travers first isolated xenon (Xe), the fifth of the noble elements in 1898.^[^ ^126^ ^]^ By 1946, the anesthetic effect of normobaric Xe was observed in mice.^[^ ^127^ ^]^ The general anesthetic effect of Xe inhalation in humans was reported by Cullen and Gross in 1951,^[^ ^128^ ^]^ which was subsequently adopted as a clinical anesthetic gas.^[^ ^129^ ^]^ Around the early 2010s, experimental and clinical research began to reveal the tissue protective effect of Xe on the nervous and cardiovascular systems, especially under ischemic and hypoxic circumstances.^[^ ^130^, ^131^, ^132^ ^]^

Despite being a noble element, Xe can interact with amino acid residues and modulate the functional properties of surrounding proteins due to its large atomic radius and relatively high polarizability.^[^ ^126^ ^]^ As an anesthetic and cytoprotectant, Xe interacts with the glycine binding site to block the NMDA subtype of glutamate receptors in excitatory neurotransmission, suggesting that Xe may hold high potential as a neurotherapeutic (see Sections 4.2., 4.3.3. and 4.3.4. for details).^[^ ^130^, ^133^, ^134^ ^]^ Xe has been shown to modulate the biological function of background (i.e., two‐pore domain K^+^ channels that leak K^+^ currents at all potentials) and ATP‐sensitive K^+^ channels, and multiple signaling transductions, such as PI3K/Akt, MAPK, mTOR/HIF‐1*α*, and mitochondrial apoptosis pathways.^[^ ^131^, ^135^, ^136^, ^137^, ^138^, ^139^ ^]^ Specifically, in a mouse model of renal IRI,^[^ ^138^ ^]^ Xe pretreatment provided renoprotection via mTOR pathway‐mediated HIF‐1*α* upregulation (Figure 4; Figure S1: Supporting Information; Table 2; Section S3: Supporting Information).

#### 3.2.2 Argon (Ar)

Henry Cavendish, in 1785, found a small amount of an unknown, unreacted gas remaining after he removed O_2_ and N_2_ from the air.^[^ ^140^ ^]^ More than a century later (1894), Williams Ramsay and John William Strutt separated Argon (Ar; from Greek *αργ*ό*ν*: slack), the third noble element (atomic number 18), from liquified atmospheric air and recognized that it was responsible for the residue noticed by Cavendish. Ar is the most abundant noble gas in Earth's atmosphere.^[^ ^126^ ^]^

Clinically, liquid Ar‐based cryotherapy (boiling point of Ar at 1ATM: −303°F/−186°C) has been used to kill cancer cells.^[^ ^141^ ^]^ Ar lasers have been applied in surgeries for tissue welding (e.g., sealing arteries), ablating tumors, correcting vision defects, and treating various eye conditions including diabetic eye complications and glaucoma.^[^ ^142^ ^]^ Because of its low cost, excellent safety record, and easy application, Ar has been actively studied for therapeutic potentials in several systems, involving cell cultures under LPS, hypoxic or OGD exposure, and animal models of IRI, MCAO, subarachnoid hemorrhage, and multiorgan failure. Overall, post‐insult systemic administration of Ar ameliorated inflammation, reduced apoptosis, enhanced cell proliferation and survival, and improved function in the nervous, cardiac, retinal, renal, and hepatic systems by modulating apoptotic and pro‐survival pathways and reducing blood levels of IL‐6 and HMGB1 (Table 2; Section S3: Supporting Information).^[^ ^143^ ^‐^ ^145^ ^]^

As examples, 50% (vol) Ar exposure to plain microglial cell (line BV‐2) cultures for 30 min significantly increased levels of pERK‐1/2, stimulating cell proliferation and survival. In contrast, administration of U0126, an inhibitor of MEK (an upstream activator of ERK), abolished Ar‐induced ERK‐1/2 phosphorylation, indicating that Ar activates microglial ERK‐1/2 via turning on the upstream kinase MEK. However, Ar did not significantly affect 50 ng mL^−1^ LPS‐triggered ERK‐1/2 activation and inflammatory cytokine induction in cultured microglia. Since the study did not examine any longer‐term effect of Ar on microglia, future investigations should verify if Ar would augment beneficial polarization of microglia to enhance tissue repair. In a PND 7 rat model of asphyxia, treatment of 70% (vol) Ar (balanced with O_2_; starting 2 h after insult for 90 min) distinctly improved cell survival, brain structural integrity, and neurological function on PND 40 in both moderate and severe hypoxia‐ischemia groups, compared to those treated with 70% N_2_/30% O_2_. Ar inhalation increased the expression of Bcl‐2 to hinder apoptosis in the non‐directly injured hemisphere.^[^ ^146^ ^]^

For narcotic impact, Ar may directly interact with GABA_A_ receptors at high pressure, because pretreatment with the competitive GABA_A_ receptor antagonist gabazine (0.2 nmol) but not the GABA_B_ receptor antagonist 2‐hydroxysaclofen (10 nmol) significantly increased the argon threshold pressure to induce loss‐of‐righting‐reflex (*p* < 0.005) in rats.^[^ ^147^ ^]^ This mechanism can also contribute to neural protection and neuroplasticity in certain neurological abnormalities (Figure 4; Figure S1: Supporting Information).^[^ ^147^ ^]^ Being physically less polarized than Xe, Ar hardly modulates NMDA receptors and ATP‐sensitive K^+^ channels under normobaric pressure. However, treatment with 75% (vol) Ar for 2 h reduced the expression and density of TLR2 and TLR4 (transmembrane mediator of major immune responses) in human neuroblastoma cells. The effect was associated with suppression of the TLR2 and TLR4‐induced downstream STAT3/NF‐*κ*B activation in combination with activation of the ERK‐1/2/Akt pathway.^[^ ^145^, ^148^ ^]^ Furthermore, Ar inhalation significantly ameliorated apoptosis of animal retina ganglion cells after IRI in vivo via attenuating TLR2 and 4/STAT3/NF‐*κ*B‐induced retinal IL‐8 expression, and preservation of mitochondrial membrane potential to reduce ROS generation (Table 2; Section S3: Supporting Information; Figure 4; Figure S1: Supporting Information).^[^ ^148^ ^]^

For the effect of Ar, some data discrepancies persisted (Table S1: Supporting Information). When analyzed 24 h after the insult, Ar precondition (i.e., 75% Ar/20% O_2_/5% CO_2_ (vol) x 3 h) neither exhibited protection nor modified survival gene expression in human tubular kidney (HK2) cells after 3 h of OGD. In the same study, whereas He exposure worsened the OGD injury, Xe treatment improved cell survival through upregulating expression of pAkt, HIF‐1*α* and Bcl‐2.^[^ ^139^ ^]^ Factors interfering with the cell protection potential of Ar might be related to the two long intervals (24 h/each) between Ar exposure and OGD onset, and between the end of OGD and beginning of data collection.

Based on data obtained from an ex vivo rat brain slice model of OGD and a rat MCAO model, investigators recommended that postischemic Ar treatment be administered during but not after ischemia (i.e., given before but not after reperfusion has occurred) to provide cortical neuroprotection and avoid worsening subcortical brain damage.^[^ ^149^ ^]^ One cause for the detrimental effect of Ar recorded in a few studies might involve the concentration and duration of the treatment. It was demonstrated that Ar manifested a concentration‐dependent dual effect on the enzymatic and thrombolytic efficiency of tPA. Precisely, 25% and 75% (vol) of Ar treatment blocked and increased tPA enzymatic and thrombolytic efficacy, respectively. The concentration of Ar used to manage acute ischemic stroke should be judiciously tailored, factoring in the impact of drug administration timing (e.g., given during ischemia).^[^ ^150^ ^]^

#### 3.2.3 Helium (He)

Helium (from Greek: *ηλιος*/romanized: Helios, lit: sun) was originally detected as a bright yellow spectral line in sunlight during a solar eclipse in 1868 by Pierre Janssen and Joseph Norman Lockyer.^[^ ^151^ ^]^ Helium (He; atomic number 2; group 18), the first in the noble gas group in the periodic table and the second most abundant element in the universe, was isolated on Earth by William Ramsay in 1895.^[^ ^152^ ^]^ For its distinct physicochemical characteristics (i.e., inertness, light atomic weight, odorlessness, tastelessness, non‐irritation to the respiratory tract, high thermal conductivity, and markedly low density, solubility, and boiling point), helium has been clinically applied as an adjunct therapeutic and investigated for cytoprotective effects.

Helium has been administered to treat obstructive respiratory tract conditions such as asthma exacerbation, bronchiolitis, COPD, post‐extubation stridor, and ARDS to improve ventilation efficiency.^[^ ^153^ ^]^ Other physicochemical utilizations of helium are lung imaging and pulmonary function measurements, as hyperpolarized He‐3 (^3^H: a light and stable isotope of helium) can generate high quality images of the pulmonary airspaces.^[^ ^154^ ^]^ Unlike Ar and Xe, helium is a non‐immobilizer, due to its incapability to induce anesthesia. Still, helium treatment was found to lessen the heart and brain lesions associated with IRI and hypoxia.^[^ ^155^, ^156^, ^157^ ^]^ For neuronal protection, helium pre‐ and post‐conditioning of rats (i.e., 70% He + 30% O_2_ for 5 min prior to cardiac arrest and for 30 min post spontaneous circulation restoration) significantly decreased neuronal apoptosis in the hippocampal CA‐1 region, compared to controls treated with 70% N_2_/30% O_2_ (Table 2; Sections S3 and S4: Supporting Information).^[^ ^156^ ^]^

The biological effect of helium was as well mediated by multiple signaling cascades, among which modulation of RISK signaling messengers and pro‐survival pathway factors (e.g., caveolins and hexokinase II; see below) played main roles.^[^ ^156^, ^158^, ^159^, ^160^ ^]^ Moreover, helium protected cardiac and neural tissue in vivo via activation of GSK‐3 and/or the p53 pathway in a mPTP‐dependent manner.^[^ ^161^ ^]^ The impact might also have resulted from helium‐triggered increases of Cav‐1 and Cav‐3 and hexokinase II expressions.^[^ ^156^, ^159^, ^160^, ^161^ ^]^ As a special type of lipid raft, caveolae are small omega‐shaped cholesterol and sphingolipid‐enriched invaginations of the plasma‐membrane (Φ: 50–100 nm) that contain caveolin (Cav). They serve as epicenters of cellular signal transduction. Caveolins, the structural proteins of caveolae and hexokinase II that promotes cell survival by facilitating glycolysis, mediate the effect of cardiac and brain protection of helium.^[^ ^156^, ^159^, ^160^, ^161^ ^]^ In a rat cardiac IRI model (i.e., 25 min of ischemia followed by reperfusion of 5, 15, or 30 min), 15 min of post conditioning with 70% (vol) helium (balanced with O_2_) ventilation significantly heightened amounts of Cav‐1 and Cav‐3 in cardiomyocyte membrane of tissues within the area‐at‐risk, and serum Cav‐3 (i.e., ↑circulation effectors of caveolae). The treatment also significantly raised phosphorylation levels of RISK pathway's cytosolic proteins pERK1/2 and pAkt (Figure 4).^[^ ^159^, ^160^ ^]^

The exact mechanism enabling helium to activate RISK and/or GSK‐3 signaling remains to be determined. As described before, 75% helium pretreatment worsened injury and did not modify the expression of pro‐survival proteins, such as Akt, HIF‐1*α*, and Bcl‐2 in human tubular kidney cells post OGD.^[^ ^139^ ^]^ Despite modulating differential expression levels of Cav‐1, Cav‐3, and Hexokinase II in the heart tissue for cytoprotection, the combined helium pre/post‐conditioning in that rat resuscitation model did not improve neurological function.^[^ ^156^ ^]^ Evidently, more investigations with specific designs are required to address these issues (Table S1: Supporting Information).

### 3.3 Summaries of Gas Agents and Dosages, Study Subjects and Cells, and Experimental Models

All research papers (i.e., representative mechanistic studies) enrolled in Table 2 and Tables S2–S4: Supporting Information (*n* = 62; see Section S3: Supporting Information for abbreviations and reference citations) were further assessed regarding the experimental model (in vivo or in vitro), medical gas administration route or methods, experimental subjects or materials utilized (animals or cell cultures), injury or lesion types, and effective/safe or toxic dosage of each medical gas (Table S2: Supporting Information). The investigations administered medical gases in pure gas form (CO, H_2_, NO, O_2_ or hyperbaric O_2_, O_3_, Ar, He, and Xe), gas‐releasing molecules (e.g., CO: CORM‐2, CORM‐3, CORM ALF‐186, and methylene chloride; H_2_S: NaHS, Na_2_S, and NaGYY/sodium GYY4137; NO: PTA‐NO‐NPs/PTA‐NO nanoparticles, SNAP/S‐nitroso‐N‐acetylpenicillamine, spermine NONOate, NOC‐18, and NaNO_2_; Xe: Xe‐ELIP), and/or other forms of gas donors (e.g., H_2_‐rich saline, ozone oil, L‐arginine, a stimulator of NO synthesis).

Study subjects were Sprague‐Dawley rats, Wistar rats, C57BL/6 mice, APP/PS1 (APPswe PS1dE9) transgenic mice, B6.129P2‐Nos3tm1Unc/J (eNOS‐knockout) mice, 3xTg‐AD mice, Rat pups (7 days old), ICR (CD‐1) mice, BALB/c mice, C57B mice, SV129 mice, Yorkshire piglets (infant), and New Zealand rabbits (male) for in vivo investigations. Furthermore, primary cells or cell lines were used for in vitro post‐injury/stress cytoprotection assays. Human cells were primary brain astrocytes, brain microvascular endothelial cells, cardiac myocyte‐like progenitor cells, umbilical cord vein endothelial cells, immortalized embryonic kidney 293 cells, epidermal stem cells, and neuroblastoma cells. Rodent cells included primary astrocytes from cerebral cortices of Sprague‐Dawley rats, primary hepatocytes from male C57BL/6 (B6) mice, primary hippocampal neurons from newborn Sprague‐Dawley rats, primary spinal cord neurons from embryonic Sprague‐Dawley rats, mixed cortical glial‐neuronal co‐cultures from BALB/c mice, and midbrain cultures from embryos of female Wistar rats. Leporidae cells tested were cardiomyocytes of male New Zealand rabbits (Table S2: Supporting Information).

The dosage information of each medical gas summarized in Table S2: Supporting Information consisted of dose, units, administration route/method (i.e., intraarterial/i.a., intravenous/i.v., intra‐peritoneal/i.p., oral administration/p.o., retro‐orbital, intracerebral, inhalation, topical/local application, or into culture media). Regarding potential toxicity, all tissue protective gas dosages were not linked with any discernible toxicities (Table S2: Supporting Information); NO donors (i.e., SNAP and spermine NONOate in combination, and PTA‐NO‐NPs) were the only medical gases that were also investigated under toxic doses in the reports reviewed (i.e., 5000 × 10^−6^ m SNAP/100 × 10^−6^ m NONOate for 48 h post‐injury; and ≥1 mg mL^−1^ PTA‐NO‐NPs in culture media; other details in Table S2: Supporting Information and references in Section S3: Supporting Information). Noticeably, the number of independent studies utilizing the same standardized model (and/or study design) of a particular trauma, disease or pathophysiological condition was extremely low.

### 3.4 Statistical Analysis

The afore‐described interstudy differences in experimental models, outcome measures, drug deliveries, and research designs preempted any validity to performing conventional statistical comparisons (e.g., meta‐analysis) between studies in order to generate biologically meaningful estimates of effect magnitude of medical gases. To strengthen tangibility assessment of study outcomes, we qualitatively and quantitatively evaluated all representative mechanistic studies enrolled in Table 2 (*n* = 62; reference citations in Section S3: Supporting Information) regarding statistical methods utilized for data analysis. Our findings were presented in Table S3: Supporting Information (sample size, power analysis, statistical method including post‐hoc test, significance level, and software) and Table S4: Supporting Information (data pre‐processing and quality analysis).

Data was presented in mean ± standard error of the mean (SEM), mean ± standard deviation (SD), or median. Sample size ranged from 3 to 16 per experimental group, which, however, appeared to be empirically decided since power analysis was only performed in 3/60 studies (i.e., 5%). Specifically, power analysis was conducted in 1/8 (12.5%), 1/10 (10%), and 1/4 (25%) of CO, H_2_, and He studies reviewed in Table 2, respectively, to estimate adequate sample sizes to avoid a type II error and to have sufficient power (e.g., ≥80%) to detect a specific effect in a comparative study. For data pre‐processing, normalization, transformation, and homoscedasticity procedures were performed in about half of the studies listed in Table 2 (i.e., 34/62 = 55%), but the reports on CO (*n* = 8), NO (*n* = 4), and Xe (*n* = 4) did not specify statistical methods used in data normalization (16/34 = 47%; Table S4: Supporting Information). Thus, future investigations should further improve scientific rigor by increasing implementation of statistical power analysis and data pre‐processing in MGT research conducts.

To determine differences between multiple groups of data, one way ANOVA (48/62, 77%), two‐way ANOVA (12/62, 19%), and Kruskal‐Wallis (i.e., one‐way ANOVA on ranks, a non‐parametric method; 9/62, 15%) were used. For differences between two groups of data, Student's *t*‐test (12/62, 19%), two‐tailed unpaired *t*‐test (1/62, 2%), independent *t*‐test (2/62, 3%), Welch's *t‐*test (2/62, 3%), and Mann‐Whitney U/Wilcoxon Rank Sum Test (used when the dependent variable, either ordinal or continuous, was not normally distributed; 8/62, 13%) were applied. Chi‐squared test was used for comparing categorical data (2/62, 3%), and bivariate analysis (1/62, 2%) for assessing any concurrent relation between two variables or attributes.

Post‐hoc analyses included Bonferroni test (13/62, 21%), Tukey's test (12/62, 19%), Newman‐Keuls test (10/62, 16%), Holm‐Sidak test (5/62, 8%), Fisher's LSD (least significant difference) procedure (4/62, 6%), Dunn's test (3/62, 5%), Dunnett's test (2/62, 3%), Kruskal‐Wallis test (2/62, 3%), F‐test (2/62, 3%), Fisher's exact test (1/62, 2%), Games‐Howell test (1/62, 2%), Paired *t*‐test (1/62, 2%), Post‐hoc *t*‐test (1/62, 2%), Student's *t*‐test (1/62, 2%), Scheffé test (1/62, 2%), and Unpaired *t*‐test (1/62, 2%). The data produced from these tests were evaluated for significance based on *p*‐values (*p* < 0.0001–0.05) and *α* values (*α*  = 0.01–0.05; confidence level = 0.99–0.95; Table S3: Supporting Information). Statistical software packages for computation were SPSS (Statistical Package for the Social Sciences; IBM), GraphPad (GraphPad Software), Sigmaplot (SPSS Inc.), Sigmastat (Systat Software Inc.), Statistica (StatSoft), Statflex (ARTECH Co.), and EZAnalyze (Microsoft) (Table S3: Supporting Information).

## 4 Neuroprotective Effect of CO and Xe

In the following sections, the effects, and their associated mechanisms of CO and Xe on experimental neural abnormalities were analyzed with more details about their impacts on TBI and SCI. All retrieved literature on this topic was summarized in Table 3 and Table 4. Leading mechanisms underlying the effect of CO and Xe were detailed in Figure 5. Papers that described negative results (Table S1: Supporting Information), problems, and challenges were too discussed.

### 4.1 CO

Chemically stable and lipophilic, CO, in low concentrations, does not have toxicity or destructive radical and quenching reactions with proteins or amino acids. This attribute may empower CO to function as a neurotherapeutic to reach long‐distance targets that are guarded by the BBB and BSCB. In vitro, CO released from 30 × 10^−6^ m CORM‐2 significantly reduced apoptotic death of hippocampal neurons (from 6–8 d/o Wistar rats) following exposure to DTDP, an oxidant (25 × 10^−6^ m x 5 min). CO inhibited insertion of Kv2.1, a type of delayed rectifier K^+^ channels, into cell membranes to reverse DTDP‐increased K^+^ efferent current density, an early trigger of apoptosis. Administration of PKG inhibitor, NO donor or NOS antagonist revealed that CO's suppression of Kv2.1 membrane incorporation was PKG‐dependent without involving NO.^[^ ^42^ ^]^ Moreover, pretreatment with CO (25 ppm x 90 min) prevented excitotoxicity‐triggered apoptosis and oxidative stress in murine cerebellar granule cells triggered by glutamate (20–40 × 10^−6^ m x 2 h) or tert‐butyl hydroperoxide (an organic peroxide: 6–28 × 10^−6^ m x 2 h). This antiapoptotic benefit of CO required activation of sGC/cGMP signaling, NO synthase, mitochondrial K^+^ channel (mitoK_ATP_), ROS generation, and de novo synthesis of HO‐1.^[^ ^162^ ^]^ The efficacy may be further amplified through CO's modulation of astrocytic HO‐1 to produce multimodal neuroprotection.^[^ ^55^ ^]^

A unique property of CO is its stimulation of neurogenesis,^[^ ^44^, ^163^ ^]^ a process that has been increasingly appreciated for its multifaceted roles in mitigating secondary injuries and augmenting neural recovery.^[^ ^45^, ^92^ ^]^ Applying 25 ppm CO to hNSC cultures (at 0 and 4 d) significantly increased the rate of cells producing MAP2 (a mature neuronal marker) or tyrosine hydroxylase (the rate‐limiting enzyme of catecholamine biosynthesis), when determined 6 d after differentiation in vitro. CO treatment inhibited caspase 3 activation but augmented production of HIF‐1*α* and ROS without changing the number of cells immunopositive for K_i_67, a cell proliferation marker, suggesting that its main neurogenic mechanism was mediated through mitochondrial and ROS alterations.^[^ ^163^ ^]^

In vivo, pre‐inhalation of CO (250 ppm x 1 h) or injection of CORM (ALF‐186; 25 µg intravitreally or 10 mg kg^−1^, i.v.; given after 1 h of insult) in rat IRI models significantly attenuated retinal ganglion cell apoptosis and inflammation by increasing CREB, p38 MAPK phosphorylation, HSF‐1, HSP‐90, the Bcl‐2/Bax ratio, and GAP‐43, whereas reducing NF‐*κ*B, TNF‐*α*, AIF‐1, HSP‐70, p‐ERK1/2 and activated caspase‐3 levels.^[^ ^164^ ^]^ In cerebral ischemia models (e.g., MCAO and perinatal hypoxia‐ischemia), CO administration markedly mitigated the group average infarct size and elevated angiogenic activities, resulting in significantly improved neural behavioral performance. The effects were mediated through activating Nrf2/OH‐1 signaling and HIF‐1*α* independent expression of VEGF, and mitigation of mitochondrial cytochrome *c* release (Figure 4; Figure S1: Supporting Information; Table 3; Section S3: Supporting Information).^[^ ^41^, ^43^, ^165^ ^]^ The multifaceted impacts of CO make it an attractive therapeutic candidate to counteract secondary injury events.^[^ ^166^ ^]^

Of note, prolonged exposure to even low levels of CO can be harmful to human health. In a case report, mild cognitive deficits in memory and concentration were found in a woman, which were associated with a 3‐year exposure to CO (≈180 ppm) in the basement of her house; however, no abnormalities of general intelligence, visuospatial function, and speed and dexterity were detected in the patient, indicating that adults may tolerate lengthy contact with low doses of CO without manifesting more serious clinical signs.^[^ ^167^ ^]^ Delayed (frequently within the first month) neuropsychiatric consequences (e.g., dementia, memory deficit, personality changes, learning difficulties, etc.), by contrast, occur after acute, high dose CO poisoning, especially in individuals who have survived CO‐induced unconsciousness.^[^ ^167^ ^]^

Low doses of CO may negatively affect the developing brain. Inhalation of 25 ppm CO during PNDs 8–22 in rats reduced group average immunoreactivity to neurofilament, myelin basic protein, cytochrome oxidase, NADH‐TR, and calcium ATPase in the organ of Corti as well as neuronal density of the spiral ganglion, compared to normal controls.^[^ ^168^ ^]^ Conversely, CO's anti‐apoptotic effect, if persisting over a protracted time, can impair brain development because neuronal apoptosis normally plays a crucial role in regulating synaptogenesis. In mice (10 d/o), a 3‐h exposure to 5 ppm or 100 ppm CO mitigated cytochrome *c* release, caspase‐3 activation, and apoptosis in neocortex and hippocampus, increasing neuronal numbers. CO exposure consequentially compromised learning, memory, and social behavior in the mice when evaluated 4–5 weeks later. However, the study did not examine the impacts at any later time points (e.g., >10 weeks when they reach sexual maturity).^[^ ^169^ ^]^ Therefore, possible adverse effects of low doses of CO must be clinically assessed as per the exposure time course and the sensitivity of a particular individual (e.g., patient stratification derived from disease/trauma‐specific CO sensitivity phenotype and genotype–phenotype correlation) or developmental stage of a target organ and system.

### 4.2 Xe

The atomic size of Xe is comparatively bigger, rendering its electron clouds more polarizable (see above), to exert weak Van der Waals forces between polar molecules. Thus, Xe is interactive with multiple proteins, likely in therapeutic ways, making it an appealing neurotherapeutic candidate. Xe treatment protected rat embryotic cortical neurons under N_2_‐induced hypoxia in vitro by preventing excessive release of glutamate and regulating Ca^2+^ signaling, as this effect was nullified by preconditioning neurons with BAPTA‐AM, a Ca^2+^‐chelator.^[^ ^170^ ^]^ In another in vitro experiment, PC12 cell‐derived dopaminergic neuron‐like cells were markedly preserved by Xe under hypoxic conditions, showing minimal Ca^2+^‐induced vesicular dopamine discharge.^[^ ^171^ ^]^

The primary neuroprotective mechanism of Xe, based on the following data, was its competitive inhibition of NMDA receptors. In an organotypic murine hippocampal slice model of focal brain trauma, Xe or Ar (i.e., 0.5 ATM of 50% [vol] Xe or Ar given to a sealed incubator chamber containing 1 ATM of 75% N_2_/20% O_2_/5% CO_2_ [total pressure: 1.5 ATM] for 30 min – 24 h) applied ≈5 min after mechanical injury significantly preserved CA1 region, compared to controls treated with N_2_, He, Ne, and Kr. Notably, administration of glycine could reverse the neuroprotective outcome of Xe, but not Ar, suggesting Xe's competitive suppression of the NMDA receptor's glycine‐binding site.^[^ ^130^ ^]^

Incubation of E15.5 Wistar rat ventral midbrain tissue with 75% (vol) Xe/20% O_2_/5% CO_2_ in a normobaric setting significantly lessened dopaminergic neuron death caused by glutamate excitotoxicity staged by _L_‐trans‐2,4‐PDC (100 × 10^−6^ m), a L‐glutamate transporter inhibitor. The neuronal rescue was enabled by the NMDA receptor antagonizing effect of Xe since memantine, an uncompetitive NMDA blocker, mimicked the benefit. Xe treatment also decreased spontaneous neuronal death in standard cell cultures through inducing paracrine neuroprotection of astrocyte‐produced neurotrophic factors. These effects were Xe specific: unlike what was attained from the hippocampal slice assays,^[^ ^130^, ^172^ ^]^ they were not reproducible by Ar in the same setting.^[^ ^134^ ^]^ Through inhibition of overactive NMDA receptors to interrupt cytotoxic Ca^2+^ influx, Xe was able to protect neurons in models of excitotoxicity, OGD, stroke, and hypoxia.^[^ ^135^ ^]^

In vivo, preconditioning (120 min) of PND 7 rats with 75% (vol) Xe/25% O_2_ decreased group average infarct size resulting from asphyxia (i.e., right common carotid artery ligation under anesthesia 4 h after preconditioning), followed with hypoxia (8% O_2_/37°C/3.5 h), and improved long‐term (30 d after ischemia) rotarod performance. The effect of Xe inhalation was associated with enhanced CREB signaling.^[^ ^137^ ^]^ A similar regimen when combined with hypothermia produced synergistic neuroprotection based on morphological criteria, hemispheric weight, and rotarod performance assessed up to 30 d.p.i.^[^ ^173^ ^]^ In a murine model of focal stroke, Xe pre‐conditioning (i.e., 70% Xe or 70% N_2_ [control] with 30% O_2_ for 2 h) significantly lowered group average cerebral infarct volume in both male and female mice that had 1 h of MCAO at 24 h after Xe pre‐exposure and were allowed to recover for 24 h. Xe treatment quantitatively upregulated HIF‐1*α* and pAkt.^[^ ^173^ ^]^

Lastly, systemic injection of Xe‐containing echogenic liposomes (Xe‐ELIP: 7 or 14 mg kg^−1^, i.a.) done 2–5 h post a transient MCAO (2 h) in adult rats significantly lowered group mean infarct volume, relative to control treatment, by activating the MAPK and Akt pathways and augmenting BDNF expression (Figure 5; Table 4; Sections S3 and S4: Supporting Information).^[^ ^174^ ^]^ To determine potential toxicity of Xe in the dosage range for general anesthesia, fluoro‐Jade and fluorescent dye exclusion assays were performed 24 h after exposing organotypic hippocampal slices from 7 d/o rats to Xe (at 0.75, 1, or 2 minimum alveolar concentration [MAC] partial pressures: 60% Xe at 1.2, 2.67, and 3.67 ATM) for 6 h. Exposure to 1 or 2 MAC Xe triggered marked neuronal death (>40%) in CA1, CA3, and dentate regions.^[^ ^175^ ^]^ Replicative investigations will be required to validate this finding before mechanistic assays can be done to reach a conclusion.

### 4.3 CO or Xe Treatment for Experimental SCI and TBI

#### 4.3.1 The Effect of CO on SCI

The rationale of developing CO‐based interventions was partly built upon data collected in the early 2000s. It was exhibited that there were injury site‐specific inductions of HO‐1 and HO‐2 after a 9–10th thoracic spinal cord compression (clip: 15 g x 30 min) in adult male and female mice.^[^ ^176^ ^]^ HO‐1 and HO‐2 mRNA levels were significantly increased in spinal cord segments caudal (at 4 and 16 h p.i.) and rostral (more prominently at 16 h p.i.) to the injury epicenter. Western blot results showed similar upregulations of HO‐1 and HO‐2 proteins after SCI. Furthermore, IHC assays determined that HO‐1 and HO‐2 were mainly expressed by the ventral horn motor neurons, with HO‐2 being also augmented in neurons of the Clarke's Nucleus (located in Rexed lamina VII and involved in unconscious proprioception), oligodendrocytes, and ependymal cells. HO enzymatic activity measurements corroborated the protein expression data. The investigators hypothesized that HO‐1 might counteract cell intrinsic suicide programs, and HO‐2 might suppress RNS‐ignited inflammatory response.^[^ ^176^ ^]^

The effect of CO on neural recovery was recently examined in a rat T9 clip compression model (30 g x 1 min).^[^ ^177^ ^]^ CORM‐3 (8 mg kg^−1^ d^−1^), given immediately after injury until the end of the study, resulted in inhibition of inflammasome signaling and pyroptosis, partly through modulating clip injury‐activated IRE1, an important proximal ER stress sensor (note: prolonged IRE1 activation causes apoptosis^[^ ^178^ ^]^) and a central mediator of the unfolded protein response (UPR) for cell fate adaptation, and XBP1, a nuclear transcription factor that, downstream of IRE1*α*, is a key component of the UPR.^[^ ^177^, ^178^, ^179^, ^180^ ^]^ However, the presented post‐euthanasia (≥28 d.p.i.) histopathology showed near epicenter lesion volume only within the dorsal funiculus of the spinal cord. With sparing of the propriospinal, raphespinal, reticulospinal, rubrospinal and vestibulospinal tracts, and ventral and dorsolateral corticospinal tracts, it is unclear why the control T9‐compression rats manifested severe locomotor deficits (i.e., no body weight bearing hindlimb stepping).^[^ ^177^, ^179^ ^]^

#### 4.3.2 The Effect of CO on TBI

It was reported in 2006 that heightened concentrations of HO‐1 was detected in the cerebral spinal fluid of infants and children (mean: 2.75 ± 0.63 ng mL^−1^; *n* = 48) post TBI, compared to a control group (<0.078 ng mL^−1^ – not detectable; *n* = 7; *p* < 0.001). Notably, the amount of HO‐1 increase was positively correlated with the injury severity and unfavorable neurological outcome levels (*p* < 0.05 for both).^[^ ^181^ ^]^ Infants appeared to have higher HO‐1 increases than older children after TBI, suggesting that HO‐1, a cell protectant (see Section 3.1.1.), may be more inducible in younger brains.^[^ ^181^ ^]^

By 2016, it was reported that treatment with CORM‐3 (4 mg kg^−1^, via retro‐orbital injection at 1 h p.i.) and CO inhalation (250 ppm; 1 h after TBI for 1 h) had a dual effect on pericytes and neurogenesis, resulting in neurological behavioral benefits in C57Bl/6 mice (male; 3 months old) after TBI (controlled cortical impact [velocity: 6 m s^−1^; depth: 0.6 mm/150 ms] to the right parieto‐temporal cortex).^[^ ^44^ ^]^ CO significantly reduced pericyte death and maintained vascular integrity through impeding HIF‐1*α* and Caspase‐3 activation. Further, post‐CO exposure pericytes exhibited signs of signaling crosstalk with NSCs via nNOS phosphorylation, which increased proliferating NSC numbers and neurological recovery. In contrast, administration of free radical scavenger (i.e., PBN; 40 mg kg^−1^, i.p.) alone did not result in functional improvement, despite mitigation of apoptosis, suggesting the pro‐neurogenic effect of CO might be crucial.

This rationale was supported by data from an in vitro assay in which treating NSCs with condition medium collected from human pericyte culture treated with CORM‐3 (200 × 10^−6^ m) increased neuronal differentiation rate. Thus, CO‐elevated NSC proliferation and subsequent NSC‐derived neurons might have aided post‐TBI recovery.^[^ ^44^ ^]^ To this end, it has been uncovered that proliferating NSCs and NPCs possess functional multipotency that enables them to secrete trophic factors (e.g., VEGF, BDNF, GDNF, etc.), exosomes, and pro‐recovery cytokines (e.g., IL‐10, LIF, etc.), as well as form gap junctions to promote homeostasis and functional restoration (Figure 4; Table 3; Section S3: Supporting Information).^[^ ^182^, ^183^ ^]^

#### 4.3.3 The Effect of Xe on Spinal Cord Abnormalities

For the defined timeframe, we did not find any reports that described the neurotherapeutic effects and mechanisms of Xe treatment against traumatic SCI. A study published in 2014, nevertheless, explored the cytoprotective impact of Xe on spinal cord IRI (i.e., 25 min balloon occlusion of the descending thoracic aorta at 3–4 mm caudal to the left subclavian artery). Briefly, Xe inhalation (50% Xe/50% O_2_ x 3 h) administered at the initiation of reperfusion or 1 and 2 h after reperfusion, significantly reduced motor neuron death and hindlimb locomotor deficit in adult rats 4, 24, and 48 h after IRI, compared to the control treatment given immediately after ischemia (50% N_2_/50% O_2_ x 3 h; *p* < 0.05; *n* = 10/each group).^[^ ^184^ ^]^

The same team later showed that rats in the IRI + Xe group had significantly better locomotion scores, more spared motor neurons, fewer apoptotic neurons, and higher levels of pAkt and pERK at 6, 12, 24, and 48 h after reperfusion, relative to the control group. The data, verified via applying specific antagonists of PI3K‐Akt and ERK messengers, indicated that Xe treatment strengthened neurological recovery by activating these signaling pathways (Figure 4; Figure S1: Supporting Information; Table 4; Section S3: Supporting Information).^[^ ^136^ ^]^

#### 4.3.4 The Effect of Xe on TBI

In a controlled cortical impact model of TBI, adult male C57BL/6N mice (2.5 m/o; 24 ± 3 (SEM) g; *n* = 21 for behavioral test or 9 for contusion volume) treated with 75% Xe and 25% O_2_ for 3 h starting 15 min p.i., exhibited significantly improved mean neurological score and contusion volume determined at 24 h after trauma, compared to the control group receiving 75% N_2_/25% O_2_ (*n* = 22). Discernible locomotor enhancement was too observed in Xe‐treated mice 1 month after TBI. The benefits of Xe treatment were associated with lessened secondary brain tissue lesion.^[^ ^185^ ^]^

In a follow‐up study, the same regimen of Xe inhalation for adult male C57BL/6N mice (2.5 m/o; 23.924 ± 0.1 g) with TBI significantly reduced acute neurological dysfunction and lesion volume (*p* < 0.05 and 0.01, respectively; *n* = 9/each group) at 24 h post TBI. Moreover, at 20 months after TBI, Xe treatment blocked development of late‐onset memory deficits (*p* < 0.05; *n* = 7, sham; *n* = 9, control; and *n* = 13, Xe treatment), which was correlated with mitigated chronic white matter degeneration in the contralateral corpus callosum and neuronal death in the contralateral hippocampal CA1/dentate gyrus regions (*p* < 0.05; *n* = 7 or 5, sham; *n* = 8 or 7, control; and *n* = 11 or 10, Xe treatment, respectively). The long‐term neuroprotective effects were confirmed by a significant reduction in levels of reactive gliosis and microglial numbers in the brain areas influencing the behavioral tests.^[^ ^186^ ^]^ Future investigations should verify whether such therapeutic effects of Xe and CO (see above) on TBI are replicable in adult female mice.

## 5 Summary, Conclusions, Challenges, and Future Directions

In 223 qualified publications, 212 papers showed positive results (212/223 = 95%; citations #29 and #191 reported benefits of 2 and 3 gases, respectively). There were 11 reports with negative findings (11/223 = 5%), and another 3 papers (citations #192, 199 and 211) described both negative and positive data of different gases simultaneously studied (total: 11+3 = 14; see Section S2: Supporting Information and Figure 3). About 83% of all enrolled reports described studies on biological gases, among which H_2_S (25%: 57 papers) and CO (22%: 50 papers) were the most frequently investigated, compared to 4–12% of other 4 biological gases (Figure 3). The remaining 17% of the reports were about noble gases (7%/Xe, 6%/Ar, and 4%/He; Figure 3).

There are four major points to be drawn from this systematic review. First, the majority of studies, which met the inclusion criteria, demonstrated tissue protective effects of medical gases on clinically relevant disorders, modeled in vitro or in vivo (i.e., supporting the central hypothesis; Table 2). Second, the beneficial effects of MGT were mediated through multimodal mechanisms, to concurrently antagonize, activate, and/or modulate key signaling pathways of stress, oxidation, inflammation, cell death and survival, stem cell fate, metabolic homeostasis, and function. These common pathways can also be affected by other pathophysiological conditions or diseases (see examples of therapeutic impacts of medical gases on non‐neurotrauma models in Table 2). Third, critical mechanistic targets need to be validated using leading molecular and genetic technologies, and comparably designed studies are required before meta‐analysis can be performed. Fourth, the infiltrating, broad spectrum, anti‐disequilibrium, homeostatic efficacies exerted by MGT, if systematically reconfirmed, may represent a readily deliverable pharmaceutical approach to treating a whole array of acute and chronic clinical conditions.

In terms of challenges, all in vivo investigations reviewed were conducted in small animal models, without fully factoring in sex and age as biological variables.^[^ ^187^ ^]^ Except for very few studies (e.g., Kim et al., 2014,^[^ ^61^ ^]^) only conventional methods (e.g., PCR, Western blot, etc.) were used to verify putative target molecules. In addition, no reports on combinatorial MGT were qualified for enrollment. Therefore, it is urgent to exploit cutting‐edge technologies of functional genomics, proteomics, next generation gene sequencing, and gene knock‐in, silencing, knockdown, knockout or editing to authenticate key mechanisms of MGT.^[^ ^188^ ^]^ Furthermore, protocols to administer multiple gas molecules and newly emerged ex‐vivo models, especially 3D cell cultures and organ‐modeling organoids derived from disease/condition‐specific hiPSCs, should be utilized to expedite MGT research.^[^ ^189^ ^]^

For pharmacological logistics, direct inhalation has been the most extensively adopted drug delivery method in the field. This approach appears to be promoted by the physiological route of gas intake, lipophilic feature of gas molecules, practicability of general dose adjustment, and historical influence from inhalation therapies (see above). Yet, pragmatic management perspectives can be drastically different between medical gases, particularly regarding rarity, cost, and risk (e.g., Xe, a trace gas is extremely expensive). Furthermore, inhalation as a method has several drawbacks such as high cost of gas storage, difficulties in precisely controlling gas concentration, and possible off‐site adverse effects. Developing pharmaceutical grade pro‐drugs will thereby be a more effective strategy to enable traditional route delivery of medical gases (e.g., CORM and Xe‐ELIP).^[^ ^53^, ^174^, ^190^ ^]^ In spite of a long history concerning various clinical applications of medical gases, only oxygen therapy is currently prescribed in standardized manners for clinical problems of patients. Applying MGT to effectively manage serious diseases and traumas remains an unmet medical demand. In the coming decades, the atmosphere, with its vast capacity, will likely become the newest frontier for the discovery of therapeutics in general, and neurotherapeutics in particular.

## Conflict of Interest

The authors declare no conflict of interest.

## Supporting information

Supporting Information
