# Supplementary material for: Medical Gas Therapy for Tissue, Organ, and CNS Protection: A Systematic Review of Effects, Mechanisms, and Challenges
Source: Adv Sci (Weinh). 2022 Mar 4;9(13):2104136. doi: 10.1002/advs.202104136 (PMC9069381; doi:10.1002/advs.202104136)
Supplement: Supplementary file 1 — Supporting Information [file ADVS-9-2104136-s001.pdf]

ADVANCED SCIENCE
Open Access

Supporting Information

for *Adv. Sci.*, DOI 10.1002/advs.202104136

Medical Gas Therapy for Tissue, Organ, and CNS Protection: A Systematic Review of Effects, Mechanisms, and Challenges

Ross D. Zafonte, Lei Wang, Christian A. Arbelaez, Rachel Dennison and Yang D. Teng**

Supporting Information

Table S1

Summary of Articles Exhibiting Adverse Effects or No Discernible Therapeutic Benefits of Medical Gases

Author/Year	Gas	Model	Findings
Lopez, I. 2003[1]	CO	In vivo: 8-day old rat pups	Mild chronic CO exposure hindered cochlea development.
Cheng, Y. 2012[2]	CO	In vivo: 10-day old CD-1 mouse pups	Three-hour exposure to 5 ppm or 100 ppm CO mitigated developmental apoptosis in neocortex and hippocampus, increasing neuronal numbers to trigger megalencephaly with impaired memory, learning, and socialization.
Cheung, N. S. 2007[3]	H_2S	In vitro: murine primary cortical neurons	H_2S induced neuronal death and apoptosis through activating ionotropic glutamate receptors.
Kim, D. S. 2020[4]	H_2S	In vivo: mouse after acute exposure to H_2S	H_2S caused lesions in the inferior colliculus (IC) and thalamus (TH) via modulation of multiple biological pathways including unfolded protein response, neurotransmitters, oxidative stress, hypoxia, calcium signaling, and inflammatory response in the IC and TH.
Matchett, G. A. 2009[5]	H_2	In vivo: neonatal hypoxia-ischemia rat model	H_2 (2.9%) did not ameliorate neural damage of moderate to severe neonatal hypoxia-ischemia insults.
Takeuchi, S. 2016[6]	H_2	In vivo: intracerebral hemorrhage rat model	H_2 did not attenuate brain water content or improve functional outcome.
Badr, A. E. 2001[7]	O_2	In vivo: MCAO rat model	HBO therapy for ≥12 h significantly exacerbated the cerebral infarct area.
Veltkamp, R. 2006[8]	O_2	In vivo: rat transient and permanent MCAO model	O_2 was not effective in reducing neural lesion after permanent MCAO.
Hjelde, A. 2002[9]	O_2	In vivo: rat permanent MCAO model	HBO did not reduce neutrophil infiltration into the infarct brain tissue and did not mitigate tissue damage in rats after 4h of permanent focal ischemia, relative to control treatment.
David, H. N. 2012[10]	Ag	Ex vivo: OGD injury in brain slices<br>In vivo: MCAO rat model	Post-ischemic argon exposure worsened MCAO-caused subcortical brain damage and did not improve neurologic outcome, compared to control animals.
Harris, K. 2013[11]	He	Ex vivo: mechanical trauma in the organotypic hippocampal brain slices	Helium, neon, and krypton did not result in neuroprotection.
Aehling, C. 2018[12]	He	In vivo: rat cardiac resuscitation model	The combined helium pre/post-conditioning did not benefit neurofunctional outcome.
Rizvi, M. 2010[13]	Ag & He	In vitro: OGD injury in human tubular kidney cells	Neon, argon, and krypton did not protect cells from OGD. Helium worsened cell injury.
Brosnan, H. 2013[14]	Xe	Ex vivo: organotypic hippocampal cultures	Xenon causes neuronal cell death in an in vitro model of the developing rodent brain.

Abbreviations: HBO, hyperbaric oxygen therapy; MCAO, middle cerebral artery occlusion; OGD, oxygen glucose deprivation.

References

[1] I. Lopez, D. Acuna, D. S. Webber, R. A. Korsak, J. Edmond, J Neurosci Res 2003, 74, 666.

[2] Y. Cheng, A. Thomas, F. Mardini, S. L. Bianchi, J. X. Tang, J. Peng, H. Wei, M. F. Eckenhoff, R. G. Eckenhoff, R. J. Levy, PLoS ONE 2012, 7, e32029.

[3] N. S. Cheung, Z. F. Peng, M. J. Chen, P. K. Moore, M. Whiteman, Neuropharmacology 2007, 53, 505.

[4] D. S. Kim, P. Anantharam, P. Padhi, D. R. Thedens, G. Li, E. Gilbreath, W. K. Rumbeiha, Toxicology 2020, 430, 152345.

[5] G. A. Matchett, N. Fathali, Y. Hasegawa, V. Jadhav, R. P. Ostrowski, R. D. Martin, I. R. Dorotta, X. Sun, J. H. Zhang, Brain Res 2009, 1259, 90.

[6] S. Takeuchi, K. Nagatani, N. Otani, K. Wada, K. Mori, Turk Neurosurg 2016, 26, 854.

[7] A. E. Badr, W. Yin, G. Mychaskiw, J. H. Zhang, Am J Physiol Regul Integr Comp Physiol 2001, 280, R766.

[8] R. Veltkamp, L. Sun, O. Herrmann, G. Wolferts, S. Hagmann, D. A. Siebing, H. H. Marti, C. Veltkamp, M. Schwaninger, Brain Res 2006, 1107, 185.

[9] A. Hjelde, M. Hjelstuen, O. Haraldseth, D. Martin, R. Thom, O. Brubakk, Eur J Appl Physiol 2002, 86, 401.

[10] H. N. David, B. Haelewyn, M. Degoulet, D. G. Colomb, Jr., J. J. Risso, J. H. Abraini, PLoS ONE 2012, 7, e30934.

[11] K. Harris, S. P. Armstrong, R. Campos-Pires, L. Kiru, N. P. Franks, R. Dickinson, Anesthesiology 2013, 119, 1137.

[12] C. Aehling, N. C. Weber, C. J. Zuurbier, B. Preckel, R. Galmbacher, K. Stefan, M. W. Hollmann, E. Popp, J. Knapp, Acta Anaesthesiol Scand 2018, 62, 63.

[13] M. Rizvi, N. Jawad, Y. Li, M. P. Vizcaychipi, M. Maze, D. Ma, Exp Biol Med (Maywood) 2010, 235, 886.

[14] H. Brosnan, P. E. Bickler, Anesthesiology 2013, 119, 335.

Table S2

Analysis of Experimental Models and Medical Gas Dosages in Studies Exhibited in Table 2

Gases	Therapeutic Agents	Study Subjects/Materials	Injury/Lesion Types	Safe and/or Effective Dosage	Toxic Dosage
	Biological Gases				
CO	In vitro: CORM-2<br>In vivo: CO Gas, CORM ALF-186, CORM-2, CORM-3, and Methylene Chloride	In vitro: Human Umbilical Cord Vein Endothelial Cells, Primary Human Brain Astrocytes, and Human Brain Microvascular Endothelial Cells<br>In vivo: Male Wistar rats, Male/Female Sprague Dawley rats, and Male C57BL/6 mice	In vitro: Excitotoxicity, oxidative stress, hypoxia, and OGD<br>In vivo: Retinal, kidney or cardiac I/R injury, optical nerve crush, MCAO, cerebral hypoxia-ischemia, neurotrauma, myocardial infarction, heat shock-induced lung injury, acetaminophen-triggered liver lesion, and hepatectomy	**CO gas**: 250 ppm CO gas inhalation for 1 h post-injury [6]<br>**CORM-2**: 5-20 µM RuCO added to culture media for 2-6 h [1a], 100 µM CORM-2 added to culture media for 8 h [2a], 5-100 µM CORM-2 added to culture media for 2-12 h [4], 30 mg/kg i.p. CORM-2 pretreatment for 2 h pre-injury [5b], and 0.1/1.0/10 mg/kg i.v. SMA/CORM-2 injected 24 h pre-BLM injections for 33 d [5c]<br>**CORM-3**: 4 mg/kg or 8 mg/kg dissolved in distilled water, i.v., CORM-3 injected post-injury [1b]<br>4 mg/kg body weight dissolved in saline, i.p. CORM-3 injected 1 h post-injury [6]<br>**CORM ALF-186**: 10 mg/kg body weight i.v. dissolved in water injected 0-3 h post-injury [5a]<br>**Methylene Chloride**: 500 mg/kg p.o. 3 h pre-injury [3]	All medical gas dosages were well tolerated and safe in the experimental models investigated (see *Left Columns)*. For the purposes of the cited studies (**Table 2**), no toxic doses (gases in further higher concentrations) were evaluated
H_2S	In vitro: NaHS, and Diallyl Trisulfide (DATS)<br>In vivo: NaHS, Na_2S, H_2S Donor (NaGYY), and H_2S Donor (PTA-NO-NPs)	In vitro: Primary Astrocytes (from cerebral cortices of Sprague-Dawley rats), and Human Umbilical Cord Vein Endothelial Cells<br>In vivo: Male APP/PS1 (APPswe PS1dE9) transgenic mice, Male Wistar rats, Male C57BL/6J mice, B6.129P2-Nos3tm1Unc/J (eNOS-knockout) mice, 3xTg-AD mice, and Male Sprague–Dawley rats	In vitro: Excitotoxicity, cytotoxicity, and hypoxia/reoxygenation<br>In vivo: TBI, PD, chronic restrain stress, brain toxicity, SAH, intracranial hemorrhage, MCAO, vascular dementia, AD model, spinal cord lesion, retinal, liver, or cardiac I/R, ischemia, cardiac arrest, heart failure, and hypertension	**NaHS:** 50 µmol/kg, i.p. NaHS once daily for 4 weeks post-injury [7a], 5.6 mg/kg, i.p. NaHS once daily for 3 weeks post-injury [7b, 10a], 50 µmol/kg, i.p. NaHS pre-surgery/once daily for 5 d post-surgery [9b], 3 mg/kg NaHS i.p 5 min post-surgery [11a], 50 and 100 µmol/kg intracerebral injection NaHS daily for 8 weeks post-injury [11b], 200–800 µM NaHS added to culture media 1 h pre-injury [12a], and 56 µmol/kg i.p. NaHS 30 min pre-closure of surgical incision [12b]<br>**Diallyl Trisulfide (DATS):** 200 µg/kg retro-orbital injection, DATS once during study [9a]<br>**Na_2S:** 0.5-1.0 mg/kg retro-orbital injection, Na_2S twice daily [8a], and 6-12 mg/kg, i.p. Na_2S once daily 6 d pre-injury [10b]<br>**H_2S donor (NaGYY):** 100 mg/kg, i.p. NaGYY once daily for 12 weeks post-injury [8b]<br>**H_2S donor (PTA-NO-NPs):** 0.5-1 mg/mL PTA-NO-NPs added to culture media [13b]	Same as above
NO	In vitro: NO donors: SNAP, Spermine NONOate, PTA-NO-NPs, and NOC-18<br>In vivo: NO Donor ($NaNO_2$), NO Gas, and NO Synthesis Stimulator (L-arginine)	In vitro: Human Epidermal Stem Cells, Human Umbilical Cord Vein Endothelial Cells, and Human Embryonic Kidney 293 cells<br>In vivo: Male Sprague–Dawley rats, Neonatal Sprague–Dawley rats, Infant Yorkshire piglets, and Male Wistar rats	In vitro: Excitotoxicity, OGD, endothelial oxidative stress, mechanical nerve injury, and angiogenesis<br>In vivo: Excitotoxicity, DHCA, cerebral malaria, acute kidney injury, skin burn, liver, and lung transplant or I/R	**NO donors (SNAP, spermine NONOate)**: 100 µM SNAP/10 µM spermine NONOate added to culture media for 48 h post-injury [13a]<br>**NO donor (PTA-NO-NPs)**: 0.05-1 mg/mL PTA-NO-NPs added to culture media [13b]<br>**NO donor ($NaNO_2$):** 4 µmol, i.v. $NaNO_2$ 5 min post-injury [14]<br>**NO donor (NOC-18):** 300 µM bath perfusion Noc-18 added to culture media [15]<br>**NO gas:** 5-20 ppm inhalation NO and <1 ppm NO_2 for 7 d [16a], and 20 ppm inhalation NO for 3 h post-injury [16b]<br>**NO stimulator (L-arginine):** 50 mg/kg L-arginine i.v. in the anhepatic phase [17]	**NO donors (SNAP/spermine NONOate)**: 5000µM SNAP/100 µM NONOate for 48 h post-injury<br>**NO donor (PTA-NO-NPs:** ≥1 mg/mL added to culture media

H_2	In vitro: H_2 Gas<br>In vivo: H_2 Gas, and Hydrogen-rich Saline	In vitro: Primary Hepatocytes (from Male C57BL/6 (B6) mice), and Hippocampal neurons (from newborn Sprague-Dawley rats)<br>In vivo: Pregnant ICR (CD-1) mice, Male C57BL/6 (B6) mice, Male Sprague-Dawley, and Neonatal BALB/c mice	In vitro: OGD/reoxygenation, oxidative stress, and hypoxia and/or reoxygenation<br>In vivo: Spinal cord, cardiac, hepatic, retinal and cerebral I/R, cardiac arrest, birth asphyxia, MCAO, SAH, SCI, toxicity, and STZ-induced diabetes	**Hydrogen-rich saline:** 0.4 mM hydrogen-rich water 200 ml/kg/d x 14 [18a], 1 mL 7 ppm hydrogen-rich saline spread on directly on liver pre-injury [18b], 0.16 mol/kg, i.p. hydrogen-rich saline post-surgery [20a], 3 μmol/kg, i.p. hydrogen-rich saline daily 5 d pre-surgery [20b], and 5 ml/kg, i.p. hydrogen saline 3 d post-injury [22]<br>**H_2 gas:** 21% O_2, 5% CO_2, 3.8% H_2, and N_2 gas mixture added to culture media for 10 h [18b], 67% H_2-33% O_2 mixed gas (1 h inhalation/d x 1, 3 or 7) [19a, 19b], 2% H_2 gas inhalation for 1-2 h post-injury [20c], 1-3% H_2 gas inhalation 10 min pre-injury and 130 min post-injury [20d], and 60% H_2, 10% O_2, 5% CO_2, and 25% N_2 gas mixture inhalation for 24 h post-injury [21]	All medical gas dosages were well tolerated and safe in the experimental models investigated (see *Left Columns)*. For the purposes of the cited studies (**Table 2**), no toxic doses (gases in further higher concentrations) were evaluated
O_2	In vitro: Hyperbaric O_2<br>In vivo: Hyperbaric O_2	In vitro: Human Umbilical Cord Vein Endothelial Cells, and Spinal cord neurons (from embryonic Sprague-Dawley rats)<br>In vivo: Male Sprague-Dawley rats, C57B and SV129 mice, and Male Wistar rats	In vitro: Osteoarthritic chondrocytes, dermal fibroblasts, and muscle progenitor cells<br>In vivo: Nerve injury, SCI, AD model, MCAO, TBI, cerebral I/R, hepatectomy, muscle injury, ischemic, and tumor model	**Hyperbaric O_2:** 2.5 atmospheres 100% O_2 inhalation (1 h/d x 5 post-surgery) [23a], 2 atmospheres 95% O_2 inhalation (1 h/b.i.d. in first 3 d and q.d. x 11 d post-surgery) [23b], 1-2.5 atmospheres 98% O_2 added to culture media for 2-8 h [23c], 280 kPa HBO added to culture media for 60 min [24], 2.0 atmospheres 100% O_2 1 h inhalation x 4/d pre-injury [25], and 2.5 atmospheres 100% O_2 inhalation (2 h/d x 5 post-injury) [26]	Same as above
O_3	In vitro: None<br>In vivo: Ozone Gas, and Ozone Oil	In vitro: None<br>In vivo: C57B/L6 mice, and Male Sprague-Dawley rats	In vitro: Endothelial cells<br>In vivo: Pam3CYS inflammatory induction in the lungs, streptozotocin-induced diabetic model, and skin wound model	**Ozone gas:** 2 ppm ozone for 3 h pre-injury [27], 50 μg/ml ozone (q.d. x 15 post-injury) [28]<br>**Ozone oil:** 400 μl ozone oil applied once every 2 d x 12 post-lesion [29]	Same as above
	Noble Gases				
Xenon (Xe)	In vitro: Xe Gas<br>In vivo: Xe Gas, Xe-ELIP	In vitro: Mixed cortical glial-neuronal cocultures (from BALB/c mice), Midbrain cultures (from embryos of female Wistar rats), and hearts (from Male New Zealand rabbits)<br>In vivo: Sprague-Dawley rats, Male Wistar rats, and C57BL/6J mice	In vitro: OGD, hypoxia, excitotoxicity, and mechanical trauma<br>In vivo: TBI, I/R of spinal cord, MCAO, neonatal hypoxic-ischemic injury, and cardiac I/R	**Xe gas:** 12.5-70% Xe gas inhalation for 90 min post-injury [30a], 12.5-70% Xe gas added to culture media [30a], 70-75% Xe gas for 1 h post-injury [30b], 75% Xe gas applied to culture media for 3 d [31], 70% Xe gas inhalation for 3 h pre-injury [33], and 75% Xenon gas for 1 h pre-injury [34]<br>**Xe-ELIP:** 7-14 mg/kg (in 200 μL; i.a./intra-common carotid artery) of Xe-ELIP administered at 2, 3, or 5 h post-injury) [32]	Same as above
Argon (Ar)	In vitro: Ar Gas<br>In vivo: Ar Gas	In vitro: Human Neuroblastoma Cells, and Human Cardiac Myocyte-like Progenitor Cells<br>In vivo: Sprague-Dawley rats, and Male New Zealand rabbits	In vitro: Rotenone-induced apoptosis, LPS induction of microglia, OGD, OGD-reperfusion.<br>In vivo: MCAO, retinal I/R, kidneys, transplant, and multiorgan failure	**Ar gas:** 25-75% Ar gas inhalation for 2-4 hours post-injury [35a] 75% Ar gas inhalation for 60 min post-injury [35b], 75% Ar gas added to culture media for 2 h post-injury [35], 30-50% Ar gas added to culture media for 90 min pre-injury [36], 70% Ar gas inhalation 30 min pre-injury to 300 min post-injury [37a], and 2.06-2.14 MPa Ar gas [37b]	Same as above
Helium	In vitro: None	In vitro: None	In vivo: Brain hypoxic-	**He gas:** 70% He gas inhalation for 5 min x 3 periods pre-	Same as above

(He)	In vivo: He Gas	In vivo: Rat pups (7 d/o), Male Wistar rats, C57BL/6J mice, Male and New Zealand white rabbits	ischemic injury, cardiac arrest, and cardiac I/R	injury[38], 70% He gas inhalation for 5, 15, or 30 min post-injury [39], 70% He gas inhalation for 30 min pre-injury [40], and 70% He gas inhalation for 5 min x 1, 3, or 5 times post-injury [41]

Table S2: (1) Abbreviations and reference citations (same as **Table 2**) in Section S3. (2) For citation numbers representing more than one references, citation of the number only (e.g., [20] versus [20a] or [20d]) indicated that all references under that number (e.g., all 4 papers under [20]) were included.

Table S3

Analysis of Statistical Methods Used in All Medical Gas Studies Exhibited in Table 2

Gases	Data Presentation	Model and Sample Size	Power Analysis	Statistical Test	*Post-hoc* Analysis	Significance Level	Statistical Software
**Biological Gases**							
**CO**	Mean ± SD [1, 2, 4, 5a, 6]<br>Mean ± SEM [3, 5b & c]	**In vitro:**<br>n = 3 [1a]<br>n = 3-4 [2]<br>n = 4 [5b & c]<br>n = 3-6 [4]<br>n = 5-6 [6]<br>n = 7 [5c]<br>n = 6-8 [5b]<br>**In vivo:**<br>n = 3-7 [6]<br>n = 6 [1b]<br>n = 8 [5a]<br>n = 5-10 [3]<br>n = 10-13 [6]<br>n = 9-15 [6]	1[6]/8<br>(12.5%)*<br>*Percentage of studies that conducted power analysis	One way ANOVA [1-3, 5, 6]<br>Two way ANOVA [6]<br>Kruskal-Wallis test [3, 5a]<br>Student's *t*-test [4, 5c]	Bonferroni test [1a]<br>Scheffé test [1a]<br>Tukey's test [1b-3, 6]<br>Mann-Whitney U test [3]<br>Holm-Sidak test [5a, 6]<br>Fisher's exact test [5b]<br>Newman-Keuls test [5c]<br>Paired *t*-test [6]	*p*-value<br>($p < 0.005$-0.05) [1-6]<br>α-value<br>($\alpha = 0.05$) [6]<br>β-value<br>($\beta = 0.2$) [6]	GraphPad Prism 6<br>(GraphPad Software, San Diego, CA, USA) [2, 6]<br>GraphPad Prism 8<br>(GraphPad Software, San Diego, CA, USA) [1b]<br>SigmaPlot 11.0 (Systat Software Inc., San Jose, CA, USA) [5a]<br>SPSS 22.0 (IBM, Armonk, NY, USA) [5c]
**H_2S**	Mean ± SD [12a]<br>Mean ± SEM [7a-11b, 12b]	**In vitro:**<br>n = 3 [12a]<br>n = 3-4 [8b]<br>n = 4 [7b, 9a]<br>n = 4-5 [9b]<br>n = 7 [9b]<br>n = 12 [12b]<br>**In vivo:**<br>n = 4-12 [8a]<br>n = 5 [7a]<br>n = 6 [7b, 8b, 10-11b]<br>n = 8 [10b]<br>n = 12 [11b]<br>n = 18 [10a]	0/12 (0%)	One way ANOVA [7, 8b-12a]<br>Two way ANOVA [7b, 9a, 11, 12b]<br>Mann-Whitney U test [8a, 9a]<br>Kruskal-Wallis test [8a, 9a]<br>Student's *t*-test [7a, 8b, 9a]<br>Two-tailed independent *t*-test [10a]	Bonferroni test [7b, 9b, 10a, 11, 12b]<br>Tukey's test [7b, 8b, 9a, 10b]<br>Dunn's test [8a, 9a]<br>Games-Howell test [10a]<br>Fisher's LSD [11a]<br>Newman-Keuls test [12a]<br>Dunnett's test [12a]	*p*-value<br>($p < 0.001$-0.05) [7-12b]	SPSS 13.0 (IBM, Armonk, NY, USA) [9b]<br>SPSS 16.0 (IBM, Armonk, NY, USA) [7b, 11a, 12b]<br>SPSS 19.0 (IBM, Armonk, NY, USA) [9b, 10a, 12a]<br>SPSS 20.0 (IBM, Armonk, NY, USA) [11b]<br>SPSS 22.0 (IBM, Armonk, NY, USA) [10b]<br>GraphPad Prism<br>(GraphPad Software, San Diego, CA, USA) [8a, 9a]
**NO**	Mean ± SD [13a, 14]<br>Mean ± SEM [13b, 15-17]	**In vitro:**	0/7 (0%)	One way ANOVA [13, 14, 16a]<br>Two way ANOVA [14]	Newman-Keuls test [16a]<br>F-test [17]	*p*-value<br>($p < 0.001$-0.05) [13-17]	GraphPad Prism<br>(GraphPad Software, San Diego, CA,

		n = 3 [13a]<br>n = 3-4 [13b]<br>n = 3-8 [15]<br>n = 6-8 [15]<br>n = 12-16 [14]<br>**In vivo:**<br>n = 3-5 [14]<br>n = 4-5 [14]<br>n = 4-9 [14]<br>n = 5 [16b]<br>n = 6-10 [14]<br>n ≥ 12 [16a]<br>n = 18-19 [14]<br>n = 22 [17]		Mann-Whitney U test [14, 16b]<br>Kruskal-Wallis test [14]<br>Student's *t*-test [13a, 15]<br>Two-tailed unpaired *t*-test [16b]<br>Two-tailed Chi-squared test [17]			USA) [13b]<br>GraphPad Prism 4.0 (GraphPad Software, San Diego, CA, USA) [16a]<br>GraphPad Prism 5.0 (GraphPad Software, San Diego, CA, USA) [16b]<br>GraphPad Prism 6.05 (GraphPad Software, San Diego, CA, USA) [14]
**H_2**	Mean ± SD [19b-20a, 20c-21]<br>Mean ± SEM [18-19a, 20b]<br>Median [20d]	**In vitro:**<br>n = 3 [18b, 21]<br>n = 5-7 [18a]<br>n = 6 [20b]<br>n = 8 [20b]<br>**In vivo:**<br>n = 3-4 [20c, 22]<br>n = 4 [20a]<br>n = 5-7 [18b]<br>n = 6 [19a, 20c & d, 22]<br>n = 14 [20a]<br>n = 16-23 [19b]<br>n = 20 [18a]	1[20d]/10 (10%)	One way ANOVA [18a-22]<br>Two way ANOVA [20d]<br>Mann-Whitney U test [18a]<br>Kruskal-Wallis test [18a]<br>Student's *t*-test [18a]<br>Bivariate analysis [20a]<br>Chi-squared test [18a]	Bonferroni test [20c]<br>Tukey's test [18a, 19a, 20b, 20d, 22]<br>Dunn's test [18a]<br>Student's *t*-test [18b]<br>Dunnett's test [20a]<br>F-test [20c]<br>Fisher's LSD test [21]	*p*-value ($p < 0.001$-0.05) [18-22]<br>α-value ($\alpha = 0.01$) [20d]	GraphPad Prism 5.0 (GraphPad Software, San Diego, CA, USA) [18a]<br>SPSS 13.0 (IBM, Armonk, NY, USA) [20a, 20b]<br>SPSS 16.0 (IBM, Armonk, NY, USA) [19b]<br>SPSS 19.0 (IBM, Armonk, NY, USA) [19a, 21]<br>EZAnalyze (Microsoft, Redmond, WA, USA) [20c]<br>Statflex 6.0 (ARTECH Co, LTD, Osaka, Japan) [20d]<br>Sigmaplot 13.0 (Systat Software Inc., San Jose, CA, USA) [20d]
**O_2**	Mean ± SD [23-25]<br>Mean ± SEM [26]	**In vitro:**<br>n ≥ 3 [24]<br>**In vivo:**<br>n = 3 [23c]<br>n = 5 [23a]<br>n = 5-6 [26]<br>n = 6 [23b]<br>n = 6-8 [25]<br>n = 6-13 [26]	0/6 (0%)	One way ANOVA [23-26]<br>Two way ANOVA [26]<br>Kruskal-Wallis test [18a]<br>Student's *t*-test [23a, 26]<br>Independent *t*-test [23b]<br>Welch's *t*-test [26]	Bonferroni test [26]<br>Newman-Keuls test [25]<br>Fisher's LSD test [23c, 24]	*p*-value ($p < 0.01$-0.05) [23-26]	SPSS 17.0 (IBM, Armonk, NY, USA) [23b, 25]<br>SPSS 18.0 (IBM, Armonk, NY, USA) [24]<br>SPSS 23.0 (IBM, Armonk, NY, USA) [26]
**O_3**	Mean ± SD [28-29]	**In vitro:**	0/3 (0%)	One way ANOVA [29]	N/A	*p*-value	GraphPad Prism

	Mean ± SEM [27]	n ≥ 3 [29]<br>**In vivo:**<br>n = 4 [27]<br>n = 6 [28]<br>n = 10-15 [27]<br>n = 24 [29]		Mann-Whitney U test [27]<br>Student's *t*-test [28, 29]		(p < 0.001-0.05) [27-29]	(GraphPad Software, San Diego, CA, USA) [27]<br>GraphPad Prism 5.0<br>(GraphPad Software, San Diego, CA, USA) [29]<br>SPSS 18.0 (IBM, Armonk, NY, USA) [28]
**Noble Gases**							
**Xenon (Xe)**	Mean ± SD [30a, 31, 32, 34]<br>Mean ± SEM [33]<br>Mean [30b]	**In vitro:**<br>n = 4 [30a, 33]<br>n = 6-16 [31]<br>**In vivo:**<br>n = 4 [32]<br>n = 4-5 [30b]<br>n = 4-6 [30a, 33]<br>n = 8-9 [32]<br>n = 10-20 [34]	0/6 (0%)	One way ANOVA [30, 31, 33, 34]<br>Two way ANOVA [34]<br>Kruskal-Wallis test [31, 32]<br>Kaplan-Meier test [30a]<br>Wilcoxon rank sum test [32]	Bonferroni test [34]<br>Newman-Keuls test [30a, 31, 33]<br>Unpaired *t*-test [30a]<br>Mann-Whitney U test [30a]<br>*Post-hoc t*-test [34]	*p*-value<br>(p < 0.0005-0.01) [30-34]	Sigmastat 3.1<br>(Systat Software Inc., San Jose, CA, USA) [30b]<br>SigmaPlot 12.5 (Systat Software, San Jose, CA, USA) [31]<br>Statistica (Statsoft Inc., Tulsa, OK, USA) [32]<br>SPSS 16.0 (IBM, Armonk, NY, USA) [34]
**Argon (Ar)**	Mean ± SD [35, 36]<br>Mean ± SEM [37]<br>Median [37a]	**In vitro:**<br>n = 3 [36]<br>n = 4-8 [37b]<br>n = 5-8 [35a]<br>n = 6 [35b]<br>**In vivo:**<br>n = 4-8 [35b]<br>n = 7 [37a]	0/5 (0%)	One way ANOVA [35]<br>Two way ANOVA [36-37a]<br>Student's *t*-test [36, 37a]<br>Mann-Whitney U test [37b]<br>Kruskal-Wallis test [35, 37a]	Bonferroni test [36]<br>Holm-Sidak test [35, 37a]<br>Mann-Whitney U test [37a]<br>Newman-Keuls test [35a]	*p*-value<br>(p < 0.0001-0.05) [35-37b]	SigmaPlot 11.0 (Systat Software, San Jose, CA, USA) [35, 37b]<br>GraphPad Prism 6.0<br>(GraphPad Software, San Diego, CA, USA) [36]
**Helium (He)**	Mean ± SD [38, 40, 41]<br>Mean ± SEM [39]	**In vitro:**<br>N/A<br>**In vivo:**<br>n = 4-8 [39]<br>n = 4-9 [40]<br>n = 6-7 [41]<br>n = 7-16 [38]<br>n = 6 [41]<br>n = 7 [39-40]<br>n = 16 [38]	1[41]/4 (25%)	One way ANOVA [38, 40, 41]<br>Two way ANOVA [40]<br>Kruskal-Wallis test [39]<br>Welch's *t*-test [40]<br>Mann-Whitney U test [40]	Bonferroni test [40, 41]<br>Dunn's test [39]<br>Newman-Keuls test [38]	*p*-value<br>(p < 0.01-0.05) [38-41]<br>α-value<br>($\alpha = 0.05$) [41]<br>β-value<br>($\beta = 0.2$) [6]	SPSS 16.0 (IBM, Armonk, NY, USA) [38]<br>GraphPad Prism 5.0<br>(GraphPad Software, San Diego, CA, USA) [39]<br>GraphPad Prism 5.01<br>(GraphPad Software, San Diego, CA, USA) [40]

Table S3: (1) Abbreviations: ANOVA, analysis of variance; SD, standard deviation; SEM, standard error of mean; N/A, not available. (2) Reference citations (same as **Table 2**) in Section S3. (3) For citation numbers representing more than one references, citation of the number only

(e.g., [20] versus [20a] or [20d]) indicated that all references under that number (e.g., all 4 papers under [20]) were included. (4) All research papers enrolled in **Table 2** (n = 62; see Section S3) were systematically evaluated regarding statistical methods utilized, which included data presentation format, sample size, utilization of power analysis, specific statistical methods, *post-hoc* analysis, significance level setting, and statistical software used for computation.

Table S4

Data Pre-Processing in Medical Gas Studies Exhibited in Table 2

Gases	Data Pre-processing	Method of Data Quality Analysis
**Biological Gases**		
Carbon Monoxide (CO)	Normalization [1-6]<br>Percentage [1a, 3, 4]<br>Ratio [5a]	Shapiro-Wilk test for normality [1b]<br>Levene's test to assess the equality of variances for a variable calculated for two or more groups [1b]
Hydrogen (H_2)	Normalization [18, 19b, 20d, 22]<br>Percentage [18a, 19a, 20a, 21-22]<br>Ratio [19a, 22]<br>Transformation [20d]	Shapiro-Wilk test [20d]<br>Brown-Forsythe test for the equality of group variances based on performing an ANOVA on a transformation of the response variable [20d]
Hydrogen Sulfide (H_2S)	Normalization [8a, 10]<br>Percentage [7-8a, 10a, 11a]<br>Ratio [8a, 10a]<br>Homoscedasticity [10a]	Shapiro-Wilk test [10a]<br>Levene's test [10a]
Nitric Oxide (NO)	Normalization [13a, 14-16a]<br>Percentage [14, 15]<br>Ratio [13a, 14, 16a]	Not specified
Oxygen (O_2)	Normalization [23a, 23c, 26]<br>Percentage [26]<br>Ratio [23b & c, 26]<br>Homoscedasticity [26]	Shapiro-Wilk test [26]<br>Levene's test [26]
Ozone (O_3)	Normalization [27, 29]<br>Log2-transformation [27]<br>Ratio [28, 29]	Quantile Normalization to eliminate or minimize technical variability [27]
**Noble Gases**		
Argon (Ar)	Normalization [35, 36]<br>Percentage [35, 36, 37b]<br>Ratio [35]	Kolmogorov-Smirnov test, a nonparametric goodness-of-fit test used to determine if two distributions differ, or an underlying probability distribution differs from a hypothesized one [36]
Helium (He)	Normalization [38-40]<br>Percentage [41]<br>Ratio [38-40]	D'Agostino and Pearson test to assessing normality of data using skewness and kurtosis [40]
Xenon (Xe)	Normalization [30a & b, 32-33]<br>Percentage [30-32, 34]<br>Ratio [30a, 32]<br>Rank [32]<br>Transformation [32]	Not specified

Table S4: (1) Reference citations (same as **Table 2**) in Section S3. (2) For citation numbers representing more than one references, citation of the number only (e.g., [20] versus [20a] or [20d]) indicated that all references under that number (e.g., all 4 papers under [20]) were included. (3) All research papers enrolled in **Table 2** (n = 62; see Section S3 for reference citations) were assessed for methods of data pre-processing to evaluate data quality. Normalization, transformation, and homoscedasticity were data pre-processing procedures that were utilized in 34/62 (55%) of the reports reviewed in **Table 2**. Methods applied for data quality analysis were Brown-Forsythe test, D'Agostino and Pearson test, Kolmogorov-Smirnov test, Levene's test, Quantile Normalization, and Shapiro-Wilk test (see more details in the table above). However, the cited studies on CO (n = 8), NO (n = 4, and Xe (n = 4) did not specify statistical methods used in data normalization.

Figure S1

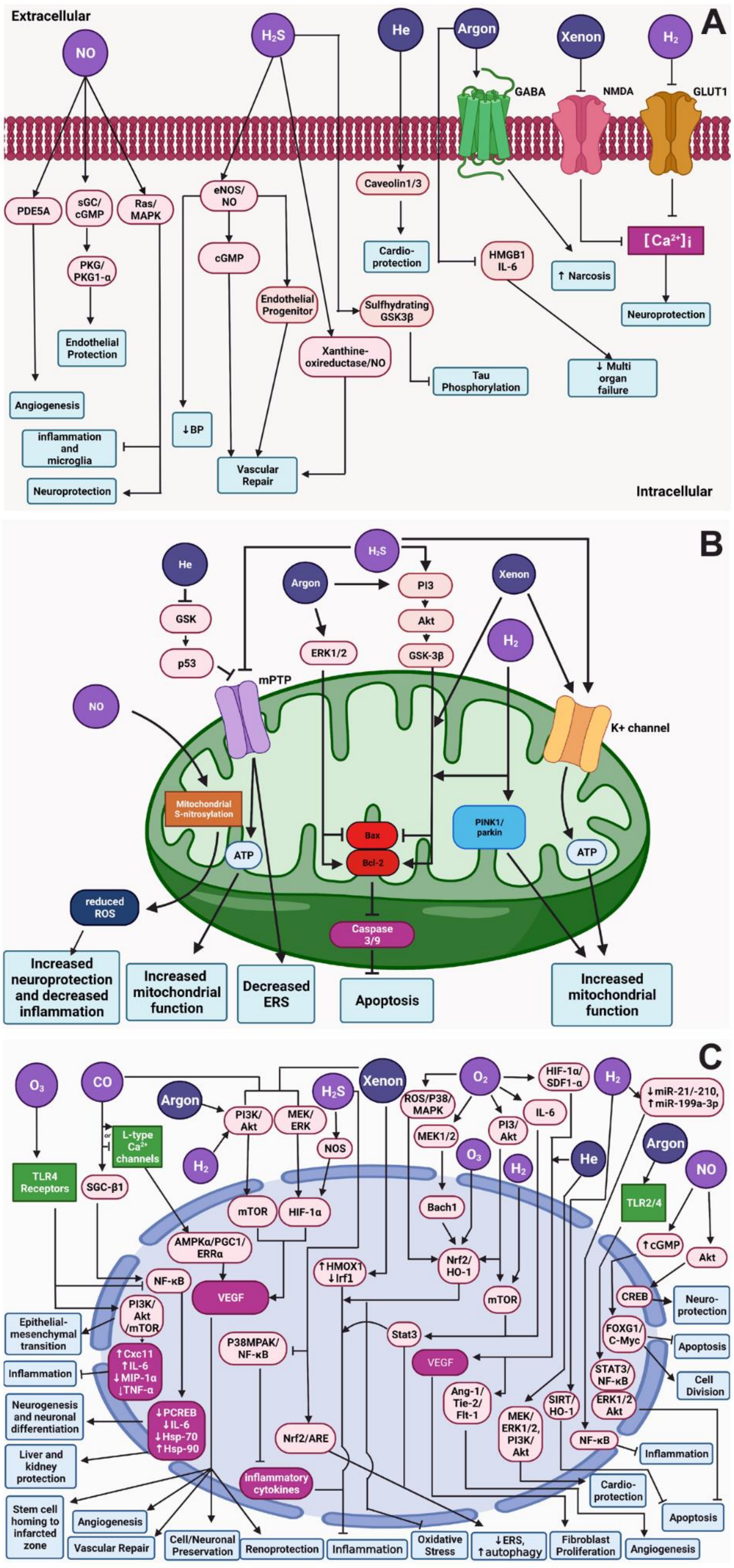

Figure S1. The key signal transduction pathways and molecular mechanisms underlying the primary tissue protective effects of the medical gases reviewed. **Figure S1** illustrated the main signal transduction pathways, cell signalling convergence, divergence and crosstalk, and key messenger, effector or target molecules underlying the primary tissue protective mechanisms of the medical gases reviewed in **Table 2**. To avoid redundancy with **Figures 4** and **5**, only the therapeutic mechanisms of 3 biological gases (H_2, H_2S, and NO) and 3 noble gases (Argon/Ar, Helium/He, and Xenon/Xe) were detailed at cytoplasmic (**A**) and mitochondrial (**B**) levels; primary nuclear level mechanisms for tissue protection of all six biological gases (CO, H_2, H_2S, NO, O_2, and O_3) and 3 noble gases (Ar, He, and Xe) were shown in **C** (note: for summaries on tissue protective mechanisms of medical gases, see **Table 2**; abbreviations, acronyms, and reference citations, see Section S3; for cytoplasmic and mitochondrial mechanisms of CO, O_2 and O_3, see **Figures 4** and **5**; **Figure S1** plotting was assisted by BioRender.com).

Section S1: Supporting Information

Database Search Strategies

I. Limitation for all database: (1) publications from January 1, 2000 to July 31, 2021

(2) literature published in English

II. Search Criteria for Ovid MEDLINE, using Medical Subject Headings (MeSH)

1. Tissue preservation/
2. Tissue preservation.mp.
3. Tissue repair.mp.
4. Tissue protection.mp.
5. Neuroprotection/
6. Neural Protection.mp.
7. Regeneration/
8. Neuroregeneration.mp.
9. Neural Regeneratiom.mp.
10. Spinal cord regeneration/
11. Angioplasty/
12. Bone regeneration/
13. Bone protection.mp.
14. Liver regeneration/
15. Nerve regeneration/
16. Brain regeneration/
17. Neural Repair.mp.
18. Brain repair.mp.
19. Spinal cord repair.mp.
20. 1 OR 2 OR 3 OR 4 OR 5 OR 6 OR 7 OR 8 OR 9 OR 10 OR 11 OR 12 OR 13 OR 14 OR 15 OR 16 OR 17 OR 18 OR 19
21. Medical gas.mp.
22. Gases/
23. Oxygen/
24. Hydrogen/

25. Hydrogen Sulfide/

26. Carbon Monoxide/

27. Nitric Oxide/

28. Nitrous Oxide/

29. Ozone/

30. Noble gas/

31. Noble gas.mp.

32. Helium/

33. Xenon/

34. Krypton/

35. Argon/

36. 21 OR 22 OR 23 OR 24 OR 25 OR 26 OR 27 OR 28 OR 29 OR 30 OR 31 OR 32 OR 33 OR 34 OR 35

37. Signal transduction/

38. Signal transduction.mp.

39. Mechanism.mp.

40. Pathway.mp.

41. Signaling.mp.

42. 37 OR 38 OR 39 OR 40 OR 41

43. 20 AND 36 AND 42

III. The same search criteria was utilized for EMBASE for performing a focused search using MeSH

VI. Search Criteria for Scopus and Google Scholar

(Tissue AND Preservation) OR ("Tissue protection") OR (Tissue AND Repair)

AND

("Medical gas") OR (Oxygen AND Therapy) OR (Hydrogen AND Therapy) OR ("Hydrogen sulfide") OR ("Carbon monoxide") OR ("Nitrous Oxide") OR ("Nitric Oxide") OR (Ozone) OR ("Noble gas") OR (Helium) OR (Xenon) OR (Krypton) OR (Argon)

AND

("Signal transduction") OR (Mechanism) OR (Pathway)

Section S2: Supporting Information

Publications Qualified for Systematic Review Enrollment

(note: articles are listed in alphabetical order under each medical gas molecule)

- Biological gases

1. Carbon Monoxide [1-48]
2. Hydrogen [29, 49-74]
3. Hydrogen sulfide [75-129]
4. Nitric oxide [130-147]
5. Oxygen [148-172]
6. Ozone [173-180]

- Noble gases

1. Argon [181-191]
2. Helium [191, 192-197]
3. Xenon [191, 198-212]

- Reports on findings of adverse effects or no significant therapeutic impact of medical gases on some or all outcome measures [192, 199, 211, 213-223]

Biological Gases

Carbon Monoxide (CO)

1. Bai, J., Bai, Y., Wang, X.-P., Zheng, W.-C. & Zhang, L.-M. Carbon Monoxide-Releasing Molecule-3 Ameliorates Acute Lung Injury in a Model of Hemorrhagic Shock and Resuscitation: Roles of p38MAPK Signaling Pathway. *Shock* **55**, 816-826, doi: 10.1097/SHK.0000000000001684 (2021).
2. Biermann, J., Lagreze, W. A., Dimitriu, C., Stoykow, C. & Goebel, U. Preconditioning with inhalative carbon monoxide protects rat retinal ganglion cells from ischemia/reperfusion injury. *Invest Ophthalmol Vis Sci* **51**, 3784-3791, doi:http://dx.doi.org/10.1167/iovs.09-4894 (2010).
3. Carraway, M. S. *et al.* Carbon monoxide promotes hypoxic pulmonary vascular remodeling. *Am J Physiol Lung Cell Mol Physiol* **282**, L693-702, doi:10.1152/ajplung.00211.2001 (2002).
4. Choi, Y. K. *et al.* Carbon monoxide promotes VEGF expression by increasing HIF-1alpha protein level via two distinct mechanisms, translational activation and stabilization of HIF-1alpha protein. *J Biol Chem* **285**, 32116-32125, doi:10.1074/jbc.M110.131284 (2010).
5. Choi, Y. K. *et al.* Carbon Monoxide Potentiation of L-Type Ca^{2+} Channel Activity Increases HIF-1α-Independent VEGF Expression via an AMPKα/SIRT1-Mediated PGC-1α/ERRα Axis. *Antioxid Redox Signal* **27**, 20-36, doi:10.1089/ars.2016.6684 (2017).
6. Choi, Y. K. *et al.* Dual effects of carbon monoxide on pericytes and neurogenesis in traumatic brain injury. *Nat Med* **22**, 1335-1341, doi:10.1038/nm.4188 (2016).
7. Correa-Costa, M. *et al.* Carbon monoxide protects the kidney through the central circadian clock and CD39. *Proc Natl Acad Sci U S A* **115**, E2302-E2310, doi:10.1073/pnas.1716747115 (2018).
8. Dallas, M. L. *et al.* Carbon monoxide protects against oxidant-induced apoptosis via inhibition of Kv2.1. *FASEB J* **25**, 1519-1530, doi:http://dx.doi.org/10.1096/fj.10-173450 (2011).
9. Dreyer-Andersen, N. *et al.* Intermittent, low dose carbon monoxide exposure enhances survival and dopaminergic differentiation of human neural stem cells. *PLoS ONE* **13**, e0191207,

doi:10.1371/journal.pone.0191207 (2018).
10. Han, Y. *et al.* Carbon monoxide offers neuroprotection from hippocampal cell damage induced by recurrent febrile seizures through the PERK-activated ER stress pathway. *Neurosci Lett* **585**, 126-131, doi:http://dx.doi.org/10.1016/j.neulet.2014.11.040 (2015).
11. Horvat, A., Vardjan, N. & Zorec, R. Targeting Astrocytes for Treating Neurological Disorders: Carbon Monoxide and Noradrenaline-Induced Increase in Lactate. *Curr Pharm Des* **23**, 4969-4978, doi:10.2174/1381612823666170622112734 (2017).
12. Imuta, N. *et al.* Hypoxia-mediated induction of heme oxygenase type I and carbon monoxide release from astrocytes protects nearby cerebral neurons from hypoxia-mediated apoptosis. *Antioxid Redox Signal* **9**, 543-552, doi:10.1089/ars.2006.1519 (2007).
13. Jamal Uddin, M. *et al.* IRG1 induced by heme oxygenase-1/carbon monoxide inhibits LPS-mediated sepsis and pro-inflammatory cytokine production. *Cell Mol Immunol* **13**, 170-179, doi:10.1038/cmi.2015.02 (2016).
14. Joshi, H. P. *et al.* Nanocarrier-mediated Delivery of CORM-2 Enhances Anti-allodynic and Anti-hyperalgesic Effects of CORM-2. *Mol Neurobiol* **56**, 5539-5554, doi:10.1007/s12035-019-1468-7 (2019).
15. Józkowicz, A. *et al.* Heme oxygenase and angiogenic activity of endothelial cells: stimulation by carbon monoxide and inhibition by tin protoporphyrin-IX. *Antioxid Redox Signal* **5**, 155-162, doi:10.1089/152308603764816514 (2003).
16. Kaiser, S., Selzner, L., Weber, J. & Schallner, N. Carbon monoxide controls microglial erythrophagocytosis by regulating CD36 surface expression to reduce the severity of hemorrhagic injury. *Glia* **68**, 2427-2445, doi:http://dx.doi.org/10.1002/glia.23864 (2020).
17. Kaizu, T. *et al.* Protection of transplant-induced hepatic ischemia/reperfusion injury with carbon monoxide via MEK/ERK1/2 pathway downregulation. *Am J Physiol Gastrointest Liver Physiol* **294**, G236-G244, doi:10.1152/ajpgi.00144.2007 (2007).
18. Kaizu, T. *et al.* Carbon monoxide inhalation ameliorates cold ischemia/reperfusion injury after rat liver transplantation. *Surgery* **138**, 229-235, doi:10.1016/j.surg.2005.06.015 (2005).
19. Kim, J. H. *et al.* Hypoxia-responsive microRNA-101 promotes angiogenesis via heme oxygenase-1/vascular endothelial growth factor axis by targeting cullin 3. *Antioxid Redox Signal* **21**, 2469-2482, doi:10.1089/ars.2014.5856 (2014).
20. Kim, K. M. *et al.* Carbon monoxide induces heme oxygenase-1 via activation of protein kinase R-like endoplasmic reticulum kinase and inhibits endothelial cell apoptosis triggered by endoplasmic reticulum stress. *Circ Res* **101**, 919-927, doi:10.1161/circresaha.107.154781 (2007).
21. Kuramitsu, K. *et al.* Carbon monoxide enhances early liver regeneration in mice after hepatectomy. *Hepatology* **53**, 2016-2026, doi:https://dx.doi.org/10.1002/hep.24317 (2011).
22. Kuramitsu, K., Wegiel, B., Csizmadia, E., Otterbein, L. E. & Hanto, D. W. The effect of carbon monoxide on liver regeneration after hepatectomy. *Hepatology* **50**, 639A, doi:http://dx.doi.org/10.1002/hep.23303 (2009).
23. Lakkisto, P. *et al.* Heme oxygenase-1 and carbon monoxide promote neovascularization after myocardial infarction by modulating the expression of HIF-1α, SDF-1α and VEGF-B. *Eur J Pharmacol* **635**, 156-164, doi:10.1016/j.ejphar.2010.02.050 (2010).
24. Li, M., Gallo, D., Csizmadia, E., Otterbein, L. E. & Wegiel, B. Carbon monoxide induces chromatin remodelling to facilitate endothelial cell migration. *Thromb Haemost* **111**, 951-959, doi:10.1160/TH13-09-0748 (2014).
25. Lin, C. C., Yang, C. C., Hsiao, L. D., Chen, S. Y. & Yang, C. M. Heme Oxygenase-1 Induction by Carbon Monoxide Releasing Molecule-3 Suppresses Interleukin-1β-Mediated Neuroinflammation. *Front Mol Neurosci* **10**, 387, doi:10.3389/fnmol.2017.00387 (2017).
26. Lin, H. H., Chen, Y. H., Yet, S. F. & Chau, L. Y. After vascular injury, heme oxygenase-1/carbon monoxide enhances re-endothelialization via promoting mobilization of circulating endothelial progenitor cells. *J Thromb Haemost* **7**, 1401-1408, doi:10.1111/j.1538-7836.2009.03478.x (2009).

27. Lu, K. *et al.* Carbon Monoxide Releasing Molecule-3 (CORM-3) Regulates Microglia Activity, Prevents Neuronal Injury, and Improves Memory Function Dur-ing Radiation Induced Brain Injury. *Curr Neurovasc Res*, **17**, 464-470, doi:10.2174/1567202617999200730213259 (2020).
28. Nakao, A. *et al.* Ex vivo carbon monoxide delivery inhibits intimal hyperplasia in arterialized vein grafts. *Cardiovasc Res* **89**, 457-463, doi:https://dx.doi.org/10.1093/cvr/cvq298 (2011).
29. Nakao, A. *et al.* Amelioration of rat cardiac cold ischemia/reperfusion injury with inhaled hydrogen or carbon monoxide, or both. *J Heart Lung Transplant* **29**, 544-553, doi:10.1016/j.healun.2009.10.011 (2010).
30. Nakao, A. *et al.* Protection against ischemia/reperfusion injury in cardiac and renal transplantation with carbon monoxide, biliverdin and both. *Am J Transplant* **5**, 282-291, doi:10.1111/j.1600-6143.2004.00695.x (2005).
31. Ozaki, K. S. *et al.* Carbon monoxide inhibits apoptosis during cold storage and protects kidney grafts donated after cardiac death. *Transplant Int* **25**, 107-117, doi:10.1111/j.1432-2277.2011.01363.x (2012).
32. Pizarro, M. D. *et al.* Protective effects of a carbon monoxide-releasing molecule (CORM-3) during hepatic cold preservation. *Cryobiology* **58**, 248-255 (2009).
33. Queiroga, C. S. *et al.* Preconditioning triggered by carbon monoxide (CO) provides neuronal protection following perinatal hypoxia-ischemia. *PLoS ONE* **7**, e42632, doi:10.1371/journal.pone.0042632 (2012).
34. Ryter, S. W., Morse, D. & Choi, A. M. K. Carbon monoxide and bilirubin: Potential therapies for pulmonary/vascular injury and disease. *Am J Resp Cell Mol Biol* **36**, 175-182, doi:10.1165/rcmb.2006-0333TR (2007).
35. Schallner, N. *et al.* Carbon Monoxide Abrogates Ischemic Insult to Neuronal Cells via the Soluble Guanylate Cyclase-cGMP Pathway. *PLoS ONE* **8**, e60672, doi:http://dx.doi.org/10.1371/journal.pone.0060672 (2013).
36. Song, B. *et al.* Nano-designed carbon monoxide donor SMA/CORM2 exhibits protective effect against acetaminophen induced liver injury through macrophage reprograming and promoting liver regeneration. *J Control Release* **331**, 350-363, doi: 10.1016/j.jconrel.2021.01.025 (2021).
37. Stifter, J. *et al.* Neuroprotection and neuroregeneration of retinal ganglion cells after intravitreal carbon monoxide release. *PLoS ONE* **12**, e0188444, doi:https://dx.doi.org/10.1371/journal.pone.0188444 (2017).
38. Uddin, M. J., Jeong, J., Pak, E. S. & Ha, H. CO-Releasing Molecule-2 Prevents Acute Kidney Injury through Suppression of ROS-Fyn-ER Stress Signaling in Mouse Model. *Oxid Med Cell Longev* **2021**, 9947772, doi: 10.1155/2021/9947772 (2021).
39. Ulbrich, F. *et al.* The Carbon monoxide releasing molecule ALF-186 mediates anti-inflammatory and neuroprotective effects via the soluble guanylate cyclase ß1 in rats' retinal ganglion cells after ischemia and reperfusion injury. *J Neuroinflammation* **14**, 130, doi:10.1186/s12974-017-0905-7 (2017).
40. Ulbrich, F. *et al.* The CORM ALF-186 Mediates Anti-Apoptotic Signaling via an Activation of the p38 MAPK after Ischemia and Reperfusion Injury in Retinal Ganglion Cells. *PLoS ONE* **11**, e0165182, doi:10.1371/journal.pone.0165182 (2016).
41. Vieira, H. L., Queiroga, C. S. & Alves, P. M. Pre-conditioning induced by carbon monoxide provides neuronal protection against apoptosis. *J Neurochem* **107**, 375-384, doi:10.1111/j.1471-4159.2008.05610.x (2008).
42. Wang, B., Cao, W., Biswal, S. & Dore, S. Carbon monoxide-activated Nrf2 pathway leads to protection against permanent focal cerebral ischemia. *Stroke* **42**, 2605-2610, doi:http://dx.doi.org/10.1161/STROKEAHA.110.607101 (2011).
43. Wang, R. *et al.* Preconditioning with carbon monoxide inhalation promotes retinal ganglion cell survival against optic nerve crush via inhibition of the apoptotic pathway. *Mol Med Rep* **17**, 1297-1304, doi:http://dx.doi.org/10.3892/mmr.2017.7990 (2018).
44. Wang, X.-P. *et al.* Carbon Monoxide-Releasing Molecule-3 Alleviates Kupffer Cell Pyroptosis Induced by Hemorrhagic Shock and Resuscitation via sGC-cGMP Signal Pathway. *Inflammation* **44**, 1330-1344, doi: 10.1007/s10753-021-01419-w (2021).

45. Wegiel, B. *et al.* Nitric oxide-dependent bone marrow progenitor mobilization by carbon monoxide enhances endothelial repair after vascular injury. *Circulation* **121**, 537-548, doi:10.1161/CIRCULATIONAHA.109.887695 (2010).
46. Zhang, R. G., Yip, C. Y. & Ko, W. H. Regulation of Intracellular Calcium by Carbon Monoxide in Human Bronchial Epithelial Cells. *Cell Physiol Biochem* **42**, 2377-2390, doi:10.1159/000480029 (2017).
47. Zheng, G. *et al.* Carbon monoxide releasing molecule-3 alleviates neuron death after spinal cord injury via inflammasome regulation. *EBioMedicine* **40**, 643-654, doi:10.1016/j.ebiom.2018.12.059 (2019).
48. Zheng, G. *et al.* CO-Releasing Molecule (CORM)-3 Ameliorates Spinal Cord-Blood Barrier Disruption Following Injury to the Spinal Cord. *Front Pharmacol* **11**, 761, doi:http://dx.doi.org/10.3389/fphar.2020.00761 (2020).

Hydrogen (H_2)

49. Bai, X. *et al.* Hydrogen-rich saline mediates neuroprotection through the regulation of endoplasmic reticulum stress and autophagy under hypoxia-ischemia neonatal brain injury in mice. *Brain Res* **1646**, 410-417, doi:http://dx.doi.org/10.1016/j.brainres.2016.06.020 (2016).
50. Buchholz, B. M. *et al.* Hydrogen-enriched preservation protects the isogeneic intestinal graft and amends recipient gastric function during transplantation. *Transplantation* **92**, 985-992, doi:10.1097/TP.0b013e318230159d (2011).
51. Chen, X. *et al.* Inhalation of Hydrogen of Different Concentrations Ameliorates Spinal Cord Injury in Mice by Protecting Spinal Cord Neurons from Apoptosis, Oxidative Injury and Mitochondrial Structure Damages. *Cell Physiol Biochem* **47**, 176-190, doi:http://dx.doi.org/10.1159/000489764 (2018).
52. Hong, Y. *et al.* Neuroprotective effect of hydrogen-rich saline against neurologic damage and apoptosis in early brain injury following subarachnoid hemorrhage: Possible role of the Akt/GSK3beta signaling pathway. *PLoS ONE* **9**, e96212, doi:http://dx.doi.org/10.1371/journal.pone.0096212 (2014).
53. Huang, J. L. *et al.* Hydrogen inhibits microglial activation and regulates microglial phenotype in a mouse middle cerebral artery occlusion model. *Med Gas Res* **9**, 127-132, doi:10.4103/2045-9912.266987 (2019).
54. Huo, T. T. *et al.* Hydrogen-rich saline improves survival and neurological outcome after cardiac arrest and cardiopulmonary resuscitation in Rats. *Anesth Analg* **119**, 368-380, doi:10.1213/ANE.0000000000000303 (2014).
55. Imai, K. *et al.* Neuroprotective potential of molecular hydrogen against perinatal brain injury via suppression of activated microglia. *Free Radic Biol Med* **91**, 154-163, doi:http://dx.doi.org/10.1016/j.freeradbiomed.2015.12.015 (2016).
56. Ishikawa, T. *et al.* Post-reperfusion hydrogen gas treatment ameliorates ischemia reperfusion injury in rat livers from donors after cardiac death: a preliminary study. *Surg Today* **48**, 1081-1088, doi:10.1007/s00595-018-1693-0 (2018).
57. Jiao, Y. *et al.* Protective effects of hydrogen-rich saline against experimental diabetic peripheral neuropathy via activation of the mitochondrial ATP-sensitive potassium channel channels in rats. *Mol Med Rep* **21**, 282-290, doi:http://dx.doi.org/10.3892/mmr.2019.10795 (2020).
58. Kimura, A. *et al.* Protective effects of hydrogen gas against spinal cord ischemia-reperfusion injury. *J Thorac Cardiovasc Surg* S0022-5223(21)00762–5, doi:10.1016/j.jtcvs.2021.04.077 (2021).
59. Li, J. *et al.* Protective effects of hydrogen-rich saline in a rat model of permanent focal cerebral ischemia via reducing oxidative stress and inflammatory cytokines. *Brain Res* **1486**, 103-111, doi:http://dx.doi.org/10.1016/j.brainres.2012.09.031 (2012).
60. Li, Q. *et al.* Neuroprotective Effect of Hydrogen-Rich Saline in Global Cerebral Ischemia/Reperfusion Rats: Up-Regulated Tregs and Down-Regulated miR-21, miR-210 and NF-kappaB Expression. *Neurochem Res* **41**, 2655-2665, doi:http://dx.doi.org/10.1007/s11064-016-1978-x (2016).
61. Murakami, Y., Ito, M. & Ohsawa, I. Molecular hydrogen protects against oxidative stress-induced SH-

SY5Y neuroblastoma cell death through the process of mitohormesis. *PLoS ONE* **12**, e0176992, doi:http://dx.doi.org/10.1371/journal.pone.0176992 (2017).
62. Olah, O., Toth-Szuki, V., Temesvari, P., Bari, F. & Domoki, F. Delayed neurovascular dysfunction is alleviated by hydrogen in asphyxiated newborn pigs. *Neonatology* **104**, 79-86, doi:http://dx.doi.org/10.1159/000348445 (2013).
63. Spulber, S. *et al.* Molecular hydrogen reduces lps-induced neuroinflammation and promotes recovery from sickness behaviour in mice. *PLoS ONE* **7**, e42078, doi:http://dx.doi.org/10.1371/journal.pone.0042078 (2012).
64. Wang, L. *et al.* Hydrogen exerts neuroprotection by activation of the miR-21/PI3K/AKT/GSK-3beta pathway in an in vitro model of traumatic brain injury. *J Cell Mol Med* **24**, 4061-4071, doi:http://dx.doi.org/10.1111/jcmm.15051 (2020).
65. Wang, P. *et al.* Hydrogen Gas Attenuates Hypoxic-Ischemic Brain Injury via Regulation of the MAPK/HO-1/PGC-1a Pathway in Neonatal Rats. *Oxidative Med Cell Longev* **2020**, 6978784, doi:http://dx.doi.org/10.1155/2020/6978784 (2020).
66. Wang, T. *et al.* Oral intake of hydrogen-rich water ameliorated chlorpyrifos-induced neurotoxicity in rats. *Toxicol Appl Pharmacol* **280**, 169-176, doi:http://dx.doi.org/10.1016/j.taap.2014.06.011 (2014).
67. Wang, X., Zhang, L., Zhao, W. & Liu, T. The protective effects of hydrogen on HO-1 expression in the brain after focal cerebral ischemia reperfusion in rats. *Turk J Med Sci* **46**, 1534-1539, doi:http://dx.doi.org/10.3906/sag-1502-3 (2016).
68. Wei, R., Zhang, R., Xie, Y., Shen, L. & Chen, F. Hydrogen suppresses hypoxia/reoxygenation-induced cell death in hippocampal neurons through reducing oxidative stress. *Cell Physiol Biochem* **36**, 585-598, doi:http://dx.doi.org/10.1159/000430122 (2015).
69. Wu, J. *et al.* Hydrogen postconditioning promotes survival of rat retinal ganglion cells against ischemia/reperfusion injury through the PI3K/Akt pathway. *Biochem Biophys Res Commun* **495**, 2462-2468, doi:http://dx.doi.org/10.1016/j.bbrc.2017.12.146 (2018).
70. Wu, X. *et al.* Hydrogen exerts neuroprotective effects on OGD/R damaged neurons in rat hippocampal by protecting mitochondrial function via regulating mitophagy mediated by PINK1/Parkin signaling pathway. *Brain Res* **1698**, 89-98, doi:http://dx.doi.org/10.1016/j.brainres.2018.06.028 (2018).
71. Xie, K. *et al.* Hydrogen Gas Alleviates Sepsis-Induced Brain Injury by Improving Mitochondrial Biogenesis Through the Activation of PGC-α in Mice. *Shock* **55**, 100-109, doi: 10.1097/SHK.0000000000001594 (2021).
72. Zhang, J.-Y. *et al.* Hydrogen-rich water protects against acetaminophen-induced hepatotoxicity in mice. *World J Gastroenterol* **21**, 4195-4209, doi:https://dx.doi.org/10.3748/wjg.v21.i14.4195 (2015).
73. Zhao, Q. H. *et al.* Hydrogen inhalation inhibits microglia activation and neuroinflammation in a rat model of traumatic brain injury. *Brain Res* **1748**, 147053, doi:http://dx.doi.org/10.1016/j.brainres.2020.147053 (2020).
74. Zhuang, Z. *et al.* Nuclear factor-kappaB/Bcl-XL pathway is involved in the protective effect of hydrogen-rich saline on the brain following experimental subarachnoid hemorrhage in rabbits. *J Neurosci Res* **91**, 1599-1608, doi:http://dx.doi.org/10.1002/jnr.23281 (2013).

Hydrogen Sulfide (H_2S)

75. Bai, C. P., Zhao, C. & Shen, L. Post-treatment with a hydrogen sulfide donor limits neuronal injury and modulates potassium voltage-gated channel subfamily d member 2 (Kv4.2) and potassium channel interacting protein 3 (KChIP3) during transient global cerebral ischemia. *Curr Neurovascular Res* **14**, 397-405, doi:http://dx.doi.org/10.2174/1567202614666171108113447 (2017).
76. Biermann, J., Lagreze, W. A., Schallner, N., Schwer, C. I. & Goebel, U. Inhalative preconditioning with hydrogen sulfide attenuated apoptosis after retinal ischemia/reperfusion injury. *Mol Vision* **17**, 1275-1286 (2011).
77. Bir, S. C. *et al.* Hydrogen sulfide stimulates ischemic vascular remodeling through nitric oxide synthase

and nitrite reduction activity regulating hypoxia-inducible factor-1α and vascular endothelial growth factor-dependent angiogenesis. *J Am Heart Assoc* **1**, e004093, doi:10.1161/jaha.112.004093 (2012).
78. Chen, L., Ma, K., Fan, H., Wang, X. & Cao, T. Exogenous hydrogen sulfide protects against hepatic ischemia/reperfusion injury by inhibiting endoplasmic reticulum stress and cell apoptosis. *Exp Ther Med* **22**, 799, doi: 10.3892/etm.2021.10231 (2021).
79. Chen, S. M. *et al.* Hydrogen sulfide attenuates postoperative cognitive dysfunction through promoting the pathway of Warburg effect-synaptic plasticity in hippocampus. *Toxicol Appl Pharmacol* **409**, 115286, doi:http://dx.doi.org/10.1016/j.taap.2020.115286 (2020).
80. Chen, Y. *et al.* Inhibition of ALDH2 protects PC12 cells against formaldehyde-induced cytotoxicity: involving the protection of hydrogen sulphide. *Clin Exp Pharmacol Physiol* **44**, 595-601, doi:http://dx.doi.org/10.1111/1440-1681.12741 (2017).
81. Ciocci, M. *et al.* H2S-releasing nanoemulsions: A new formulation to inhibit tumor cells proliferation and improve tissue repair. *Oncotarget* **7**, 84338-84358, doi:10.18632/oncotarget.12609 (2016).
82. Giovinazzo, D. *et al.* Hydrogen sulfide is neuroprotective in Alzheimer's disease by sulfhydrating GSK3β and inhibiting Tau hyperphosphorylation. *Proc Natl Acad Sci U S A* **118**, e2017225118, doi: 10.1073/pnas.2017225118 (2021).
83. Giuliani, D. *et al.* Hydrogen sulfide slows down progression of experimental Alzheimer's disease by targeting multiple pathophysiological mechanisms. *Neurobiol Learn Mem* **104**, 82-91, doi:10.1016/j.nlm.2013.05.006 (2013).
84. Greasley, A. *et al.* H2S protects against cardiac cell hypertrophy through regulation of selenoproteins. *Oxidative Med Cell Longev* **2019**, 6494306, doi:10.1155/2019/6494306 (2019).
85. Hazzaa, S. M. *et al.* H2S releasing Sodium sulfide protects from acute stress-induced hypertension by increasing the activity of endothelial nitric oxide synthase enzyme. *Tissue Cell* **72**, 101550, doi: 10.1016/j.tice.2021.101550 (2021).
86. He, X. L. *et al.* Hydrogen sulfide down-regulates BACE1 and PS1 via activating PI3K/Akt pathway in the brain of APP/PS1 transgenic mouse. *Pharmacol Rep* **68**, 975-982, doi:http://dx.doi.org/10.1016/j.pharep.2016.05.006 (2016).
87. Hu, Q. *et al.* Hydrogen sulfide improves vascular repair by promoting endothelial nitric oxide synthase-dependentmobilization of endothelial progenitor cells. *J Hypertens* **37**, 972-984, doi:10.1097/HJH.0000000000001983 (2019).
88. Huang, S. *et al.* Hydrogen sulfide supplement attenuates the apoptosis of retinal ganglion cells in experimental glaucoma. *Exp Eye Res* **168**, 33-48, doi:10.1016/j.exer.2018.01.004 (2018).
89. Jiang, L. H., Luo, X., He, W. A., Huang, X. X. & Cheng, T. T. Effects of exogenous hydrogen sulfide on apoptosis proteins and oxidative stress in the hippocampus of rats undergoing heroin withdrawal. *Arch Pharm Res* **34**, 2155-2162, doi:http://dx.doi.org/10.1007/s12272-011-1220-y (2011).
90. Jiang, X., Huang, Y., Lin, W., Gao, D., Fei, Z. Protective effects of hydrogen sulfide in a rat model of traumatic brain injury via activation of mitochondrial adenosine triphosphate-sensitive potassium channels and reduction of oxidative stress. *J Surg Res* **184**, E27-E35, doi:http://dx.doi.org/10.1016/j.jss.2013.03.067 (2013).
91. Kang, B. *et al.* Involvement of miR-1 in the protective effect of hydrogen sulfide against cardiomyocyte apoptosis induced by ischemia/reperfusion. *Mol Biol Rep* **41**, 6845-6853, doi:http://dx.doi.org/10.1007/s11033-014-3570-2 (2014).
92. Kondo, K. *et al.* H2S protects against pressure overload-induced heart failure via upregulation of endothelial nitric oxide synthase. *Circulation* **127**, 1116-1127, doi:10.1161/CIRCULATIONAHA.112.000855 (2013).
93. Lan, A. *et al.* Inhibition of ROS-activated p38MAPK pathway is involved in the protective effect of H2S against chemical hypoxia-induced inflammation in PC12 cells. *Neurochem Res* **38**, 1454-1466, doi:http://dx.doi.org/10.1007/s11064-013-1044-x (2013).
94. Li, H. *et al.* Hydrogen sulfide decreases blood-brain barrier damage via regulating protein kinase C and tight junction after cardiac arrest in rats. *Cell Physiol Biochem* **47**, 994-1006,

doi:http://dx.doi.org/10.1159/000490166 (2018).
95. Li, L., Jiang, H. K., Li, Y. P. & Guo, Y. P. Hydrogen sulfide protects spinal cord and induces autophagy via miR-30c in a rat model of spinal cord ischemia-reperfusion injury. *J Biomed Sci* **22**, 135, doi:http://dx.doi.org/10.1186/s12929-015-0135-1 (2015).
96. Li, T. *et al.* Neuroprotective Effects of Hydrogen Sulfide Against Early Brain Injury and Secondary Cognitive Deficits Following Subarachnoid Hemorrhage. *Brain Pathol* **27**, 51-63, doi:http://dx.doi.org/10.1111/bpa.12361 (2017).
97. Li, X. J. *et al.* Hydrogen sulfide intervention in focal cerebral ischemia/reperfusion injury in rats. *Neural Regen Res* **10**, 932-937, doi:http://dx.doi.org/10.4103/1673-5374.158353 (2015).
98. Li, X. N. *et al.* Hydrogen sulfide attenuates chronic restrain stress-induced cognitive impairment by upreglulation of Sirt1 in hippocampus. *Oncotarget* **8**, 100396-100410, doi:http://dx.doi.org/10.18632/oncotarget.22237 (2017).
99. Ling, K. *et al.* H2S attenuates oxidative stress via Nrf2/NF-κB signaling to regulate restenosis after percutaneous transluminal angioplasty. *Exp Biol Med (Maywood)* **246**, 226-239, doi: 10.1177/1535370220961038 (2021).
100. Liu, C., Xu, X., Gao, J., Zhang, T. & Yang, Z. Hydrogen Sulfide Prevents Synaptic Plasticity from VD-Induced Damage via Akt/GSK-3beta Pathway and Notch Signaling Pathway in Rats. *Mol Neurobiol* **53**, 4159-4172, doi:http://dx.doi.org/10.1007/s12035-015-9324-x (2016).
101. Liu, Y. *et al.* Exogenous H2S Protects Colon Cells in Ulcerative Colitis by Inhibiting NLRP3 and Activating Autophagy. *DNA Cell Biol* **40**, 748-756, doi: 10.1089/dna.2020.6380 (2021).
102. Liu, Y. Y., Sparatore, A., Del Soldato, P. & Bian, J. S. ACS84, a novel hydrogen sulfide-releasing compound, protects against amyloid beta-induced cell cytotoxicity. *Neurochem Int* **58**, 591-598, doi:http://dx.doi.org/10.1016/j.neuint.2011.01.023 (2011).
103. Lu, M., Hu, L. F., Hu, G. & Bian, J. S. Hydrogen sulfide protects astrocytes against H2O2-induced neural injury via enhancing glutamate uptake. *Free Radic Biol Med* **45**, 1705-1713, doi:http://dx.doi.org/10.1016/j.freeradbiomed.2008.09.014 (2008).
104. Lu, M. *et al.* The neuroprotection of hydrogen sulfide against MPTP-induced dopaminergic neuron degeneration involves uncoupling protein 2 rather than ATP-sensitive potassium channels. *Antioxid Redox Signal* **17**, 849-859, doi:http://dx.doi.org/10.1089/ars.2011.4507 (2012).
105. Minaei, A., Sarookhani, M. R., Haghdoost-Yazdi, H. & Rajaei, F. Hydrogen sulfide attenuates induction and prevents progress of the 6-hydroxydopamine-induced Parkinsonism in rat through activation of ATP-sensitive potassium channels and suppression of ER stress. *Toxicol Appl Pharmacol* **423**, 115558, doi: 10.1016/j.taap.2021.115558 (2021).
106. Ni, J. *et al.* Hydrogen sulfide reduces pyroptosis and alleviates ischemia-reperfusion acute kidney injury by inhibiting NLRP3 inflammasome. *Life Sci* 119466, doi:10.1016/j.lfs.2021.119466 (2021).
107. Pan, J. G. *et al.* Protective effect of hydrogen sulfide on hypoxic respiratory suppression in medullary slice of neonatal rats. *Respir Physiol Neurobiol* **171**, 181-186, doi:http://dx.doi.org/10.1016/j.resp.2010.04.006 (2010).
108. Rafaiee, R. *et al.* Hydrogen sulfide protects hippocampal CA1 neurons against lead mediated neuronal damage via reduction oxidative stress in male rats. *J Chem Neuroanat* **112**, 101917, doi: 10.1016/j.jchemneu.2020.101917 (2021).
109. Shan, H. *et al.* Exogenous Hydrogen Sulfide Offers Neuroprotection on Intracerebral Hemorrhage Injury Through Modulating Endogenous H2S Metabolism in Mice. *Front Cell Neurosci* **13**, 349, doi:http://dx.doi.org/10.3389/fncel.2019.00349 (2019).
110. Shao, J. L. *et al.* H2S protects hippocampal neurons from anoxia-reoxygenation through cAMP-mediated PI3K/Akt/p70S6K cell-survival signaling pathways. *J Mol Neurosci* **43**, 453-460, doi:http://dx.doi.org/10.1007/s12031-010-9464-4 (2011).
111. Song, Y. J. *et al.* H2S attenuates injury after ischemic stroke by diminishing the assembly of CaMKII with ASK1-MKK3-p38 signaling module. *Behav Brain Res* **384**, 112520, doi:http://dx.doi.org/10.1016/j.bbr.2020.112520 (2020).

112. Sun, J. *et al.* Neuroprotective effect of hydrogen sulfide against glutamate-induced oxidative stress is mediated via the p53/glutaminase 2 pathway after traumatic brain injury. *Aging* **13**, 7180-7189, doi: 10.18632/aging.202575 (2021).
113. Sun, X. *et al.* Donor heart preservation with a novel long-term and slow-releasing hydrogen sulfide system. *Nitric Oxide Biol Chem* **81**, 1-10, doi:10.1016/j.niox.2018.09.001 (2018).
114. Tang, X. Q. *et al.* Involvement of KATP/PI3K/AKT/Bcl-2 Pathway in Hydrogen Sulfide-induced Neuroprotection Against the Toxicity of 1-methy-4-phenylpyridinium Ion. *J Mol Neurosci* **46**, 442-449, doi:http://dx.doi.org/10.1007/s12031-011-9608-1 (2011).
115. Tang, Y. Y. *et al.* Role of silent information regulator 1 in the protective effect of hydrogen sulfide on homocysteine-induced cognitive dysfunction: Involving reduction of hippocampal ER stress. *Behav Brain Res* **342**, 35-42, doi:http://dx.doi.org/10.1016/j.bbr.2017.12.040 (2018).
116. Tay, A. S., Hu, L. F., Lu, M., Wong, P. T. H. & Bian, J. S. Hydrogen sulfide protects neurons against hypoxic injury via stimulation of ATP-sensitive potassium channel/protein kinase C/extracellular signal-regulated kinase/heat shock protein90 pathway. *Neuroscience* **167**, 277-286, doi:http://dx.doi.org/10.1016/j.neuroscience.2010.02.006 (2010).
117. Vandini, E. *et al.* Mechanisms of hydrogen sulfide against the progression of severe Alzheimer's disease in transgenic mice at different ages. *Pharmacology* **103**, 93-100, doi:10.1159/000494113 (2019).
118. Wang, B. *et al.* Hydrogen sulfide protects against IL-1β-induced inflammation and mitochondrial dysfunction-related apoptosis in chondrocytes and ameliorates osteoarthritis. *J Cell Physiol* **236**, 4369-4386, doi: 10.1002/jcp.30154 (2021).
119. Wang, M. *et al.* Hydrogen sulfide enhances adult neurogenesis in a mouse model of Parkinson's disease. *Neural Regen Res* **16**, 1353-1358, doi: 10.4103/1673-5374.301026 (2021).
120. Wang, Y. *et al.* Hydrogen sulfide protects blood-brain barrier integrity following cerebral ischemia. *J Neurochem* **129**, 827-838, doi:http://dx.doi.org/10.1111/jnc.12695 (2014).
121. Wei, H. J. *et al.* Hydrogen sulfide inhibits homocysteine-induced endoplasmic reticulum stress and neuronal apoptosis in rat hippocampus via upregulation of the BDNF-TrkB pathway. *Acta Pharmacol Sin* **35**, 707-715, doi:http://dx.doi.org/10.1038/aps.2013.197 (2014).
122. Wei, X. *et al.* Hydrogen sulfide induces neuroprotection against experimental stroke in rats by down-regulation of AQP4 via activating PKC. *Brain Res* **1622**, 292-299, doi:http://dx.doi.org/10.1016/j.brainres.2015.07.001 (2015).
123. Wei, X. *et al.* Hydrogen Sulfide Inhalation Improves Neurological Outcome via NF-kappaB-Mediated Inflammatory Pathway in a Rat Model of Cardiac Arrest and Resuscitation. *Cell Physiol Biochem* **36**, 1527-1538, doi:http://dx.doi.org/10.1159/000430316 (2015).
124. Wen, J.-Y. *et al.* Endothelium-derived hydrogen sulfide acts as a hyperpolarizing factor and exerts neuroprotective effects via activation of large-conductance Ca^{2+} -activated K^+ channels. *Br J Pharmacol* doi:10.1111/bph.15607 (2021).
125. Xiao, Q. *et al.* Exogenous hydrogen sulfide inhibits human melanoma cell development via suppression of the PI3K/AKT/ mTOR pathway. *J Dermatol Sci* **98**, 26-34, doi: 10.1016/j.jdermsci.2020.02.004 (2020).
126. Xiong, Q. *et al.* Hydrogen sulfide stabilizes atherosclerotic plaques in apolipoprotein E knockout mice. *Pharmacol Res* **144**, 90-98, doi:10.1016/j.phrs.2019.04.006 (2019).
127. Xu, X., Liu, C., Li, Z. & Zhang, T. Effects of Hydrogen Sulfide on Modulation of Theta-Gamma Coupling in Hippocampus in Vascular Dementia Rats. *Brain Topogr* **28**, 879-894, doi:http://dx.doi.org/10.1007/s10548-015-0430-x (2015).
128. Yang, B. *et al.* Activation of autophagic flux and the Nrf2/ARE signaling pathway by hydrogen sulfide protects against acrylonitrile-induced neurotoxicity in primary rat astrocytes. *Arch Toxicol* **92**, 2093-2108, doi:http://dx.doi.org/10.1007/s00204-018-2208-x (2018).
129. Zhao, F. L. *et al.* Hydrogen Sulfide Selectively Inhibits gamma-Secretase Activity and Decreases Mitochondrial Abeta Production in Neurons from APP/PS1 Transgenic Mice. *Neurochem Res* **41**, 1145-1159, doi:http://dx.doi.org/10.1007/s11064-015-1807-7 (2016).

Nitric Oxide (NO)

130. Colasanti, M., Mazzone, V., Mancinelli, L., Leone, S. & Venturini, G. Involvement of nitric oxide in the head regeneration of Hydra vulgaris. *Nitric Oxide* **21**, 164-170, doi:https://dx.doi.org/10.1016/j.niox.2009.07.003 (2009).
131. Declèves, A. É. *et al.* Protective effect of nitric oxide in aristolochic acid-induced toxic acute kidney injury: an old friend with new assets. *Exp Physiol* **101**, 193-206, doi:10.1113/EP085333 (2016).
132. Dezfulian, C. *et al.* Mechanistic characterization of nitrite-mediated neuroprotection after experimental cardiac arrest. *J Neurochem* **139**, 419-431, doi:https://dx.doi.org/10.1111/jnc.13764 (2016).
133. Diao, T. J. *et al.* Protective effect of nitric oxide on hepatopulmonary syndrome from ischemia-reperfusion injury. *World J Gastroenterol* **18**, 3310-3316, doi:10.3748/wjg.v18.i25.3310 (2012).
134. Gomes de Almeida Schirmer, B. *et al.* The NO-donor MPC-1011 stimulates angiogenesis and arteriogenesis and improves hindlimb ischemia via a cGMP-dependent pathway involving VEGF and SDF-1alpha. *Atherosclerosis* **304**, 30-38, doi:http://dx.doi.org/10.1016/j.atherosclerosis.2020.05.012 (2020).
135. Jiang, H. *et al.* Dietary nitrite improves insulin signaling through GLUT4 translocation. *Free Radic Biol Med* **67**, 51-57, doi:10.1016/j.freeradbiomed.2013.10.809 (2014).
136. Kajimoto, M. *et al.* Inhaled nitric oxide reduces injury and microglia activation in porcine hippocampus after deep hypothermic circulatory arrest. *J Thorac Cardiovasc Surg* **161**, e485-e498, doi: 10.1016/j.jtcvs.2019.12.075 (2021).
137. Lee, J., Yang, C., Ahn, S., Choi, Y. & Lee, K. Enhanced NO-induced angiogenesis via NO/H2S co-delivery from self-assembled nanoparticles. *Biomater Sci* **9**, 5150-5159, doi: 10.1039/d1bm00448d (2021).
138. Lin, Y. F., Raab-Graham, K., Jan, Y. N. & Jan, L. Y. NO stimulation of ATP-sensitive potassium channels: Involvement of Ras/mitogen-activated protein kinase pathway and contribution to neuroprotection. *Proc Natl Acad Sci U S A* **101**, 7799-7804, doi:http://dx.doi.org/10.1073/pnas.0402496101 (2004).
139. Pansiot, J. *et al.* Neuroprotective effect of inhaled nitric oxide on excitotoxic-induced brain damage in neonatal rat. *PLoS ONE* **5**, e10916, doi:http://dx.doi.org/10.1371/journal.pone.0010916 (2010).
140. Santos, A. I. *et al.* S-Nitrosylation of Ras Mediates Nitric Oxide-Dependent Post-Injury Neurogenesis in a Seizure Model. *Antioxid Redox Signal* **28**, 15-30, doi:http://dx.doi.org/10.1089/ars.2016.6858 (2018).
141. Schroter, A., Andrabi, S. A., Wolf, G. & Horn, T. F. W. Nitric oxide applications prior and simultaneous to potentially excitotoxic NMDA-evoked calcium transients: Cell death or survival. *Brain Res* **1060**, 1-15, doi:http://dx.doi.org/10.1016/j.brainres.2005.07.065 (2005).
142. Serghides, L. *et al.* Inhaled nitric oxide reduces endothelial activation and parasite accumulation in the brain, and enhances survival in experimental cerebral malaria. *PLoS ONE* **6**, e27714, doi:http://dx.doi.org/10.1371/journal.pone.0027714 (2011).
143. Stern, M. & Bicker, G. Nitric oxide regulates axonal regeneration in an insect embryonic CNS. *Develop Neurobiol* **68**, 295-308, doi:https://doi.org/10.1002/dneu.20585 (2008).
144. Sugimoto, R. *et al.* Nitrite reduces acute lung injury and improves survival in a rat lung transplantation model. *Am J Transplant* **12**, 2938-2948, doi:10.1111/j.1600-6143.2012.04169.x (2012).
145. Sun, J. *et al.* Essential role of nitric oxide in acute ischemic preconditioning: S-nitros(yl)ation versus sGC/cGMP/PKG signaling? *Free Radic Biol Med* **54**, 105-112, doi:10.1016/j.freeradbiomed.2012.09.005 (2013).
146. Wang, L., Li, M. & Zhou, L. Nitric oxide promotes survival of cerebellar granule neurons cultured in vitro through the Akt pathway. *Neural Regen Res* **6**, 1559-1563, doi:http://dx.doi.org/10.3969/j.issn.1673-5374.2011.20.007 (2011).
147. Zhan, R. *et al.* Nitric oxide promotes epidermal stem cell proliferation via FOXG1-c-Myc signalling. *Nitric Oxide Biol Chem* **73**, 1-8, doi:10.1016/j.niox.2017.12.002 (2018).

Oxygen (O_2)

148. Chen, C. H., Cui, H. M., Li, Z. H., Wang, R. F. & Zhou, C. M. Normobaric oxygen for cerebral ischemic injury. *Neural Regen Res* **8**, 2885-2894, doi:http://dx.doi.org/10.3969/j.issn.1673-5374.2013.31.001 (2013).
149. Fang, Z. *et al.* Neuroprotective Autophagic Flux Induced by Hyperbaric Oxygen Preconditioning is Mediated by Cystatin C. *Neurosci Bull* **35**, 336-346, doi:https://dx.doi.org/10.1007/s12264-018-0313-8 (2019).
150. Feng, Y. *et al.* Hyperbaric oxygen preconditioning protects lung against hyperoxic acute lung injury in rats via heme oxygenase-1 induction. *Biochem Biophys Res Commun* **456**, 549-554, doi:10.1016/j.bbrc.2014.09.074 (2015).
151. He, X. *et al.* Preconditioning with hyperbaric oxygen induces tolerance against renal ischemia-reperfusion injury via increased expression of heme oxygenase-1. *J Surg Res* **170**, e271-277, doi:10.1016/j.jss.2011.06.008 (2011).
152. Ho, Y. J. *et al.* Normalization of tumor vasculature by oxygen microbubbles with ultrasound. *Theranostics* **9**, 7370-7383, doi:10.7150/thno.37750 (2019).
153. Horie, M. *et al.* Enhancement of satellite cell differentiation and functional recovery in injured skeletal muscle by hyperbaric oxygen treatment. *J Appl Physiol* **116**, 149-155, doi:https://dx.doi.org/10.1152/japplphysiol.00235.2013 (2014).
154. Hou, Y. N. *et al.* Effect of hyperbaric oxygen on MMP9/2 expression and motor function in rats with spinal cord injury. *Int J Clin Exp Med* **8**, 14926-14934 (2015).
155. Huang, G. *et al.* Signaling pathways involved in HSP32 induction by hyperbaric oxygen in rat spinal neurons. *Redox Biol* **10**, 108-118, doi:10.1016/j.redox.2016.09.011 (2016).
156. Lee, C. C. *et al.* Hyperbaric oxygen induces VEGF expression through ERK, JNK and c-Jun/AP-1 activation in human umbilical vein endothelial cells. *J Biomed Sci* **13**, 143-156, doi:10.1007/s11373-005-9037-7 (2006).
157. Lin, K. C. *et al.* Combined therapy with hyperbaric oxygen and melatonin effectively reduce brain infarct volume and preserve neurological function after acute ischemic infarct in rat. *J Neuropathol Exp Neurol* **78**, 949-960, doi:10.1093/jnen/nlz076 (2019).
158. Lin, S. S. *et al.* Hyperbaric oxygen inhibits the HMGB1/RAGE signaling pathway by upregulating Mir-107 expression in human osteoarthritic chondrocytes. *Osteoarthritis Cartilage* **27**, 1372-1381, doi:10.1016/j.joca.2019.05.011 (2019).
159. Liu, X. *et al.* Effect of VEGF and CX43 on the promotion of neurological recovery by hyperbaric oxygen treatment in spinal cord-injured rats. *Spine J* **14**, 119-127, doi:10.1016/j.spinee.2013.06.084 (2014).
160. Majmundar, A. J. *et al.* O 2 regulates skeletal muscle progenitor differentiation through phosphatidylinositol 3-kinase/AKT signaling. *Mol Cell Biol* **32**, 36-49, doi:10.1128/MCB.05857-11 (2012).
161. Matsuoka, H. *et al.* Administration of Oxygen Ultra-Fine Bubbles Improves Nerve Dysfunction in a Rat Sciatic Nerve Crush Injury Model. *Int J Mol Sci* **19**, 1395, doi:https://dx.doi.org/10.3390/ijms19051395 (2018).
162. Müller, A. *et al.* The effect of hyperbaric oxygen therapy on erectile function recovery in a rat cavernous nerve injury model. *J Sex Med* **5**, 562-570, doi:10.1111/j.1743-6109.2007.00727.x (2008).
163. Oyaizu, T. *et al.* Hyperbaric oxygen reduces inflammation, oxygenates injured muscle, and regenerates skeletal muscle via macrophage and satellite cell activation. *Sci Rep* **8**, 1288, doi:https://dx.doi.org/10.1038/s41598-018-19670-x (2018).
164. Ozden, T. A. *et al.* The effects of hyperbaric oxygen treatment on oxidant and antioxidants levels during liver regeneration in rats. *Tohoku J Exp Med* **203**, 253-265, doi:http://dx.doi.org/10.1620/tjem.203.253 (2004).
165. Palzur, E., Zaaroor, M., Vlodavsky, E., Milman, F. & Soustiel, J. F. Neuroprotective effect of

hyperbaric oxygen therapy in brain injury is mediated by preservation of mitochondrial membrane properties. *Brain Res* **1221**, 126-133, doi:10.1016/j.brainres.2008.04.078 (2008).
166. Tian, X. *et al.* The protective effect of hyperbaric oxygen and Ginkgo biloba extract on Abeta25-35-induced oxidative stress and neuronal apoptosis in rats. *Behav Brain Res* **242**, 1-8, doi:http://dx.doi.org/10.1016/j.bbr.2012.12.026 (2013).
167. Wang, M., Geng, X., Dandu, C., Patel, R. & Ding, Y. Normobaric Oxygen (NBO) Therapy Reduces Cerebral Ischemia/Reperfusion Injury through Inhibition of Early Autophagy. *Evid Based Complement Alternat Med* **2021**, 7041290, doi: 10.1155/2021/7041290 (2021).
168. Wang, S.-D. *et al.* Hyperbaric Oxygen Preconditioning Protects Against Cerebral Ischemia/Reperfusion Injury by Inhibiting Mitochondrial Apoptosis and Energy Metabolism Disturbance. *Neurochem Res* **46**, 866-877, doi: 10.1007/s11064-020-03219-4 (2021).
169. Wang, Y., Zhang, S., Luo, M. & Li, Y. Hyperbaric oxygen therapy improves local microenvironment after spinal cord injury. *Neural Regen Res* **9**, 2182-2188, doi:10.4103/1673-5374.147951 (2014).
170. Yamamoto, N. *et al.* VEGF and bFGF induction by nitric oxide is associated with hyperbaric oxygen-induced angiogenesis and muscle regeneration. *Sci Rep* **10**, 2744, doi:https://dx.doi.org/10.1038/s41598-020-59615-x (2020).
171. Yin, X. *et al.* Hyperbaric Oxygen Preconditioning Attenuates Myocardium Ischemia-Reperfusion Injury Through Upregulation of Heme Oxygenase 1 Expression: PI3K/Akt/Nrf2 Pathway Involved. *J Cardiovasc Pharmacol Ther* **20**, 428-438, doi:10.1177/1074248414568196 (2015).
172. Zhou, Y. *et al.* Hyperbaric oxygen intervention on expression of hypoxia-inducible factor-1α and vascular endothelial growth factor in spinal cord injury models in rats. *Chin Med J* **126**, 3897-3903, doi:10.3760/cma.j.issn.0366-6999.20130571 (2013).

Ozone (O_3)

173. Cisterna, B. *et al.* Ozone activates the NRF2 pathway and improves preservation of explanted adipose tissue in vitro. *Antioxidants* **9**, 1-15, doi:10.3390/antiox9100989 (2020).
174. Hao, K. *et al.* Ozone promotes regeneration by regulating the inflammatory response in zebrafish. *Int Immunopharmacol* **28**, 369-375, doi:https://dx.doi.org/10.1016/j.intimp.2015.05.026 (2015).
175. Morsy, M. D., Hassan, W. N. & Zalat, S. I. Improvement of renal oxidative stress markers after ozone administration in diabetic nephropathy in rats. *Diabetol Metab Syndr* **2**, 29, doi:10.1186/1758-5996-2-29 (2010).
176. Oakes, J. L. *et al.* Ozone enhances pulmonary innate immune response to a toll-like receptor-2 agonist. *Am J Resp Cell Mol Biol* **48**, 27-34, doi:10.1165/rcmb.2012-0187OC (2013).
177. Orakdogen, M. *et al.* The Effect of Ozone Therapy on Experimental Vasospasm in the Rat Femoral Artery. *Turk Neurosurg* **26**, 860-865, doi:10.5137/1019-5149.Jtn.14162-15.2 (2016).
178. Qiu, T. *et al.* Effect of ozone oxidative preconditioning on oxidative stress injury in a rat model of kidney transplantation. *Exp Ther Med* **13**, 1948-1955, doi:10.3892/etm.2017.4193 (2017).
179. Soares, C. D. *et al.* Effects of subcutaneous injection of ozone during wound healing in rats. *Growth Factors* **37**, 95-103, doi:10.1080/08977194.2019.1643339 (2019).
180. Xiao, W. *et al.* Ozone oil promotes wound healing by increasing the migration of fibroblasts via PI3K/Akt/mTOR signaling pathway. *Biosci Rep* **37**, BSR20170658, doi:10.1042/BSR20170658 (2017).

Noble Gases

Argon (Ar)

181. Abraini, J. H., Kriem, B., Balon, N., Rostain, J. C. & Risso, J. J. Gamma-aminobutyric acid neuropharmacological investigations on narcosis produced by nitrogen, argon, or nitrous oxide. *Anesth Analg* **96**, 746-749, doi:10.1213/01.ane.0000050282.14291.38 (2003).

182. David, H. N. *et al.* Ex vivo and in vivo neuroprotection induced by argon when given after an excitotoxic or ischemic insult. *PLoS ONE* **7**, e30934, doi:http://dx.doi.org/10.1371/journal.pone.0030934 (2012).
183. Fahlenkamp, A. V. *et al.* The noble gas argon modifies extracellular signal-regulated kinase 1/2 signaling in neurons and glial cells. *Eur J Pharmacol* **674**, 104-111, doi:10.1016/j.ejphar.2011.10.045 (2012).
184. Faure, A. *et al.* Effectiveness of pure argon for renal transplant preservation in a preclinical pig model of heterotopic autotransplantation. *J Transl Med* **14**, 40, doi:10.1186/s12967-016-0795-y (2016).
185. Goebel, U. *et al.* Argon reduces microglial activation and inflammatory cytokine expression in retinal ischemia/reperfusion injury. *Neural Regen Res* **16**, 192-198, doi:http://dx.doi.org/10.4103/1673-5374.290098 (2021).
186. Qi, H. *et al.* Pretreatment With Argon Protects Human Cardiac Myocyte-Like Progenitor Cells from Oxygen Glucose Deprivation-Induced Cell Death by Activation of AKT and Differential Regulation of Mapkinases. *Shock* **49**, 556-563, doi:10.1097/shk.0000000000000998 (2018).
187. de Roux, Q. *et al.* Argon Attenuates Multiorgan Failure in Relation with HMGB1 Inhibition. *Int J Mol Sci* **22**, 3257, doi:10.3390/ijms22063257 (2021).
188. Ulbrich, F. *et al.* Argon Mediates Anti-Apoptotic Signaling and Neuroprotection via Inhibition of Toll-Like Receptor 2 and 4. *PLoS ONE* **10**, e0143887, doi:https://dx.doi.org/10.1371/journal.pone.0143887 (2015).
189. Ulbrich, F. *et al.* Neuroprotective effects of Argon are mediated via an ERK-1/2 dependent regulation of heme-oxygenase-1 in retinal ganglion cells. *J Neurochem* **134**, 717-727, doi:10.1111/jnc.13115 (2015).
190. Ulbrich, F. *et al.* Argon mediates protection by interleukin-8 suppression via a TLR2/TLR4/STAT3/NF-κB pathway in a model of apoptosis in neuroblastoma cells in vitro and following ischemia-reperfusion injury in rat retina in vivo. *J Neurochem* **138**, 859-873, doi:10.1111/jnc.13662 (2016).
191. Zhuang, L. *et al.* The protective profile of argon, helium, and xenon in a model of neonatal asphyxia in rats. *Crit Care Med* **40**, 1724-1730, doi:10.1097/CCM.0b013e3182452164 (2012).

Helium (He)

192. Aehling, C. *et al.* Effects of combined helium pre/post-conditioning on the brain and heart in a rat resuscitation model. *Acta Anaesthesiol Scand* **62**, 63-74, doi:10.1111/aas.13041 (2018).
193. Flick, M. *et al.* Helium postconditioning regulates expression of caveolin-1 and -3 and induces RISK pathway activation after ischaemia/reperfusion in cardiac tissue of rats. *Eur J Pharmacol* **791**, 718-725, doi:10.1016/j.ejphar.2016.10.012 (2016).
194. Li, Y., Zhang, P., Liu, Y., Liu, W. & Yin, N. Helium preconditioning protects the brain against hypoxia/ischemia injury via improving the neurovascular niche in a neonatal rat model. *Behav Brain Res* **314**, 165-172, doi:10.1016/j.bbr.2016.08.015 (2016).
195. Liu, Y. *et al.* Helium preconditioning attenuates hypoxia/ischemia-induced injury in the developing brain. *Brain Res* **1376**, 122-129, doi:10.1016/j.brainres.2010.12.068 (2011).
196. Pagel, P. S. *et al.* Inhibition of glycogen synthase kinase or the apoptotic protein p53 lowers the threshold of helium cardioprotection in vivo: the role of mitochondrial permeability transition. *Anesth Analg* **107**, 769-775, doi:10.1213/ane.0b013e3181815b84 (2008).
197. Weber, N. C. *et al.* Helium-Induced Changes in Circulating Caveolin in Mice Suggest a Novel Mechanism of Cardiac Protection. *Int J Mol Sci* **20**, 2640, doi:10.3390/ijms20112640 (2019).

Xenon (Xe)

198. Filev, A. D. *et al.* Effect of Xenon Treatment on Gene Expression in Brain Tissue after Traumatic

Brain Injury in Rats. *Brain Sci* **11**, 889, doi: 10.3390/brainsci11070889 (2021).
199. Harris, K. *et al.* Neuroprotection against traumatic brain injury by xenon, but not argon, is mediated by inhibition at the n-methyl-d-aspartate receptor glycine site. *Anesthesiology* **119**, 1137-1148, doi:http://dx.doi.org/10.1097/ALN.0b013e3182a2a265 (2013).
200. Koziakova, M. *et al.* Noble gas neuroprotection: xenon and argon protect against hypoxic-ischaemic injury in rat hippocampus in vitro via distinct mechanisms. *Br J Anaesth* **123**, 601-609, doi:10.1016/j.bja.2019.07.010 (2019).
201. Lavaur, J. *et al.* The noble gas xenon provides protection and trophic stimulation to midbrain dopamine neurons. *J Neurochem* **142**, 14-28, doi:http://dx.doi.org/10.1111/jnc.14041 (2017).
202. Li, Q. *et al.* Pretreatment With Xenon Protected Immature Rabbit Heart From Ischaemia/Reperfusion Injury by Opening of the mitoKATP Channel. *Heart Lung Circul* **22**, 276-283, doi:10.1016/j.hlc.2012.10.016 (2013).
203. Limatola, V. *et al.* Xenon preconditioning confers neuroprotection regardless of gender in a mouse model of transient middle cerebral artery occlusion. *Neuroscience* **165**, 874-881, doi:http://dx.doi.org/10.1016/j.neuroscience.2009.10.063 (2010).
204. Liu, S. *et al.* Xenon-delayed postconditioning attenuates spinal cord ischemia/reperfusion injury through activation AKT and ERK signaling pathways in rats. *J Neurol Sci* **368**, 277-284, doi:10.1016/j.jns.2016.07.009 (2016).
205. Luo, Y. *et al.* Xenon and sevoflurane protect against brain injury in a neonatal asphyxia model. *Anesthesiology* **109**, 782-789, doi:10.1097/ALN.0b013e3181895f88 (2008).
206. Ma, D. *et al.* Xenon and hypothermia combine to provide neuroprotection from neonatal asphyxia. *Ann Neurol* **58**, 182-193, doi:http://dx.doi.org/10.1002/ana.20547 (2005).
207. Ma, D. *et al.* Xenon preconditioning protects against renal ischemic-reperfusion injury via HIF-1alpha activation. *J Am Soc Nephrol* **20**, 713-720, doi:10.1681/asn.2008070712 (2009).
208. Peng, T. *et al.* Therapeutic time window and dose dependence of xenon delivered via echogenic liposomes for neuroprotection in stroke. *CNS Neurosci Ther* **19**, 773-784, doi:http://dx.doi.org/10.1111/cns.12159 (2013).
209. Petzelt, C., Blom, P., Schmehl, W., Muller, J. & Kox, W. J. Prevention of neurotoxicity in hypoxic cortical neurons by the noble gas xenon. *Life Sci* **72**, 1909-1918, doi:http://dx.doi.org/10.1016/S0024-3205%2802%2902439-6 (2003).
210. Petzelt, C., Blom, P., Schmehl, W., Müller, J. & Kox, W. J. Xenon prevents cellular damage in differentiated PC-12 cells exposed to hypoxia. *BMC Neurosci* **5**, 55, doi:10.1186/1471-2202-5-55 (2004).
211. Rizvi, M. *et al.* Effect of noble gases on oxygen and glucose deprived injury in human tubular kidney cells. *Exp Biol Med* **235**, 886-891, doi:10.1258/ebm.2010.009366 (2010).
212. Yang, Y. W. *et al.* Timing of xenon-induced delayed postconditioning to protect against spinal cord ischaemia-reperfusion injury in rats. *Br J Anaesth* **113**, 168-176, doi:10.1093/bja/aet352 (2014).

Reports on Findings of Adverse Effects or No Significant Therapeutic Impact of Medical Gases on Some or All Outcome Measures

- Biological Gases

Carbon Monoxide (CO)

213. Cheng, Y. *et al.* Neurodevelopmental consequences of sub-clinical carbon monoxide exposure in newborn mice. *PLoS ONE* **7**, e32029, doi:10.1371/journal.pone.0032029 (2012).
214. Lopez, I., Acuna, D., Webber, D. S., Korsak, R. A. & Edmond, J. Mild carbon monoxide exposure diminishes selectively the integrity of the cochlea of the developing rat. *J Neurosci Res* **74**, 666-675, doi:10.1002/jnr.10813 (2003).

Hydrogen (H_2)

215. Matchett, G. A. *et al.* Hydrogen gas is ineffective in moderate and severe neonatal hypoxia-ischemia rat models. *Brain Res* **1259**, 90-97, doi:10.1016/j.brainres.2008.12.066 (2009).
216. Takeuchi, S., Nagatani, K., Otani, N., Wada, K. & Mori, K. Hydrogen does not exert neuroprotective effects or improve functional outcomes in rats after intracerebral hemorrhage. *Turk Neurosurg* **26**, 854-859, doi:10.5137/1019-5149.Jtn.14098-15.1 (2016).

Hydrogen Sulfide (H_2S)

217. Cheung, N. S., Peng, Z. F., Chen, M. J., Moore, P. K. & Whiteman, M. Hydrogen sulfide induced neuronal death occurs via glutamate receptor and is associated with calpain activation and lysosomal rupture in mouse primary cortical neurons. *Neuropharmacology* **53**, 505-514, doi:10.1016/j.neuropharm.2007.06.014 (2007).
218. Kim, D. S. *et al.* Transcriptomic profile analysis of brain inferior colliculus following acute hydrogen sulfide exposure. *Toxicology* **430**, 152345, doi:10.1016/j.tox.2019.152345 (2020).

Oxygen (O_2)

219. Badr, A. E., Yin, W., Mychaskiw, G. & Zhang, J. H. Dual effect of HBO on cerebral infarction in MCAO rats. *Am J Physiol Regul Integr Comp Physiol* **280**, R766-770, doi:10.1152/ajpregu.2001.280.3.R766 (2001).
220. Hjelde, A., Hjelstuen, M., Haraldseth, O., Martin, D., Thom, R., Brubakk, O. Hyperbaric oxygen and neutrophil accumulation/tissue damage during permanent focal cerebral ischaemia in rats. *Eur J Appl Physiol* **86**, 401-405. doi: 10.1007/s00421-001-0573-1 (2002).
221. Veltkamp, R. *et al.* Oxygen therapy in permanent brain ischemia: potential and limitations. *Brain Res* **1107**, 185-191, doi:10.1016/j.brainres.2006.05.108 (2006).

- Noble Gases:

Argon (Ar)

199. Harris, K. et al. Neuroprotection against traumatic brain injury by xenon, but not argon, is mediated by inhibition at the n-methyl-d-aspartate receptor glycine site. *Anesthesiology* **119**, 1137-1148, doi:http://dx.doi.org/10.1097/ALN.0b013e3182a2a265 (2013).
222. David, H. N. *et al.* Ex vivo and in vivo neuroprotection induced by argon when given after an excitotoxic or ischemic insult. *PLoS ONE* **7**, e30934, doi:10.1371/journal.pone.0030934 (2012).

Helium (He)

191. Zhuang, L. *et al.* The protective profile of argon, helium, and xenon in a model of neonatal asphyxia in rats. *Crit Care Med* **40**, 1724-1730, doi:10.1097/CCM.0b013e3182452164 (2012)
192. Aehling, C. et al. Effects of combined helium pre/post-conditioning on the brain and heart in a rat resuscitation model. *Acta Anaesthesiol Scand* **62**, 63-74, doi:10.1111/aas.13041 (2018).
211. Rizvi, M. et al. Effect of noble gases on oxygen and glucose deprived injury in human tubular kidney cells. *Exp Biol Med* **235**, 886-891, doi:10.1258/ebm.2010.009366 (2010).

Xenon (Xe)

223. Brosnan, H. & Bickler, P. E. Xenon neurotoxicity in rat hippocampal slice cultures is similar to isoflurane and sevoflurane. *Anesthesiology* **119**, 335-344, doi:10.1097/ALN.0b013e31829417f0 (2013).

Section S3: Supporting Information

Abbreviations and References of Tables 2-4

Table 2. Representative Tissue Protective Effects of Medical Gases

Abbreviations:

AD, Alzheimer's disease; Akt, protein kinase B; AMPKα, adenosine monophosphate-activated protein kinase α subunit; AP-1, activating protein-1; ATP, adenosine triphosphate; Bax, BCL2 Associated X: a protein coding gene regulating apoptosis; Bach1, BTB And CNC Homology 1/basic leucine zipper transcription factor 1; Bcl2, B-cell lymphoma 2: a protein coding gene regulating apoptosis; Cav-1/3, caveolin-1/3; cGMP, cyclic guanosine monophosphate; c-Myc, cellular Myc proto-oncogene (Myc: abbreviation for myelocytoma); CO, carbon monoxide; CORM, carbon monoxide releasing molecule; CREB, cAMP response element-binding protein; eNOS, endothelial nitric oxide synthase; ER, endoplasmic reticulum; ERK1/2, extracellular regulated kinase 1/2; ERRα, estrogen-related receptor alpha; ERS, endoplasmic reticulum stress; FOXG1, Forkhead Box G1: a protein coding gene; Fyn: a proto-oncogene tyrosine-protein kinase that belongs to the Src family kinases; GABA, gamma-aminobutyric acid; GSK, glycogen synthase kinase; GSK3β, glycogen synthase kinase 3β; HIF1α, hypoxia-inducible factor 1-alpha; HMGB1: high mobility group box 1 protein; HMOX1, a human gene encoding for the heme oxygenase 1; HO-1, heme-oxygenase 1; I/R, ischemia/reperfusion; Irf1, interferon regulatory factor 1; IL, interleukin; L-type Ca^{2+} channels, long-lasting calcium channels; M2 macrophages: M2 subtype-polarized macrophages mainly produce Arginase-I, IL-10 and TGF-β and other anti-inflammatory cytokines to promote tissue repair and wound healing; MAPK, mitogen-activated protein kinase; MCAO, middle cerebral artery occlusion; MEK, MAP kinase/ERK kinase or mitogen-activated protein kinase kinase; MIP-1α, macrophage inflammatory protein 1; miR, microRNA; MPa, megapascal (1 MPa = 10 bar or ~145 PSI/ pounds per square inch); mPTP, mitochondrial permeability transition pore; mTOR, the mammalian target of rapamycin: a kinase; NF-κB, nuclear factor kappa-light-chain-enhancer of activated B cells; NMDA receptor, N -methyl-D-aspartate receptor; nNOS, neuronal nitric oxide synthase; NO, nitric oxide; Nrf2, the nuclear factor erythroid 2–related factor 2; NSCs, neural stem cells; OGD, oxygen-glucose deprivation; PD, Parkinson's disease; PDE5A, phosphodiesterase-5A; PGC1α, peroxisome proliferator-activated receptor gamma coactivator 1α; PI3K, phosphoinositide 3-kinase; PINK1, PTEN-induced kinase 1 that is a mitochondrial serine/threonine-protein kinase; PKG, cyclic GMP–dependent protein kinase; p-p38MAPK, phosphorylated p38 mitogen-activated protein kinases; Ras, Ras (rat sarcoma) proteins belonging to small GTPase family; RISK, the reperfusion injury salvage kinase; ROS, reactive oxygen species; SAH, subarachnoid hemorrhage; SCI, spinal cord injury; sGC, soluble guanylyl cyclase; SOD, superoxide dismutase; STAT3, signal transducer and activator of transcription 3; STZ, streptozotocin; TBI, traumatic brain injury; TLR, the toll-like receptor family; VEGF, vascular endothelial growth factor; and Xe-ELIP, xenon containing echogenic liposomes.

Citations: (note: these references were also cited in **Tables S2-S4**)

[1] a) K. M. Kim, H. O. Pae, M. Zheng, R. Park, Y. M. Kim, H. T. Chung, Circ Res 2007, 101, 919; b) J. Bai, Y. Bai, X.-P. Wang, W.-C. Zheng, L.-M Zhang, Shock 2021, 55, 816.

[2] Y. K. Choi, J. H. Kim, D. K. Lee, K. S. Lee, M. H. Won, D. Jeoung, H. Lee, K. S. Ha, Y. G. Kwon, Y. M. Kim, Antioxid. Redox Signal. 2017, 27, 20.

[3] P. Lakkisto, V. Kytö, H. Forsten, J. M. Siren, H. Segersvärd, L. M. Voipio-Pulkki, M. Laine, K. Pulkki, I. Tikkanen, Eur J Pharmacol 2010, 635, 156.

[4] Y. K. Choi, C. K. Kim, H. Lee, D. Jeoung, K. S. Ha, Y. G. Kwon, K. W. Kim, Y. M. Kim, J Biol Chem 2010, 285, 32116.

[5] a) F. Ulbrich, C. Hagmann, H. Buerkle, C. C. Romao, N. Schallner, U. Goebel, J. Biermann, J Neuroinflammation 2017, 14, 130; b) M. J. Uddin, J. Jeong, E. S. Park, H. Ha, Oxid Med Cell Longev 2021, 9947772; c) B. Song, C. Zhang, W. Hu, C. Guo, Z. Xia, W. Hu, M. Qin, W. Jiang, J. Lv, D. Xu, S. Zhang, J. Fang, J Control Release 2021, 331:350.

[6] Y. K. Choi, T. Maki, E. T. Mandeville, S. H. Koh, K. Hayakawa, K. Arai, Y. M. Kim, M. J. Whalen, C. Xing, X. Wang, K. W. Kim, E. H. Lo, Nat Med 2016, 22, 1335.

[7] a) X. L. He, N. Yan, X. S. Chen, Y. W. Qi, Y. Yan, Z. Cai, Pharmacol. Rep. 2016, 68, 975; b) C. Liu, X. Xu, J. Gao, T. Zhang, Z. Yang, Mol Neurobiol 2016, 53, 4159.

[8] a) S. C. Bir, G. K. Kolluru, P. McCarthy, X. Shen, S. Pardue, C. B. Pattillo, C. G. Kevil, J Am Heart Assoc 2012, 1, e004093; b) D. Giovinazzo, B. Bursac,J. I. Sbodio, S. Nalluru, T. Vignane, A. M. Snowman, L. M. Albacarys, T. W. Sedlak, R. Torregrossa, M. Whiteman, M.R. Filipovic, S. H. Snyder, B. D. Paul,

Proc Natl Acad Sci U S A 2021, 118(4):e2017225118.

[9] a) S. Pardue, G.K. Kolluru, X. Shen, S. E. Lewis, C. B. Saffle, E. E. Kelley, C. G. Kevil.

Redox Biol 2020, 34:101447; b) Q. Hu, X. Ke, T. Zhang, Y. Chen, Q. Huang, B. Deng, S. Xie, J. Wang, R. Nie, J Hypertens 2019, 37, 972.

[10] a) S. Huang, P. Huang, Z. Lin, X. Liu, X. Xu, L. Guo, X. Shen, C. Li, Y. Zhong, Exp Eye Res 2018, 168, 33; b) S. M. Hazzaa, E. S. E. Elsayed Arafat, A. E. Abdo Ismail, A. E. A. Eltorgoman, S. A. Abdelaziz, Y. F. A. Kombr, R. A. Zidan, M. F. Assar, Tissue Cell 2021, 72:101550.

[11] a) X. Jiang, Y. Huang, W. Lin, D. Gao, Z. Fei, J Surg Res 2013, 184, E27; b) A. Minaei, M. R. Sarookhani, H. Haghdoost-Yazdi, F. Rajaei, Toxicol Appl Pharmacol 2021, 423,115558.

[12] a) B. Yang, Y. Bai, C. Yin, H. Qian, G. Xing, S. Wang, F. Li, J. Bian, M. Aschner, R. Lu, Arch Toxicol 2018, 92, 2093; b) L. Chen, K. Ma, H. Fan, X. Wang, T. Cao, Exp Ther Med 2021, 22, 799.

[13] a) R. Zhan, F. Wang, Y. Wu, Y. Wang, W. Qian, M. Liu, T. Liu, W. He, H. Ren, G. Luo, Nitric Oxide Biol Chem 2018, 73, 1; b) J. Lee, C. Yang, S. Ahn, Y. Choi, K. Lee, Enhanced NO-induced angiogenesis via NO/H2S co-delivery from self-assembled nanoparticles. Biomater Sci 2021, 9, 5150.

[14] C. Dezfulian, E. Kenny, A. Lamade, A. Misse, N. Krehel, C. St Croix, E. E. Kelley, T. C. Jackson, T. Uray, J. Rackley, P. M. Kochanek, R. S. B. Clark, H. Bayir, J Neurochem 2016, 139, 419.

[15] Y. F. Lin, K. Raab-Graham, Y. N. Jan, L. Y. Jan, Proc Natl Acad Sci U S A 2004, 101, 7799.

[16] a) J. Pansiot, G. Loron, P. Olivier, R. Fontaine, C. Charriaut-Marlangue, J. C. Mercier, P. Gressens, O. Baud, PLoS ONE 2010, 5, e10916; b) M. Kajimoto, M. Nuri, J. R. Sleasman, K. A. Charette, B. R. Nelson, M. A. Portman, J Thorac Cardiovasc Surg 2021, 161, e485.

[17] T. J. Diao, X. Chen, L. H. Deng, H. X. Chen, Y. Liang, X. D. Zhao, Q. H. Wang, W. S. Yuan, B. C. Gao, Y. Ye, World J Gastroenterol 2012, 18, 3310.

[18] a) K. Imai, T. Kotani, H. Tsuda, Y. Mano, T. Nakano, T. Ushida, H. Li, R. Miki, S. Sumigama, A. Iwase, A. Hirakawa, K. Ohno, S. Toyokuni, H. Takeuchi, T. Mizuno, A. Suzumura, F. Kikkawa, Free Radic Biol Med 2016, 91, 154; b) S. Li, M. Fujino, N. Ichimaru, R. Kurokawa, S. Hirano, L. Mou, S. Takahara, T. Takahara, X. K. Li, Sci Rep 2018, 8, 14019.

[19] a) J. Wu, R. Wang, D. Yang, W. Tang, Z. Chen, Q. Sun, L. Liu, R. Zang, Biochem. Biophys. Res. Commun. 2018, 495, 2462; b) H. Li, O. Chen, Z, Ye, R. Zhang, H, Hu, N. Zhang, J. Huang, W. Liu, X. Sun, Biochem Pharmacol 2017, 130, 83.

[20] a) Q. Li, P. Yu, Q. Zeng, B. Luo, S. Cai, K. Hui, G. Yu, C. Zhu, X. Chen, M. Duan, X. Sun, Neurochemical Research 2016, 41, 2655; b) W. Yao, X Lin, X Han, L Zeng, A. Guo, Y. Guan, Z. Hei, J. Liu, P. Huang, Biosci Rep 2020, 40, BSR20191043; c) C. S. Huang, T. Kawamura, X. Peng, N. Tochigi, N. Shigemura, T. R. Billiar, A. Nakao, Y. Toyoda, Biochem Biophys Res Commun 2011, 408, 253; d) A. Kimura, K. Suehiro, A. Mukai, Y. Fujimoto, T. Funao, T. Yamada, T. Mori, J Thorac Cardiovasc Surg 2021, 5223, 00762.

[21] X. Wu, X. Li, Y. Liu, N. Yuan, C. Li, Z. Kang, X. Zhang, Y. Xia, Y. Hao, Y. Tan, Brain Res. 2018, 1698, 89.

[22] X. Bai, S. Liu, L. Yuan, Y. Xie, T. Li, L. Wang, X. Wang, T. Zhang, S. Qin, G. Song, L. Ge, Z. Wang, Brain Res. 2016, 1646, 410.

[23] a) S. D. Wang, Y. Y. Fu, X. Y. Han, Z. J. Yong, Q. Li, Z. Hu, Z. G. Liu, Neurochem Res 2021, 46, 866; b) Y. Zhou, X. H. Liu, S. D. Qu, J. Yang, Z. W. Wang, C. J. Gao, Q. J. Su, Chin. Med. J. 2013, 126, 3897; c) C. C. Lee, S. C. Chen, S. C. Tsai, B. W. Wang, Y. C. Liu, H. M. Lee, K. G. Shyu, J Biomed Sci 2006, 13, 143.

[24] G. Huang, J. Diao, H. Yi, L. Xu, J. Xu, W. Xu, Redox Biol 2016, 10, 108.

[25] X. Yin, X. Wang, Z. Fan, C. Peng, Z. Ren, L. Huang, Z. Liu, K. Zhao, J Cardiovasc Pharmacol Ther 2015, 20, 428.

[26] T. Oyaizu, M. Enomoto, N. Yamamoto, K. Tsuji, M. Horie, T. Muneta, I. Sekiya, A. Okawa, K. Yagishita, Sci. Rep. 2018, 8, 1288.

[27] J. L. Oakes, B. P. O'Connor, L. A. Warg, R. Burton, A. Hock, J. Loader, D. LaFlamme, J. Jing, L. Hui, D. A. Schwartz, I. V. Yang, Am. J. Resp. Cell Mol. Biol. 2013, 48, 27.

[28] T. Qiu, Z. S. Wang, X. H. Liu, H. Chen, J. Q. Zhou, Z. Y. Chen, M. Wang, G. J. Jiang, L. Wang, G. Yu, L. Zhang, Y. Shen, L. Zhang, L. He, H. X. Wang, W. J. Zhang, Exp Ther Med 2017, 13, 1948.

[29] W. Xiao, H. Tang, M. Wu, Y. Liao, K. Li, L. Li, X. Xu, Biosci. Rep. 2017, 37.

[30] a) D. Ma, M. Hossain, A. Chow, M. Arshad, R. M. Battson, R. D. Sanders, H. Mehmet, A. D. Edwards, N. P. Franks, M. Maze, Ann. Neurol. 2005, 58, 182; b) A. D. Filev, D. N. Silachev, I. A. Ryzhkov, K. N. Lapin, A. S. Babkina, O. A. Grebenchikov, V. M. Pisarev, Brain Sci 2021, 11, 889.

[31] J. Lavaur, D. Le Nogue, M. Lemaire, J. Pype, G. Farjot, E. C. Hirsch, P. P. Michel, J. Neurochem. 2017, 142, 14.

[32] T. Peng, G. L. Britton, H. Kim, D. Cattano, J. Aronowski, J. Grotta, D. D. McPherson, S. L. Huang, CNS Neuroscience and Therapeutics 2013, 19, 773.

[33] D. Ma, T. Lim, J. Xu, H. Tang, Y. Wan, H. Zhao, M. Hossain, P. H. Maxwell, M. Maze, J Am Soc Nephrol 2009, 20, 713.

[34] Q. Li, C. Lian, R. Zhou, T. Li, X. Xiang, B. Liu, Heart Lung Circul. 2013, 22, 276.

[35] a) F. Ulbrich, K. Kaufmann, M. Roesslein, F. Wellner, V. Auwarter, J. Kempf, T. Loop, H. Buerkle, U. Goebel, PLoS ONE 2015, 10, e0143887; b) F. Ulbrich, T. Lerach, J. Biermann, K. B. Kaufmann, W. A. Lagreze, H. Buerkle, T. Loop, U. Goebel, J Neurochem 2016, 138, 859.

[36] H. Qi, L. Soto-Gonzalez, K. A. Krychtiuk, S. Ruhittel, C. Kaun, W. S. Speidl, A. Kiss, B. K. Podesser, S. Yao, K. Markstaller, K. U. Klein, V. Tretter, Shock 2018, 49, 556.

[37] a) Q. de Roux, F. Lidouren, A. Kudela, L. Slassi, M. Kohlhauer, E. Boissady, M. Chalopin, G. Farjot, C. Billoet, P. Bruneval, B. Ghaleh, N. Mongardon, R. Tissier, Int J Mol Sci 2021, 22, 3257; b) J. H. Abraini, B. Kriem, N. Balon, J. C. Rostain, J. J. Risso, Anesth Analg 2003, 96, 746.

[38] Y. Li, P. Zhang, Y. Liu, W. Liu, N. Yin, Behav Brain Res 2016, 314, 165.

[39] M. Flick, M. Albrecht, G. Oei, R. Steenstra, R. P. Kerindongo, C. J. Zuurbier, H. H. Patel, M. W. Hollmann, B. Preckel, N. C. Weber, Eur J Pharmacol 2016, 791, 718.

[40] N. C. Weber, J. M. Schilling, M. V. Warmbrunn, M. Dhanani, R. Kerindongo, J. Siamwala, Y. Song, A. E. Zemljic-Harpf, M. J. Fannon, M. W. Hollmann, B. Preckel, D. M. Roth, H. H. Patel, Int J Mol Sci 2019, 20.

[41] P. S. Pagel, J. G. Krolikowski, P. F. Pratt, Jr., Y. H. Shim, J. Amour, D. C. Warltier, D. Weihrauch, Anesth Analg 2008, 107, 769.

Table 3. Neuroprotective Mechanisms of Carbon Monoxide (CO)

Abbreviations:

Akt, protein kinase B; AIF1, allograft inflammatory factor 1; AMPKα, adenosine monophosphate-activated protein kinase α subunit; Bax, BCL2 Associated X: a protein coding gene regulating apoptosis; Bcl2, B-cell lymphoma 2: a protein coding gene regulating apoptosis; BV2 cell line, a type of microglial cell derived from C57/BL6 murine that was immortalized by v-raf /v-myc carrying J2 retrovirus; CaMKKβ, Ca^{2+}/calmodulin-dependent protein kinase kinase β; cGMP, cyclic guanosine monophosphate; CHOP, C/EBP homologous protein; CO, carbon monoxide; CORM, carbon monoxide releasing molecule; CREB, cAMP response element-binding protein; elF2, eukaryotic initiation factor 2; eNOS, endothelial nitric oxide synthase; ERK1/2, extracellular regulated kinase 1/2; ERRα, estrogen-related receptor alpha; ERS, endoplasmic reticulum stress; GFAP, glial fibrillary acidic protein; GRP78, glucose-regulated protein 78; HBMEC, human brain microvascular endothelial cells; HIF1α, hypoxia-inducible factor 1-alpha; HO-1, heme-oxygenase 1; HSF1, heat shock factor 1: a transcription factor; Hsp-70 or 90, heat shock protein 70 KD or 90 KD; IL, interleukin; I/R, ischemia/reperfusion; ICAM-1, intercellular adhesion molecule 1; IRE1, the ER-resident transmembrane kinase-endoribonuclease inositol-requiring enzyme 1; Kv2.1, potassium voltage gated channel, Shab-related subfamily, member 1; L-type Ca^{2+} channels, long-lasting calcium channels; MAP2, microtubule-associated protein 2; MAPK, mitogen-activated protein kinase; MCAO, middle cerebral artery occlusion; MEK, MAP Kinase/ERK Kinase or mitogen-activated protein kinase kinase; mitoKATP, mitochondrial adenosine triphosphate-dependent potassium channel; MMP, matrix metalloproteinase; mTOR, the mammalian target of rapamycin: a kinase; NAMPT, nicotinamide phosphoribosyl transferase 1; NF-κB, nuclear factor kappa-light-chain-enhancer of activated B cells; NO, nitric oxide; nNOS, neuronal nitric oxide synthase; Nrf2, the nuclear factor erythroid 2–related factor 2; NSCs, neural stem cells; OGD, oxygen-glucose deprivation; p-p38, phosphorylated-p38 mitogen-activated protein kinase; pCREB, phosphorylated CREB; PERK, the protein kinase R (PKR)-like endoplasmic reticulum kinase; PI3K, phosphoinositide 3-kinase; PGC1α, peroxisome proliferator-activated receptor gamma coactivator 1α; PKG, cyclic GMP–dependent protein kinase; Ras, Ras (rat sarcoma) proteins belonging to small GTPase family; RGC, retinal ganglion cells; RISK, the reperfusion injury salvage kinase; ROS, reactive oxygen species; SCI, spinal cord injury; sGC, soluble guanylyl cyclase; SIRT1, sirtuin 1;TBI, traumatic brain injury; TNFα, tumor necrotic factor α; VEGF, vascular endothelial growth factor; and XBP1, the X-box binding protein 1: a transcription factor.

Citations:

[1] Y. K. Choi, C. K. Kim, H. Lee, D. Jeoung, K. S. Ha, Y. G. Kwon, K. W. Kim, Y. M. Kim, J Biol Chem 2010, 285, 32116.

[2] M. L. Dallas, J. P. Boyle, C. J. Milligan, R. Sayer, T. L. Kerrigan, C. McKinstry, P. Lu, J. Mankouri, M. Harris, J. L. Scragg, H. A. Pearson, C. Peers, FASEB J. 2011, 25, 1519.

[3] N. Dreyer-Andersen, A. S. Almeida, P. Jensen, M. Kamand, J. Okarmus, T. Rosenberg, S. D. Friis, A. Martínez Serrano, M. Blaabjerg, B. W. Kristensen, T. Skrydstrup, J. B. Gramsbergen, H. L. A. Vieira, M. Meyer, PLoS ONE 2018, 13, e0191207.

[4] N. Imuta, O. Hori, Y. Kitao, Y. Tabata, T. Yoshimoto, T. Matsuyama, S. Ogawa, Antioxid Redox Signal 2007, 9, 543.

[5] C. C. Lin, C. C. Yang, L. D. Hsiao, S. Y. Chen, C. M. Yang, Front Mol Neurosci 2017, 10, 387.

[6] H. L. Vieira, C. S. Queiroga, P. M. Alves, J Neurochem 2008, 107, 375.

[7] A. Horvat, N. Vardjan, R. Zorec, Curr Pharm Des 2017, 23, 4969.

[8] J. Biermann, W. A. Lagreze, C. Dimitriu, C. Stoykow, U. Goebel, Invest. Ophthalmol. Vis. Sci. 2010, 51, 3784.

[9] Y. Han, W. Yi, J. Qin, Y. Zhao, J. Zhang, X. Chang, Neurosci. Lett. 2015, 585, 126.

[10] F. Ulbrich, K. B. Kaufmann, A. Meske, W. A. Lagrèze, M. Augustynik, H. Buerkle, C. C. Ramao, J. Biermann, U. Goebel, PLoS ONE 2016, 11, e0165182.

[11] F. Ulbrich, C. Hagmann, H. Buerkle, C. C. Romao, N. Schallner, U. Goebel, J. Biermann, J Neuroinflammation 2017, 14, 130.

[12] B. Wang, W. Cao, S. Biswal, S. Dore, Stroke 2011, 42, 2605.

[13] R. Wang, Z. Chen, J. Wu, F. Xia, Q. Sun, A. Sun, L. Liu, Mol. Med. Rep. 2018, 17, 1297.

[14] G. Zheng, F. Zheng, Z. Luo, H. Ma, D. Zheng, G. Xiang, C. Xu, Y. Zhou, N. Tian, Y. Wu, T. Zhang, W. Ni, S. Wang, H. Xu, X. Zhang, Front. Pharmacol. 2020, 11, 761.

[15] Y. K. Choi, T. Maki, E. T. Mandeville, S. H. Koh, K. Hayakawa, K. Arai, Y. M. Kim, M. J. Whalen, C. Xing, X. Wang, K. W. Kim, E. H. Lo, Nat Med 2016, 22, 1335.

[16] Y. K. Choi, J. H. Kim, D. K. Lee, K. S. Lee, M. H. Won, D. Jeoung, H. Lee, K. S. Ha, Y. G. Kwon, Y. M. Kim, Antioxid. Redox Signal. 2017, 27, 20.

[17] S. Kaiser, L. Selzner, J. Weber, N. Schallner, GLIA 2020, 68, 2427.

[18] K. Lu, W. J. Wu, C. Zhang, Y. L. Zhu, J. Q. Zhong, J. Li, Curr Neurovasc Res 2020.

[19] N. Schallner, C. C. Romao, J. Biermann, W. A. Lagreze, L. E. Otterbein, H. Buerkle, T. Loop, U. Goebel, PLoS ONE 2013, 8, e60672.

[20] J. Stifter, F. Ulbrich, U. Goebel, D. Bohringer, W. A. Lagreze, J. Biermann, PLoS ONE 2017, 12, e0188444.

[21] C. S. Queiroga, S. Tomasi, M. Widerøe, P. M. Alves, A. Vercelli, H. L. Vieira, PLoS ONE 2012, 7, e42632.

[22] G. Zheng, Y. Zhan, H. Wang, Z. Luo, F. Zheng, Y. Zhou, Y. Wu, S. Wang, Y. Wu, G. Xiang, C. Xu, H. Xu, N. Tian, X. Zhang, EBioMedicine 2019, 40, 643.

Table 4. Neuroprotective Mechanisms of Xenon (Xe)

Abbreviations:

Akt, protein kinase B that was originally identified as the oncogene in the transforming retrovirus, AKT8; Ar, argon; ATM, standard atmosphere, unit of pressure, equal to the mean atmospheric pressure at sea level (i.e., 14.7 pounds per square inch; it is used to describe relative pressure); Bax, BCL2 Associated X: a protein coding gene regulating apoptosis; Bcl2, B-cell lymphoma 2: a protein coding gene regulating apoptosis; BDNF, brain-derived neurotrophic factor; CREB, cAMP response element-binding protein; DA, dopamine; E, embryonic day; ERK, extracellular regulated kinase; HIF1α, hypoxia-inducible factor 1-alpha; Irf1, interferon regulatory factor 1; LDH, lactate dehydrogenase; MAPK, mitogen-activated protein kinase; MCAO, middle cerebral artery occlusion; NMDA, N-methyl-D-aspartate; OGD, oxygen-glucose deprivation; p-Akt, phosphorylated Akt; PC12, a cell line derived from a pheochromocytoma of the rat adrenal medulla, with an embryonic origin from

the neural crest (a mixture of neuroblastic cells and eosinophilic cells); PDC, l-trans-pyrrolidine-2,4-dicarboxylic acid: an synthetic analog of glutamate; p-ERK, phosphorylated ERK; PI3K, phosphoinositide 3-kinase; PND, postnatal day; TBI, traumatic brain injury; TREK1, Twik-related K^+ channels (TREK) 1; Twik, tandem of P domains in weakly inward rectifier K^+ channels; Xe, xenon; Xe-ELIP, Xe-containing echogenic liposomes.

Citations:

[1] K. Harris, S. P. Armstrong, R. Campos-Pires, L. Kiru, N. P. Franks, R. Dickinson, Anesthesiology 2013, 119, 1137.

[2] M. Koziakova, K. Harris, C. J. Edge, N. P. Franks, I. L. White, R. Dickinson, Br J Anaesth 2019, 123, 601.

[3] J. Lavaur, D. Le Nogue, M. Lemaire, J. Pype, G. Farjot, E. C. Hirsch, P. P. Michel, J. Neurochem. 2017, 142, 14.

[4] C. Petzelt, P. Blom, W. Schmehl, J. Muller, W. J. Kox, Life Sci. 2003, 72, 1909.

[5] C. Petzelt, P. Blom, W. Schmehl, J. Müller, W. J. Kox, BMC Neurosci 2004, 5, 55.

[6] V. Limatola, P. Ward, D. Cattano, J. Gu, F. Giunta, M. Maze, D. Ma, Neuroscience 2010, 165, 874.

[7] S. Liu, Y. Yang, M. Jin, S. Hou, X. Dong, J. Lu, W. Cheng, J Neurol Sci 2016, 368, 277.

[8] T. Peng, G. L. Britton, H. Kim, D. Cattano, J. Aronowski, J. Grotta, D. D. McPherson, S. L. Huang, CNS Neuroscience and Therapeutics 2013, 19, 773.

[9] Y. W. Yang, W. P. Cheng, J. K. Lu, X. H. Dong, C. B. Wang, J. Zhang, L. Y. Zhao, Z. F. Gao, Br J Anaesth 2014, 113, 168.

[10] L. Zhuang, T. Yang, H. Zhao, A. R. Fidalgo, M. P. Vizcaychipi, R. D. Sanders, B. Yu, M. Takata, M. R. Johnson, D. Ma, Crit Care Med 2012, 40, 1724.

[11] A. D. Filev, D. N. Silachev, I. A. Ryzhkov, K. N. Lapin, A. S. Babkina, O. A. Grebenchikov, V. M. Pisarev, Brain Sci 2021, 11, 889.

[12] D. Ma, M. Hossain, A. Chow, M. Arshad, R. M. Battson, R. D. Sanders, H. Mehmet, A. D. Edwards, N. P. Franks, M. Maze, Ann. Neurol. 2005, 58, 182.

[13] Y. Luo, D. Ma, E. Ieong, R. D. Sanders, B. Yu, M. Hossain, M. Maze, Anesthesiology 2008, 109, 782.

Section S4: Supporting Information

Abbreviations, Acronyms, and Nomenclatures in the Text

Akt: protein kinase B that is the oncogene in the transforming retrovirus, AKT8

AMPKα: 5'-AMP-activated protein kinase catalytic subunit alpha-1

ARDS: acute respiratory distress syndrome

ATA: atmospheres absolute (1 ATA = 14.7 pounds per square inch that is the average atmospheric pressure exerted at sea level; it is used to describe the total ambient pressure on the system)

ATM: standard atmosphere, unit of pressure, equal to the mean atmospheric pressure at sea level (i.e., 14.7 pounds per square inch; it is used to describe relative pressure: e.g., 33' of water is 1 ATM of water but it is 2 ATA because of the 1 atmosphere of air above it)

BACH1: BTB and CNC homology 1 or basic leucine zipper transcription factor 1

Bax: Bcl-2-associated X protein

BBB: the blood-brain barrier

Bcl-2: B cell lymphoma 2

Bcl-xL: B-cell lymphoma-extra large

BSCB: the blood-spinal cord barrier

CA: the cornu ammonis

Cav-1 and Cav-3: caveolin-1 and caveolin-3

CDC: centers for disease control and prevention

C/EBP homologous protein: the UPR-regulated CCAAT/enhancer-binding protein (C/EBP) homologous protein

CF: concentration factor (CF = theoretical distribution/actual distribution)

cGMP: cyclic guanosine monophosphate

c-Myc: cellular Myc, with homology to an oncogene of the Avian virus, Myelocytomatosis, v-Myc

COPD: chronic obstructive pulmonary disease

CORM: carbon monoxide-releasing molecule

COVID-19: the coronavirus disease 2019

CREB: cAMP response element-binding protein

CSE: cystathionine γ-lyase, the endogenous biosynthetic enzyme of H_2S

CST: the corticospinal tract

CXCL: cysteine-X-cysteine motif chemokine ligand

d/o: day(s) old

d.p.i.: day(s) post injury

DTDP: 2,2′-dithiodipyridine

ELISA: enzyme-linked immunosorbent assay

eNOS: endothelial nitric oxide synthetase

ER: endoplasmic reticulum

ERK: extracellular signal-regulated kinase

ERRα: estrogen-related receptor alpha

FDA: food and drug administration

FOXG1: forkhead box G1

Fyn: a proto-oncogene tyrosine-protein kinase that belongs to the Src family kinases

GAP-43: growth associated protein 43

GRP78: the 78-kDa glucose-regulated protein

GSK-3: glycogen synthase kinase 3

GSK3β: glycogen synthase kinase 3β

HBO: hyperbaric oxygen

HIBI: hypoxic-ischemic brain injury

HIF-1α: hypoxia inducible factor-1α

hiPSCs: human inducible pluripotent stem cells

HK II: hexokinase II

HMGB1: high-mobility group box protein 1

HMOX1: a human gene encoding for the heme oxygenase 1

HO: heme oxygenase

HSF-1: heat shock factor 1

HSP: heat shock protein

HSP-70: heat shock protein 70

HSP-90: heat shock protein 90

i.a.: intraarterial

ICAM-1: intercellular adhesion molecule-1

IL: interleukin

iNOS: inducible nitric oxide synthetase

Interleukin: IL

IRE1: inositol-requiring enzyme-1

Irf1: interferon regulatory factor 1

IRG1: immune responsive gene 1

IRI: ischemia-reperfusion injury

i.t.: intrathecal

i.v.: intravenous

LC: lethal concentration

LSP: lipopolysaccharides

$_{L}$-trans-2,4-PDC: $_{L}$-trans-pyrrolidine-2,4-dicarboxylic acid

m/o: month(s) old

MACO: middle cerebral artery occlusion

MAP2: microtubule associated protein 2

MAPK: the mitogen-activated protein kinase

MBP: myelin basic protein

MDA: malondialdehyde

MEK-1/2: mitogen-activated protein kinase kinase-1/2

MeSH: medical subject headings

MGT: medical gas therapy

MIP-1α: macrophage inflammatory protein 1

Mitophagy: selective degradation of mitochondria by autophagy.

MPa: megapascal pressure unit

mPTP: the mitochondrial permeability transition pore

mTOR: mechanistic target of rapamycin for cell proliferation

NADH-TR: nicotinamide adenine dinucleotide dehydrogenase-tetrazolium reductase

NF-κB: nuclear factor-κB

NIH: the national institutes of health

NMDA: N-methyl-D-aspartate

nNOS: neuronal nitric oxide synthetase

NOS: nitric oxide synthetase

NPAS2: neuronal PAS (prostate specific antigen) domain protein 2

NQO1: NAD(P)H quinone oxidoreductase 1

Nrf2: nuclear factor erythroid 2-related factor 2

OGD: oxygen-glucose deprivation

pAkt: phosphorylated Akt (phospho-Akt)

PDE5A: phosphodiesterase-5A that specifically degrades cGMP, an important signaling molecule

p.i.: post injury

p38MAPK: p38 mitogen-activated protein kinases

p-p38MAPK: phosphorylated p38 mitogen-activated protein kinases

Parkin: an E3 ubiquitin ligase

PBN: N-tert-butyl-a-phenylnitrone

PD: Parkinson’s disease

pERK: phosphorylated ERK

PERK: protein kinase R (PKR)-like ER kinase

PGC-1α: peroxisome proliferators-activated receptor (PPAR) gamma coactivator 1-alpha that induces the expression of several members of the mitochondrial ROS detoxification system

PGC-1α: peroxisome proliferator-activated receptor gamma coactivator 1-alpha

PI3K: phosphoinositide 3-kinases

PINK1: PTEN-induced kinase 1 (PTEN: phosphatase and tensin homolog deleted on chromosome 10)

PKG: protein kinase G

PND: postneonatal day

pO_2: partial pressure of oxygen

ppm: parts per million

Ras: a class of small GTPase

RISK: the reperfusion injury salvage kinase

RNS: reactive nitrogen species

ROS: reactive oxygen species

rRT-PCR: real-time reverse transcription polymerase chain reaction

scRNA-seq: single-cell RNA (ribonuclear acid) sequencing

SDF-1: stromal cell-derived factor-1α and -1β

SDF-1α: stromal cell derived factor-1α

SIRT1: NAD-dependent protein deacetylase sirtuin-1

S-nitrosylation: the addition of one NO group to the thiol side chain of cysteine residues within a protein or a peptide to form an *S*-nitrosothiol

SOD: superoxide dismutase

Stat3: signal transducer and activator of transcription 3

Tau: a microtubule-associated protein that normally stabilizes neuronal microtubules

TGF- β1: transforming growth factor- β1

TLRs: toll-like receptors, a class of receptors that play a key role in regulating the innate immune system

TNFα: tumor necrosis factor-α

TNFα: tumor necrosis factor-α

tPA: tissue plasminogen activator

VEGF-B: vascular endothelial growth factor-B

vol: volume

XBP1: X-box binding protein 1

Xe-ELIP: Xe-containing echogenic liposomes

y/o: year(s) old
